# Termite sanitary behaviour and their associated bacteria protect against latent *Xylaria* and entomopathogenic *Metarhizium*

**DOI:** 10.3897/imafungus.17.211888

**Published:** 2026-10-01

**Authors:** Er-Fu Yang, Samantha C. Karunarathna, Milan C. Samarakoon, Itthayakorn Promputtha, Qian-Qian Tao, Chun-I Chiu, Zhen-Xiong Zhao, Xiu-Juan Luo, Martin M. Gakuubi, Steven L. Stephenson, Abdallah M. Elgorban, Alanoud T. Alfagham, Nakarin Suwannarach, Entaj Tarafder, Jing-Ya Yang, Fu-Qiang Yu, Dong-Qin Dai, Mei-Mei Wang, Saowaluck Tibpromma

**Affiliations:** 1 Key Laboratory of Yunnan Provincial Department of Education of the Deep-Time Evolution on Biodiversity from the Origin of the Pearl River, Qujing Normal University, Qujing, Yunnan 655011, China Key Laboratory of Yunnan Provincial Department of Education of the Deep-Time Evolution on Biodiversity from the Origin of the Pearl River, Qujing Normal University Qujing China; 2 Department of Biology, Faculty of Science, Chiang Mai University, Chiang Mai 50200, Thailand Department of Biology, Faculty of Science, Chiang Mai University Chiang Mai Thailand; 3 Yunnan Key Laboratory for Fungal Diversity and Green Development & Yunnan International Joint Laboratory of Fungal Sustainable Utilization in South and Southeast Asia, Germplasm Bank of Wild Species, Kunming Institute of Botany, Chinese Academy of Sciences, Kunming, Yunnan 650201, China Yunnan Key Laboratory for Fungal Diversity and Green Development & Yunnan International Joint Laboratory of Fungal Sustainable Utilization in South and Southeast Asia, Germplasm Bank of Wild Species, Kunming Institute of Botany, Chinese Academy of Sciences Kunming China; 4 Center for Yunnan Plateau Biological Resources Protection and Utilization, College of Biology and Food Engineering, Qujing Normal University, Qujing, Yunnan 655011, China Center for Yunnan Plateau Biological Resources Protection and Utilization, College of Biology and Food Engineering, Qujing Normal University Qujing China; 5 Department of Entomology and Plant Pathology, Faculty of Agriculture, Chiang Mai University, Chiang Mai 50200, Thailand Department of Entomology and Plant Pathology, Faculty of Agriculture, Chiang Mai University Chiang Mai Thailand; 6 State Key Laboratory of Environmental Criteria and Risk Assessment, Chinese Research Academy of Environmental Sciences, Beijing 100012, China State Key Laboratory of Environmental Criteria and Risk Assessment, Chinese Research Academy of Environmental Sciences Beijing China; 7 Department of Agriculture, Earth and Environmental Sciences, Faculty of Science, Mwenge Catholic University, P.O. Box 1226, Moshi, Tanzania Department of Agriculture, Earth and Environmental Sciences, Faculty of Science, Mwenge Catholic University Moshi Tanzania; 8 Department of Biological Sciences, University of Arkansas, Fayetteville, Arkansas 72701, USA Department of Biological Sciences, University of Arkansas Fayetteville United States of America; 9 Center of Excellence in Biotechnology Research (CEBR), King Saud University, Riyadh 11451, Saudi Arabia Center of Excellence in Biotechnology Research (CEBR), King Saud University Riyadh Saudi Arabia; 10 Department of Botany and Microbiology, College of Science, King Saud University, Riyadh 11451, Saudi Arabia Department of Botany and Microbiology, College of Science, King Saud University Riyadh Saudi Arabia; 11 Center of Excellence in Microbial Diversity and Sustainable Utilization, Chiang Mai University, Chiang Mai 50200, Thailand Center of Excellence in Microbial Diversity and Sustainable Utilization, Chiang Mai University Chiang Mai Thailand; 12 College of Agronomy and Biological Science, Yuxi Normal University, Yuxi, Yunnan 653100, China College of Agronomy and Biological Science, Yuxi Normal University Yuxi China; 13 Yunnan Ecological Economy Society, Kunming, Yunnan Province 650000, China Yunnan Ecological Economy Society Kunming China

**Keywords:** Burial behaviour, culturable microbes, entomopathogens, fungistatic bacteria, fungus-growing termites, weedy fungi

## Abstract

Fungus-growing termites harbour diverse microbial communities in their combs and guts. However, culture-based surveys, cross-colony and cross-regional comparisons and assessments of their antimicrobial activities against entomopathogens and fungal weeds remain limited. Through culture-dependent isolation, we surveyed the bacterial and fungal diversity associated with the combs and guts of termites in the genera *Macrotermes* and *Odontotermes*, recovering 600 non-*Termitomyces* fungal isolates representing 79 genera, as well as 59 bacterial isolates. Pathogenicity tests on *Macrotermes gilvus* revealed that a *Metarhizium* isolate obtained from a healthy comb was highly virulent to termites. Combs incubated in the boxes yielded 112 isolates of the weed *Xylaria* and multi-locus phylogenetic analyses revealed high species diversity. Together, these findings revealed two major microbial threats within healthy termite fungus combs. First, highly virulent entomopathogens that directly threaten termite survival. Second, a diverse assemblage of comb-specialised xylariaceous fungi that persist latently and compete for comb nutrients. Behavioural assays revealed that termites exhibited sharply differential hygienic responses to fungi co-occurring in soil plates, aggressively burying *Metarhizium* regardless of comb presence, but responded weakly to co-evolved *Xylaria*. Newly-hatched termites also displayed innate avoidance of food contaminated with *Metarhizium* conidia. Several comb- and gut-associated bacterial isolates, as well as termite-generated soil boluses, inhibited both *Metarhizium* and *Xylaria in vitro*. This study underscores the utility of culture-based approaches for elucidating microbial diversity and ecology in termite nests and highlights the roles of host behaviour and comb- and gut-associated bacteria in suppressing both fungal weed invasions and entomopathogen infections.

## Introduction

Fungus-growing termites (*Isoptera*, *Termitidae*, *Macrotermitinae*) are a well-known example of insect fungiculture (Biedermann and Vega 2020; Li et al. 2021). Within the highly obligate symbiotic farming system of *Macrotermitinae*-*Termitomyces*, termite workers actively maintain and renew the garden substrates (Rouland-Lefèvre 2010). Older workers forage for woody and leaf litter, which young workers then process and ingest along with fungal nodules, specialised nutrient-rich structures that contain the asexual propagules of *Termitomyces* (Li et al. 2017). These spore-laden materials pass through the termite gut, where they are partially digested by gut-associated symbiotic bacteria and the resulting faecal matter is deposited to form fresh fungus combs (Murphy et al. 2021; Ahmad et al. 2022). Over time, these combs mature within the stable microenvironment of subterranean nests or epigeal mounds, providing optimal conditions for the development of fungal nodules, which serve as a nutrient-rich food source for colony members (Vesala et al. 2023). Finally, those old combs become a primary food source for older workers, whose faecal matter is deposited in specialised refuse areas (Leuthold et al. 1989; Li et al. 2017; Shoaib et al. 2024). The fungus comb also provides a favourable environment for undesirable microbial colonisation. However, our understanding of the entomopathogens and fungal weeds that threaten this stable and efficient cultivation system, as well as the defensive mechanisms by which termites and their symbiotic microbes counteract such invasions, remains insufficient.

In addition to the primary *Termitomyces* cultivars, culture-dependent and culture-independent molecular analyses have demonstrated that this favourable environment also supports a diverse array of non-*Termitomyces* fungi and bacteria (Guedegbe et al. 2009; Otani et al. 2014, 2019; Agarwal et al. 2024; Shoaib et al. 2024). Gut bacteria analyses across nine fungus-growing species of termites spanning five genera (*Ancistrotermes*, *Macrotermes*, *Microtermes*, *Odontotermes* and *Pseudacanthotermes*) revealed that gut communities harbour representatives of 26 bacterial phyla and are dominated by *Bacteroidetes*, *Firmicutes*, *Proteobacteria*, *Spirochaetes* and *Synergistetes* (Otani et al. 2014). Nanopore sequencing revealed 418 unique fungal genera in the fungus combs (both fresh and decaying), alates, workers and nymphs of *Odontotermes obesus* (Agarwal et al. 2024). Based on culture-dependent investigations, several bacterial genera have been identified, including *Actinomadura*, *Bacillus*, *Burkholderia*, *Micromonospora*, *Pseudomonas* and *Streptomyces* (Benndorf et al. 2018; Shoaib et al. 2024), while fungal genera predominantly belonging to *Aspergillus*, *Didymostilbe*, *Fusarium*, *Trichoderma*, *Geotrichum*, *Paecilomyces*, *Pleurostoma*, *Pseudallescheria* and *Scedosporium* have also been recovered (Sibanda et al. 2019; Nel et al. 2021; Yang et al. 2025). Those non-*Termitomyces* microbes are likely introduced via soil contact, extensive termite foraging and nutrient-rich fungus combs that attract environmental microorganisms (Goes and Adams 2025). Previous research suggested that the obligate association with *Termitomyces* has forced the gut bacterial communities of the fungus-growing termites towards a relatively uniform composition (Otani et al. 2014; Barcoto et al. 2020). High-throughput studies have shown that gut bacterial communities of *Macrotermes michaelseni* and *Odontotermes* sp. are nearly identical (Makonde et al. 2015). Since all comb material passes through termite guts, it is inevitable that gut bacteria are also deposited in the comb (Otani et al. 2016). Indeed, clone library analyses have shown that termite guts harbour both higher abundance and greater bacterial diversity than fungus combs (Long et al. 2010; Makonde et al. 2015). Despite the documented richness of fungal communities in combs and guts, a substantial portion of the microbial assemblages inhabiting the termite comb and gut remains undiscovered or inadequately characterised (Ahmad et al. 2025).

Once the fungus combs are compromised or abandoned, termite-specialised *Xylaria* (TE clade) species rapidly colonise the ecological niche previously occupied by termite-cultivated *Termitomyces*. As such, they are considered typical fungal weeds in comb-*Termitomyces* cultivation (Visser et al. 2011; Katariya et al. 2017; Panchal et al. 2025). Termites employ specific behaviour to remove, isolate and suppress the growth of the fungal weed *Xylaria*, primarily by encasing them with soil boluses containing fungistatic microbes (Panchal et al. 2025). Although *Xylaria* species are regarded as weedy fungi, termites failed to effectively suppress their spread in soil-free laboratory assays; indeed, they elicited significantly lower bolus-burial rates than did *Aspergillus* and *Trichoderma* (Yang et al. 2025). Moreover, comb- or gut-associated microbes exhibited stronger *in vitro* antagonistic activity against *Aspergillus* than against *Xylaria*. However, because these findings were derived from a single termite colony and the entomopathogenic potential of *Aspergillus* was not assessed (Yang et al. 2025), it remains unresolved whether termites exhibit distinct or preferential behavioural responses to entomopathogenic fungi compared to co-evolved weed *Xylaria* (Yang et al. 2025). Furthermore, numerous *Xylaria* species and putative novel taxa have been reported from fungus combs (Yue et al. 2013; Wangsawat et al. 2021; Li et al. 2025; Okane et al. 2025). Most of those *Xylaria* isolates do not produce fruiting bodies or spores under laboratory conditions (Li et al. 2025), leaving their dispersal strategy and life cycle across colonies and generations largely unknown. Specifically, how *Xylaria* enters the nest and persists in the comb remains an unresolved question, as *Xylaria* species are rarely detected in surrounding soils (Thomas 1987).

Although entomopathogenic fungi, including *Beauveria*, *Metarhizium* and *Isaria*, have occasionally been detected in fungus-growing termite combs (Otani et al. 2019; Agarwal et al. 2024), there is limited evidence that these entomopathogens proliferate extensively within the nest systems of fungus-growing termites. Therefore, how these systems maintain stability in the presence of soil-borne entomopathogenic threats warrants further investigation. Entomopathogenic fungi of the genus *Metarhizium* are widespread, soil-borne facultative saprotrophs and important pathogens of termites and other insects (Shah and Pell 2003; Keppanan et al. 2018; Correa et al. 2022; Zheng et al. 2024). *Metarhizium anisopliae* is highly infective and causes mortality in fungus-growing termites, including *Macrotermes michaelseni*, *Microcerotermes diversus*, *Odontotermes obesus* and *O. formosanus* (Dong et al. 2009; Mburu et al. 2009; Balachander et al. 2013; Cheraghi et al. 2013). In response to this infection threat, termites exhibit social immunity that restricts the transmission of *M. anisopliae* amongst healthy colony members (Cremer et al. 2007). This defence is mediated by a suite of collective behaviours, including vibrational alarm signalling, aggregation, allogrooming, defecation and burial of infected individuals (Rosengaus et al. 1998; Myles 2002; Yanagawa and Shimizu 2007; Sun et al. 2017; Ferreira et al. 2025). In addition, subterranean termites construct nests from faecal materials that may contain antifungal compounds produced by gut endosymbionts, thereby enhancing colony-level defence (Chouvenc et al. 2009). Burial of pathogen-infected cadavers within nest material can further stimulate the activity of protective bacteria, such as *Streptomyces* species, thereby strengthening antifungal defence and maintaining colony hygiene (Chouvenc et al. 2013; Nakashima and Matsuura 2025). Soldiers of *Nasutitermes corniger* secrete defensive compounds from their frontal glands that inhibit *M. anisopliae* infection (Ferreira et al. 2025). Beyond bacterially mediated and behavioural defences, comb-associated fungi, such as *Trichoderma* and *Xylaria*, have also been reported to inhibit *Beauveria* and *Metarhizium* in dual-culture assays (Wen et al. 2020; Nagam et al. 2021).

Herein we contribute to our understanding of how termite colonies maintain disease-free conditions within their mounds and nests when confronted with two principal groups of microbial threats — fungal weeds that compromise the cultivated *Termitomyces* symbiont and soil-borne entomopathogens that endanger the termite hosts themselves. We characterised the culturable fungal and bacterial communities associated with the guts and combs of fungus-growing termites belonging to the genera *Macrotermes* and *Odontotermes*, with a special focus on screening for culturable entomopathogenic fungi and evaluating their pathogenicity to the termite host. In addition, we assessed the diversity of latent, weedy *Xylaria* across different termite genera and locations, as well as their potential for transmission. Building on previous findings, we hypothesised that termites exhibit more pronounced burying responses to entomopathogens than to comb weedy fungi and that their comb- and gut-associated fungistatic bacteria display antagonistic activity against these two categories of microbial invaders.

## Materials and methods

### Sample collection

**Figure 1.**
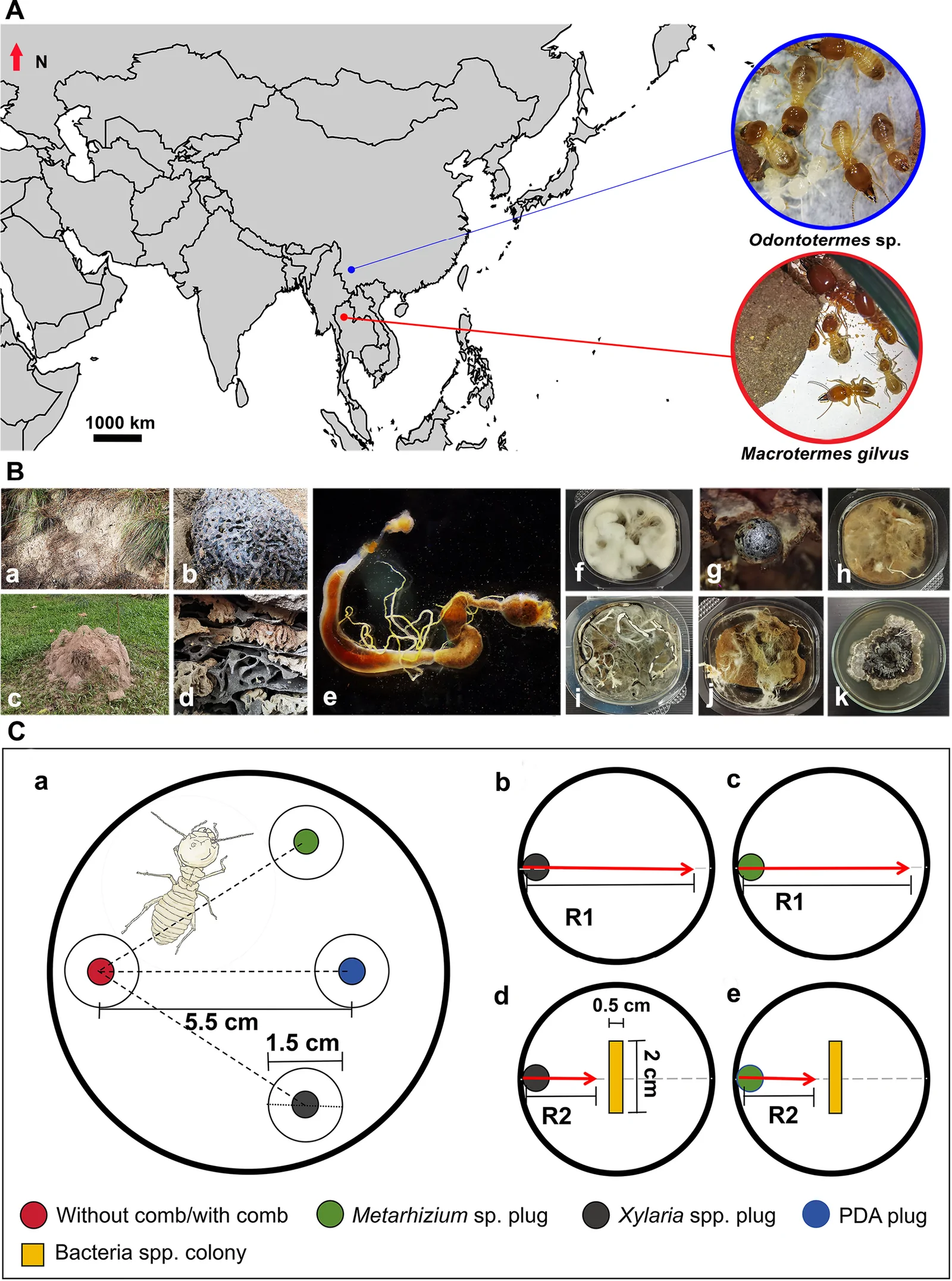
Sample collection and experimental methods. **A** Geographic map of sampling sites. The blue dot indicates the sampling location in Chuxiong (China), where *Odontotermes* sp. colonies were collected; the red dot indicates the sampling location in Chiang Mai (Thailand), where *Macrotermes gilvus* colonies were collected; **B** Microbial isolation and *Xylaria* induction. **a**, **b** Subterranean nest and fungus comb of *Odontotermes* sp. termites. **c**, **d** Epigeous mound of *Macrotermes gilvus*. **e** Worker gut dissection. **f**–**j***Xylaria* morphotypes emerging from incubated 2-g inner fungus-comb subsamples after 14 days. **k** A representative *Xylaria* isolate cultured on PDA; **C** Experimental design for evaluating termite responses to fungi and microbial interactions. **a** Assay setup presenting termites with a plug of the entomopathogenic fungus *Metarhizium* sp., a plug of the weedy fungus *Xylaria* and a PDA control plug, either with or without comb pieces. The weight of fresh soil boluses deposited on and within a 1.5-cm-diameter paper circle was recorded. **b–e** Dual-culture assays evaluating bacterial inhibition of *Metarhizium* and *Xylaria* growth.

Three colonies of *Odontotermes* sp. were sampled from Linggang Village, Daguokou Town, Chuxiong City, Yunnan Province, China (24°54'46"N, 101°09'30"E, elevation: 1871 m), in January 2025. Three colonies of *Macrotermes gilvus* were sampled from Chiang Mai University Campus, Chiang Mai, Thailand (18°48'20"N, 98°57'09"E; elevation: 300 m), in April 2025 (Fig. 1A). During sampling, gentle lateral pressure was applied to remove the outer mound casing, minimising disturbance of the fungus combs and termites (Lee and Lee 2015). Intact, clean fungus combs, together with termites, were transferred into temporary plastic boxes (15 cm × 10 cm × 6 cm) that had been sterilised with 75% ethanol and exposed to UV light for 30 min before use. Comb material from each colony was divided amongst three boxes lined with sterile, moist tissue paper. To maintain microbial integrity and minimise loss of microbial biodiversity, isolation was performed upon return to the laboratory, within 3 hours. Termites were identified, based on the morphological characteristics of major soldiers, according to published taxonomic keys (Tsai and Chen 1963; Ahmad 1965).

### Isolation of microbial communities from termite fungus combs

From each colony, 30 samples of inner portions of mature combs (5 g each) were collected, yielding a total of 180 samples across the six colonies of *Macrotermes gilvus* and *Odontotermes* sp. (Fig. 1B). Microbial isolation followed a dilution-and-plating approach (Guswenrivo et al. 2018; Yang et al. 2025), with minor modifications. Each 5 g sample was homogenised in 45 ml of sterile phosphate-buffered saline (PBS: 1.44 g Na_2_HPO_4_, 0.24 g NaH_2_PO_4_, 0.2 g KCl, 8 g NaCl, dissolved in 1 litre of distilled H_2_O), shaken for 1 min and then centrifuged at 4 °C and 1,000 rpm for 5 min. The resulting suspension was serially diluted to 10^–3^ by transferring 1 ml of the suspension into 9 ml of PBS at each step. Subsequently, a 10-µl aliquot of the 10^–3^ dilution was spread on to potato dextrose agar (PDA; without antibiotics) and nutrient agar (NA) plates using a sterile L-shaped spreader. Plates were then incubated at 27 °C for one week. Hyphal tips of fungi and small individual bacterial colonies were subcultured on to new PDA and NA plates, respectively (Yang et al. 2025). Bacterial strains isolated from combs of *Odontotermes* sp. were labelled “CX-Comb” (Suppl. material 1: table SS1), while those isolated from combs of *Macrotermes gilvus* were labelled “CMU-C” (Suppl. material 1: table S2); fungal strains isolated from combs of *Odontotermes* sp. were labelled “CX” (Colony 1: CX-CB; Colony 2: CX-CU; Colony 3: CX-CY) (Suppl. material 1: table S3) and those isolated from combs of *Macrotermes gilvus* were labelled “Cmu” (Colony 1: Cmu-a; Colony 2: Cmu-b; Colony 3: Cmu-c) (Suppl. material 1: table S4). All microbial isolation procedures were conducted in a laminar flow cabinet to prevent external contamination.

### Isolation of microbial communities from the guts of termite workers

Isolation of bacteria and fungi from termite guts followed the procedures described in previous publications with slight modifications (Um et al. 2013; Yang et al. 2025). Active termites (15 major workers and 15 minor workers) were randomly collected from each colony, yielding a total of 180 gut samples across the six colonies of *Macrotermes gilvus* and *Odontotermes* sp. Each termite was surface-sterilised by immersion in 75% ethanol for 1 min, followed by three rinses with sterile distilled water and air-drying for 1 min in a laminar flow hood. The whole gut (Fig. 1B) was aseptically dissected using flame-sterilised fine-tip forceps, transferred into 500 µl of phosphate-buffered saline (PBS) and gently rinsed. The resulting suspensions were evenly spread on to PDA and NA plates in triplicate using a sterile L-shaped spreader. Plates were incubated at 27 °C for one week. Emerging fungal hyphae and bacterial colonies were subcultured on to fresh PDA and NA plates, respectively, to obtain pure isolates (Yang et al. 2025). Bacterial strains isolated from *Odontotermes* sp. were labelled “CX-Gut” (Suppl. material 1: table SS1), while those isolated from *Macrotermes gilvus* were labelled “CMU-g” (Suppl. material 1: table S2); fungal strains isolated from *Odontotermes* sp. were labelled “CX” (Colony 1: CX-QJ, Colony 2: CX-JJ, Colony 3: CX-QQ) (Suppl. material 1: table S5), and those isolated from *Macrotermes gilvus* were labelled “Cmu” (Colony 1: Cmu-ag, Colony 2: Cmu-bg, Colony 3: Cmu-cg) (Suppl. material 1: table S6).

### Isolation of *Xylaria* spp. from incubated termite-fungus combs

To minimise contamination from external microbes, the outer portions of fresh termite fungus combs were discarded. To isolate comb-specialised *Xylaria*, inner portions of mature fungal combs were collected and incubated separately in sterile boxes containing moist sterile tissue. Fifty fragments of mature fungal combs (2 g each) were collected from each of five colonies — two of *Odontotermes* sp. (China) and three of *Macrotermes gilvus* (Thailand), resulting in a total of 250 comb samples. Each of these samples was incubated individually in a small, sterilised plastic box (4 × 3 × 5 cm; disinfected with 75% ethanol and exposed to UV light for 30 min prior to use), lined with a layer of moist sterile tissue paper to maintain appropriate humidity. After two weeks of incubation in the dark at room temperature, visible *Xylaria* strains were isolated by transferring both actively growing aerial mycelia and sclerotial tissues on to PDA plates (Fig. 1B). *Xylaria* strains from *Odontotermes* sp. were labelled “CX” and those from *Macrotermes gilvus* were labelled “CMU” (Suppl. material 1: table S7).

To assess whether incipient colonies established under laboratory conditions showed indications of weedy *Xylaria* invasion, alates were collected from *Macrotermes gilvus* colonies from Thailand in May 2025 and paired. The pairs were placed in plastic boxes containing sterile agar (wiped with 75% ethanol and exposed to UV for 30 min) and maintained for five months with sterile food and sterile water. The food was obtained from the same natural colonies and autoclaved three times at 121 °C for 20 min each. Afterwards, all kings, queens and newly-hatched offspring were removed from the boxes (five replicates). The termite-excluded boxes were then incubated at 27 °C for one month to observe whether visible *Xylaria* mycelia emerged.

### DNA extraction, PCR amplification and sequencing

Fungal mycelia harvested from two-week-old cultures were transferred into 1.5 ml microcentrifuge tubes for genomic DNA extraction. Fungal DNA was isolated using the Biospin Fungus Genomic DNA Extraction Kit (BioFlux®, Hangzhou, China) following the manufacturer’s instructions. Bacterial DNA was extracted from biomass collected from NA plates using the Biospin Bacteria Genomic DNA Extraction Kit (BioFlux®, Hangzhou, China) according to the manufacturer’s protocol. PCR amplification was performed in a 25 μl reaction mixture containing 12.5 μl of 2× Power Taq PCR MasterMix (Sangon, China), 8.5 μl of sterile double-distilled water, 2 μl of DNA template and 1 μl each of forward and reverse primers (10 pmol). The targeted loci included the 16S ribosomal RNA (16S rRNA) gene for bacterial isolates, the internal transcribed spacer (ITS) region for fungal isolates and the alpha-actin gene (*ACT*), beta-tubulin 2 (*TUB2*) and DNA-dependent RNA polymerase second largest subunit (*RPB2*) for isolates of *Xylaria* (see Table 1 for primer details). The PCR conditions consisted of an initial denaturation at 94 °C for 4 min, followed by 30 cycles at 94 °C for 30 s, annealing at gene-specific temperatures (see Table 1) for the indicated durations and 72 °C for 1 min, with a final extension at 72 °C for 10 min. PCR products were sequenced by Sangon Biotech Co., Ltd. (Shanghai, China) and the resulting sequences were deposited in GenBank (Suppl. material 1: tables S1–S7).

**Table 1.** Primers, target loci and PCR cycling conditions used for amplification of bacterial and fungal genes in the present study.

**Locus**	**Primer**	**Primer DNA sequence (5'**–**3')**	**Annealing Temp**.	**References**
ITS	ITS4	TCCGTAGGTGAACCTGCGG	55 °C, 50 s	White et al. (1990)
	ITS5	CCTCCGCTTATTGATATGC		
16S rRNA	fD1	AGAGTTTGATCCTGGCTCAG	50 °C, 1 min	Weisburg et al. (1991)
	rP3	TACGGYTACCTTGTTACGACTT		
*ACT*	512F	ATGTGCAAGGCCGGTTTCGC	56 °C, 50 s	Carbone and Kohn (1999)
	783R	TACGAGTCCTTCTGGCCCAT		
*TUB2*	T1	AACATGCGTGAGATTGTAAGT	52 °C, 50 s	O’Donnell and Cigelnik (1997)
	T22	TCTGGATGTTGTTGGGAATCC		
*RPB2*	5F	GAYGAYMGWGATCAYTTY GG	57 °C, 55 s	Liu et al. (1999)
	7cR	CCCATRGCTTGYTTRCCCAT		

### Phylogenetic analyses

Raw sequences obtained from the sequencing company were assembled and edited using Geneious (Restricted) (https://www.geneious.com; accessed 27 April 2026). All ITS and 16S gene sequences obtained were subjected to BLASTn searches against the GenBank database (http://blast.ncbi.nlm.nih.gov/; accessed 27 April 2026) to determine the genus-level identification and record the most similar strains (Suppl. material 1: tables S1–S6). A four-locus phylogenetic analysis (ITS, *ACT*, *RPB2* and *TUB2*) was conducted to determine: (1) whether multiple species of *Xylaria* can co-exist within a single colony and (2) whether clade differentiation is associated with the termite genus or geographic location (Suppl. material 1: table S7). For bacterial isolates, phylogenetic analyses were conducted using 16S rRNA sequences, including both our sequences and relevant outgroup reference sequences (Suppl. material 1: tables S1, S2). Multiple sequence alignments were generated using the web-based MAFFT server (https://mafft.cbrc.jp/alignment/server/; accessed 27 April 2026) (Katoh and Standley 2013) and manually checked in BioEdit 7.2.3 (Hall 1999). Ambiguous or uninformative regions were trimmed using trimAL v.1.2 (http://trimal.cgenomics.org; accessed 27 April 2026), followed by sequence assembly by BioEdit. The Alignment Transformation Environment (ALTER) digital platform was used to convert FASTA files to PHYLIP format (Glez-Peña et al. 2010). Maximum Likelihood (ML) phylogenetic analyses were conducted using RAxML-HPC2 on XSEDE (v.8.2.12) via the CIPRES Science Gateway v.3.3 (Miller et al. 2010), applying the GTRGAMMA substitution model with 1,000 bootstrap replicates (Stamatakis 2014). Phylogenetic trees were visualised in FigTree v.1.4.0 and further edited using iTOL v.7 (https://itol.embl.de/; accessed 27 April 2026) and node support values were shown based on ML bootstrap analyses.

### Preparation of conidial suspensions and virulence assessment *in vitro*

The *Metarhizium* sp. Cmu-b60 isolate, obtained from a *Macrotermes gilvus* comb, produced a high yield of conidia on PDA and showed high sequence similarity to *M. anisopliae* strain 090904-2-3, based on ITS BLASTn analysis (Suppl. material 1: table S4). In contrast, *Metarhizium* sp. CX-CU12 isolated from *Odontotermes* sp. comb showed a high level of similarity to *Metarhizium lepidiotae* BUM66.1 (Suppl. material 1: table S3). *Metarhizium anisopliae* is the most commonly used and studied entomopathogenic species (Zimmermann 2007). Consequently, *Metarhizium* sp. Cmu-b60 was selected for the entomopathogenicity test. Conidial suspensions were prepared for bioassays against the host termite *Macrotermes gilvus*. Conidia were harvested from one-month-old, actively sporulating cultures, suspended in sterile distilled water containing 0.02% Tween 80 and then passed through a sterile filter to remove hyphal debris (Wang and Powell 2004; Singha et al. 2011). Conidial density was quantified with a haemocytometer under a compound microscope and the suspension was subsequently diluted with sterile 0.02% Tween 80 carrier solution to obtain a working concentration of 1 × 10^8^ conidia ml^–1^. Conidial viability was verified on PDA before testing; only suspensions exhibiting a germination rate of > 95% were used for virulence assays. Control treatments consisted of a sterile carrier solution containing 0.02% Tween 80 without conidia.

To evaluate the virulence of *Metarhizium* sp. strain Cmu-b60 on fungus-growing termites, active individuals were collected from *Macrotermes gilvus* colony 2, the colony from which strain Cmu-b60 was originally isolated. Each experimental group consisted of 10 major workers, 10 minor workers, two major soldiers and two minor soldiers. Each group was placed on a piece of moistened tissue paper in 90-mm glass Petri plates (oven-sterilised at 180 °C for 3 h). Ten replicate groups were prepared, and each termite was gently guided using sterile wooden sticks and briefly dipped in a 1 × 10^8^ conidia ml^–1^ conidial suspension for 5 s (Syazwan et al. 2021). Control groups were handled the same way and dipped in sterile distilled water containing 0.02% Tween 80 (Singha et al. 2011). The plates were partially sealed with parafilm and maintained in the dark at 27 °C. The plates were monitored at 24-h intervals. Individuals that failed to respond to gentle stimulation, such as prodding the antennae and palps with a fine pin, were recorded as dead, removed from the plates and transferred to fresh Petri dishes containing moistened tissue to observe subsequent fungal colonisation and mycosis development. Mortality was quantified for each 24-h interval and survival rates were calculated using the formula:

$$\text{ Survival rate }=\frac{N_{\text{alive }}}{N_{\text{total }}}\times 100\%$$

where N_alive_ represents the number of surviving individuals at each observation and N_total_ is the initial group size.

### Behavioural responses of termites towards weedy *Xylaria* and entomopathogenic *Metarhizium*

To assess the differences in termite burying responses to the entomopathogenic fungus *Metarhizium* sp. and the weedy fungus *Xylaria* sp. in the presence or absence of the termite-fungus garden, a behavioural experiment was conducted in sterilised 90 mm glass Petri dishes (oven-sterilised at 180 °C for 3 h before use). Termites were simultaneously exposed to a PDA control plug, a *Metarhizium* sp. plug and a *Xylaria* sp. plug (Fig. 1C). Soil used in the assays was collected from mounds of *Macrotermes gilvus* colonies and adjacent to nests of *Odontotermes* sp. The soil was air-dried at room temperature, autoclaved five times at 121 °C for 20 min and subsequently oven-dried at 60 °C prior to use. A piece of sterile, moistened tissue paper was placed in each of the 90-mm glass Petri dishes and 10 g of treated soil was evenly spread over the surface. *Metarhizium* sp. Cmu-b60 was selected because it shared high ITS BLASTn similarity to *M. anisopliae* and exhibited strong virulence against termites; *Xylaria* sp. clades 11 and 20 were selected because they were frequently induced from comb-incubation assays of their respective termite hosts (see Results). Therefore, on one side of the Petri plate (0.5 cm from the edge), either a 0.2 g fresh comb (collected within 30 min) with four attached *Termitomyces* nodules (treatment) or no comb (control) was placed on a sterile circular filter paper (1.5 cm in diameter) (Fig. 1C). On the opposite side (5.5 cm from the centre), three 0.5-cm-diameter, one-week-old agar plugs of *Metarhizium* sp. (Cmu-b60), *Xylaria* spp. (clade 11 or clade 20) or PDA were placed on sterile circular filter paper (1.5 cm in diameter), as shown in Fig. 1C-a (Yang et al. 2025). Prior to transferring termites to the comb-soil plates, termites were acclimatised for 15 min in Petri dishes at 27 °C in a dark, quiet environment to allow allogrooming. Each Petri plate then received one minor soldier, five major workers and five minor workers. The plates were partially sealed with parafilm to allow ventilation and incubated at 27 °C in a dark, quiet environment for 6 hours. Nine replicates were prepared for each termite genus. To quantify the extent to which termites exhibited hygienic behaviour, the weight of fresh soil boluses on and within 1.5-cm-diameter filter paper was measured in each treatment. To ensure analytical precision, the weight of the soil boluses was determined by weighing by difference using the equation:

$$W_{\text{soil boluses }}=W_{\mathrm{PDA}+\text{ soil boluses }}-W_{\mathrm{PDA}}$$

(Katariya et al. 2017; Yang et al. 2025).

To evaluate whether burying responses affect fungal growth, four 0.5-cm-diameter, one-week-old fungal plugs grown on PDA were placed on water agar (WA) plates as controls. In contrast, *Metarhizium* sp. and *Xylaria* spp. plugs collected 24 hours after the burying assay, along with the attached soil boluses, were transferred on to separate WA plates. All inoculation points were positioned at the four corners, each 2 cm from the edge of the Petri dish. After one week, Growth Inhibition (GI) was calculated using the formula:

$$\mathrm{GI}(\%)=\frac{R_{c}-R_{t}}{R_{c}}\times 100\%$$

where R_C_ is the maximum radial growth diameter of *Metarhizium* or *Xylaria* on WA control plates and R_t_ is the maximum radial growth diameter of fungi with encased soil boluses on the test WA plates, including submerged mycelia.

Moreover, to assess whether termites exhibit avoidance behaviour towards *Metarhizium*-contaminated food substrates, six laboratory-established colonies, each derived from paired alates and containing 20 new individuals (3 months old), were used. Termite food material was collected from natural *Macrotermes gilvus* colonies in September 2025 and sterilised by five rounds of autoclaving at 121 °C for 20 min each. The food material was then oven-dried prior to use. For the treatment, 1 g of food substrate was mixed with 1 ml of a *Metarhizium* sp. Cmu-b60 conidial suspension prepared as described above (1 × 10^8^ conidia ml^–1^) and placed as a triangular patch in one corner of a 90-mm glass Petri dish lined with moistened sterile filter paper. For the control, 1 ml of sterile distilled water containing 0.02% Tween 80 was used instead of the conidial suspension. Termites from each of the six incipient colonies were separately transferred to glass Petri dishes, lined with moistened sterile filter paper and provisioned with a cotton ball. Termites from each of the three incipient colonies were separately transferred to glass Petri dishes for the treatment group, and termites from another three colonies were used for the control group. The dishes were partially sealed with parafilm to maintain humidity while allowing ventilation. Mortality and the extent of food substrate displacement were recorded after 120 h (5 days).

### Co-culture assays of microbial interactions *in vitro*

This study also aimed to screen bacteria associated with fungus-growing termites for inhibitory activity against the entomopathogen *Metarhizium* sp. Cmu-b60 and the weedy fungus *Xylaria* sp. clade 20 (a fast-growing *Xylaria* clade isolated from incubated combs of both *Macrotermes* and *Odontotermes*). To determine whether bacterial strains inhibit the growth of *Metarhizium* sp. and *Xylaria* sp. *in vitro*, bacterial genera shared by *Macrotermes* (Thailand) and *Odontotermes* (China) were selected. For each genus, two bacterial strains were selected, representing different hosts and distinct clades in the 16S rRNA Maximum Likelihood (ML) phylogenetic tree (*Bacillus* sp. CX-gut6 and CMU-g10; *Brevibacillus* sp. CX-gut6-1 and CMU-C11; *Burkholderia* sp. CX-Comb6 and CMU-C7, *Enterobacter* sp. CX-gut10 and CMU-g3; *Pseudomonas* sp. CX-gut7 and CMU-g13; *Serratia* sp. CX-Comb4 and CMU-g5) (see Results; Suppl. material 1: tables S1, S2, Suppl. material 2: fig. S1; ). Five-millimetre-diameter mycelial plugs from one-month-old *Metarhizium* sp. Cmu-b60 and one-week-old *Xylaria* sp. (Clade 20) cultures were placed at the edge of 60- or 90-mm NA Petri dishes. A bacterial colony (0.5 cm wide and 2 cm long) was inoculated at the opposite side of the plate (Fig. 1C). Control plates were inoculated with the fungal isolates only and all the assays were performed in triplicate (Fig. 1C) (Mis et al. 2024). The plates were incubated at 27 °C for 5 d for the bacterium–*Xylaria* assay and for 15 d for the bacterium–*Metarhizium* assay. The inhibitory effects of the bacteria against the two fungi were expressed as percentage inhibition (PI) in radial growth, calculated using the formula:

$$\mathrm{PI}(\%)=\frac{R_{1}-R_{2}}{R_{1}}\times 100\%$$

where R_1_ is the radial growth distance of the fungus from the inoculation point to the opposite edge in the control plates and R_2_ is the minimum growth in the direction facing the bacterium in treatment plates (Mis et al. 2024).

### Diversity analysis and visualisation

Analyses of fungal and bacterial communities were performed using the R (v.4.5.1) (R Core Team 2025). Fungal isolates were identified by BLAST analysis of the ITS sequence against the NCBI database. Relative abundance of each genus was calculated per nest as the percentage of isolates assigned to that genus amongst all isolates obtained from that nest. A bar chart of mean relative abundances and a heatmap of the top 50 genera were generated using the R packages “microeco” and “pheatmap”, respectively (Liu et al. 2021). For the heatmap, abundance data were log_10_(x+0.001)-transformed and row-wise Z-score scaled, without column clustering. Alpha diversity indices (Shannon and Chao1) were compared between nest and gut samples within each termite species using Wilcoxon rank-sum tests. Beta diversity was assessed using Bray-Curtis distance matrices and visualised via Principal Coordinate Analysis (PCoA). Permutational multivariate analysis of variance (PERMANOVA) was performed to assess community dissimilarity, using 9999 permutations and the Bray–Curtis distance matrix as input (Bray and Curtis 1957). To identify genera with high discriminatory potential between nest and gut, a random forest (RF) classification model (ntree = 1000; the model was run 25 times) was performed using the randomForest package in R and the top 10 genera ranked by Gini importance were selected as discriminative features (Liaw and Wiener 2002). Importantly, to avoid confounding between termite species and geographic origin, all statistical comparisons (alpha diversity, beta diversity and differential abundance analyses) were performed separately for the two termite species.

In tests of termite burying responses to weedy *Xylaria* and entomopathogen in the presence or absence of termite comb, the normality assumption was met for *M. gilvus* and violated for *Odontotermes* sp. (Shapiro-Wilk: *P* = 0.258, *P* < 0.001) and the assumption of homogeneity of variance was violated for both species (Levene’s Test: *P* = 0.022, *P* = 0.004). Therefore, a Generalised Linear Model (GZLM) was fitted using a gamma distribution with a log link function and robust standard errors in IBM SPSS Statistics for Windows v. 29.0 (IBM Corp., Chicago, IL, USA). All main effects and interactions were evaluated using Type III *Wald χ^2^* statistics within the GZLM framework. When pairwise comparisons were required, simple-effects analyses were performed using estimated marginal means (EMMs) with a Bonferroni correction to control the family-wise Type I error rate.

For the analysis of whether deposited soil boluses inhibited the growth of *Xylaria* sp. and *Metarhizium* sp., the generated data met assumptions of normality and homogeneity of variance in SPSS (Shapiro-Wilk: *P* > 0.05, Levene’s Test: *P* > 0.05). Therefore, one-way ANOVA was applied; when the ANOVA indicated a statistically significant difference, post hoc pairwise comparisons were conducted using Tukey’s HSD test.

Likewise, for analysis of whether the termite entomopathogen (*Metarhizium* sp. Cmu-b60) and *Xylaria* sp. clade 20 were inhibited by 12 selected bacterial strains in SPSS, the generated data met the assumptions of normality and homogeneity of variance. Therefore, a one-way ANOVA was applied; if the ANOVA revealed statistically significant differences, post hoc pairwise comparisons were performed using Tukey’s HSD test.

Kaplan-Meier curves and survival plots were generated using Prism (GraphPad Prism v. 9.0; GraphPad Holdings, San Diego, CA, USA) (Motulsky 2003).

## Results

### Diversity and compositional profile of comb- and gut-associated bacterial isolates

**Figure 2.**
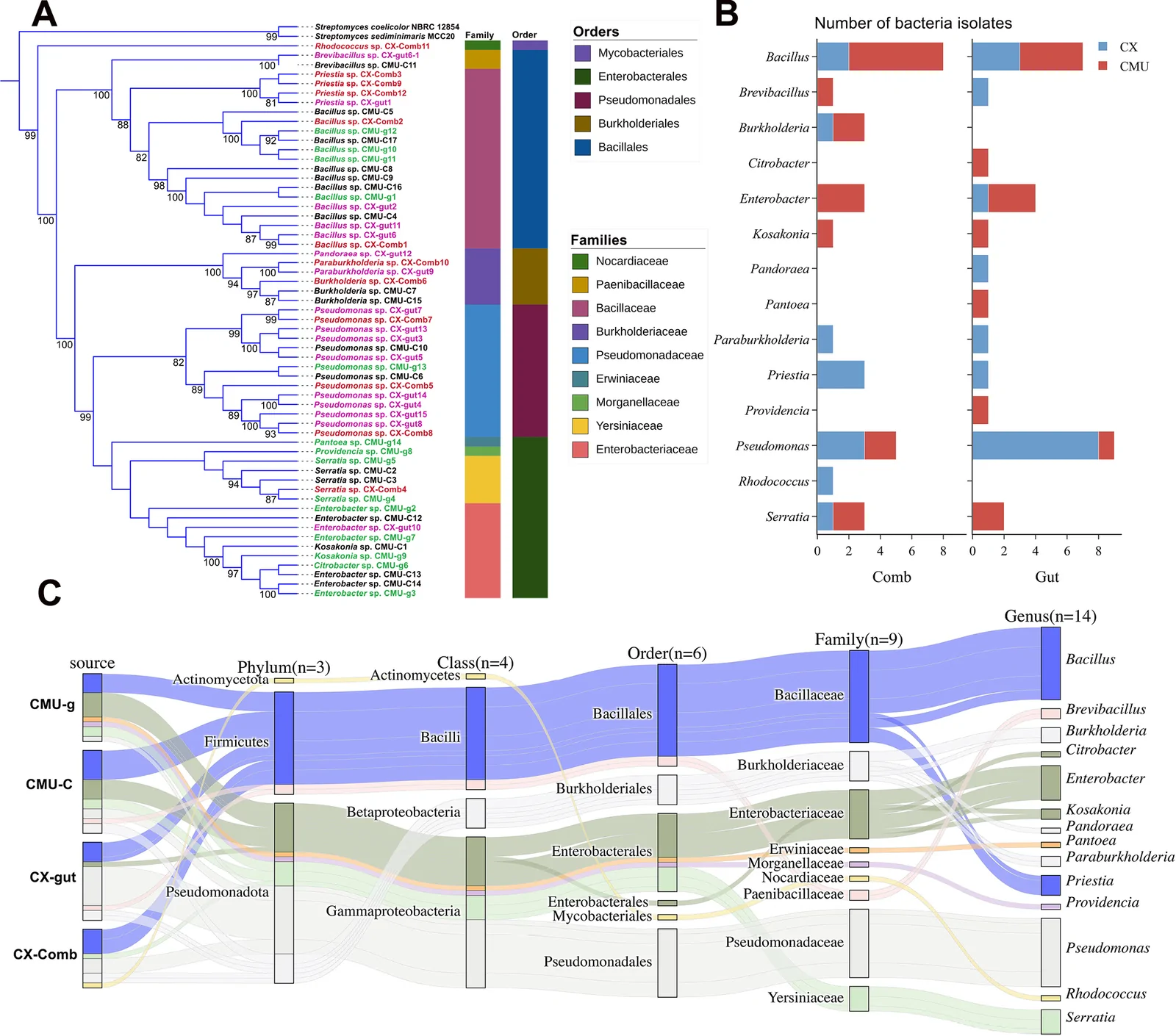
Diversity analysis of bacterial isolates from the combs and worker guts. **A** Maximum-Likelihood phylogenetic tree of the 59 isolates based on 16S rRNA gene sequences, with *Streptomyces coelicolor* NBRC 12854 and *S. sediminimaris* MCC20 included as outgroups. Nodes with ML bootstrap support values of 80% or greater are shown. The tree is colour-coded by source: CMU-C (black) indicates isolates from combs of *Macrotermes gilvus*; CMU-G (green) indicates isolates from guts of *M. gilvus* workers; CX-Comb (red) indicates isolates from combs of *Odontotermes* sp.; CX-gut (purple) indicates isolates from guts of Odontotermes workers; **B** Stacked bar plot summarising the number of bacterial isolates from each source; **C** Relative abundance of culturable bacteria associated with the guts and combs of two fungus-growing termites, from phylum to genus level.

In this study, 59 bacterial strains were isolated from six colonies of two species of termites. From *Odontotermes* sp. (China), 28 strains were isolated; 12 strains from inner combs and 16 strains from worker guts. From *Macrotermes gilvus* (Thailand), 31 bacterial strains were isolated; 17 strains from the inner combs and 14 strains from worker guts (Suppl. material 1: tables S1, S2; Suppl. material 2: fig. S1). The ML phylogenetic tree, based on 16S rRNA, revealed that the 59 strains belong to 14 distinct genera (Fig. 2A). Six genera — *Bacillus* (15 isolates: 25.4%), *Brevibacillus* (3.4%), *Burkholderia* (5.1%), *Enterobacter* (7 isolates: 11.9%), *Pseudomonas* (14 isolates: 23.7%) and *Serratia* (5 isolates: 8.5%) were common to both *Odontotermes* sp. and *Macrotermes gilvus*. In contrast, four genera: *Pandoraea* (1.7%), *Paraburkholderia* (3.4%), *Priestia* (6.8%) and *Rhodococcus* (1.7%), were exclusively isolated from *Odontotermes* sp., while *Citrobacter* (1.7%), *Pantoea* (1.7%), *Providencia* (1.7%) and *Kosakonia* (3.4%) were unique to *M. gilvus*. Furthermore, substantial overlap was observed between comb- and gut-associated isolates across the two termite species (Fig. 2).

At the family level, the 59 bacteria were distributed amongst nine distinct families. Six families (*Bacillaceae*, *Burkholderiaceae*, *Enterobacteriaceae*, *Paenibacillaceae*, *Pseudomonadaceae* and *Yersiniaceae*) were shared between *Odontotermes* sp. and *Macrotermes gilvus*. *Nocardiaceae* was exclusively associated with *Odontotermes* sp., whereas *Erwiniaceae* and *Morganellaceae* were unique to *M. gilvus*. Moreover, considerable overlap in family-level composition was observed between comb- and gut-associated bacterial isolates from the two termite species (Fig. 2B).

At the higher taxonomic level, the 59 bacterial isolates were classified into five distinct orders, four classes and three phyla. Four orders (*Bacillales*, *Burkholderiales*, *Enterobacterales* and *Pseudomonadales*) were shared between *Odontotermes* sp. and *Macrotermes gilvus*, but members of the *Mycobacteriales* were exclusively isolated from *Odontotermes* sp. (Fig. 2).

### Diversity and compositional profile of comb- and gut-associated fungal isolates

**Figure 3.**
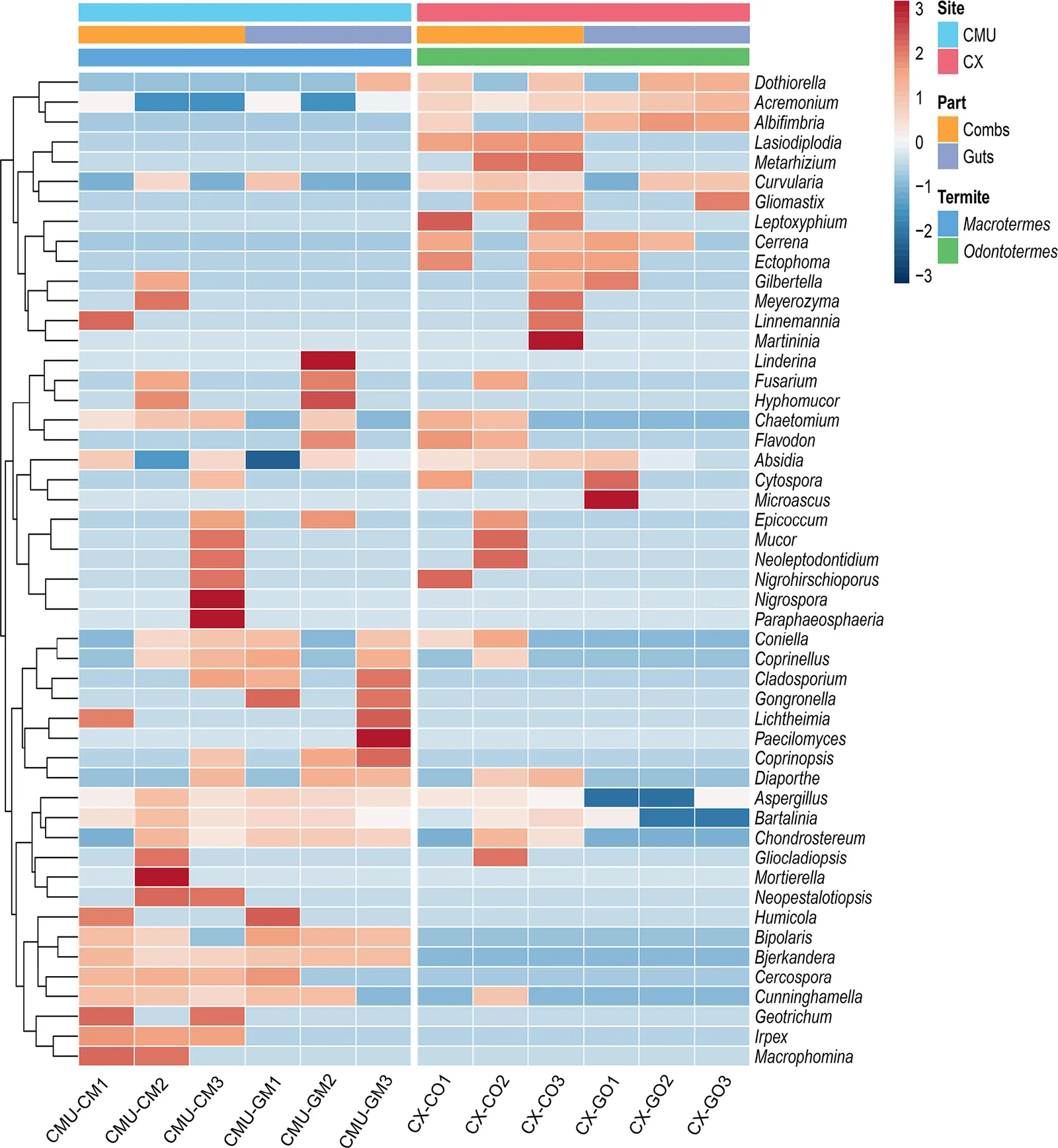
Heatmap showing the relative abundance of the 50 most common fungal genera isolated from termite combs and guts. Each column represents a single colony (pooled from 30 guts or 30 comb subsamples). Raw data are shown for each colony, rather than averaged values, to illustrate inter-colony variation. Three colonies were analysed per termite genus in each collection site. Abbreviations: CMU-CM: *M. gilvus* comb; CMU-GM: *Macrotermes gilvus* gut; CX-CO: *Odontotermes* sp. comb; CX-GO: *Odontotermes* sp. gut.

A total of 600 culturable fungal strains (excluding comb-specialised *Xylaria* isolates) were isolated from six colonies of two termite species. Of these, 300 fungal strains were from *Macrotermes gilvus*, with 200 strains associated with combs and 100 isolated from worker guts. Similarly, 300 strains were isolated from *Odontotermes* sp., 200 from the combs and 100 from worker guts (Suppl. material 2: figs S2–S5). Based on ITS sequences and BLASTn searches, the isolated fungal strains belong to three phyla — *Ascomycota* (413 isolates, 68.8%), *Mucoromycota* (171 isolates, 28.5%) and *Basidiomycota* (16 isolates, 2.7%). *Ascomycota* isolates were far more abundant in fungus combs than in guts (*Macrotermes*, 170 vs. 61; *Odontotermes*, 148 vs. 34). In contrast, *Mucoromycota* isolates were more frequent in guts than in combs (*Macrotermes*, 49 vs. 66; *Odontotermes*, 22 vs. 34). Furthermore, the isolated fungal strains were distributed into 11 classes, 26 orders, 52 families and 79 genera (Suppl. material 1: tables S3–S6). At the genus level, *Trichoderma* was the most dominant genus, accounting for 164 isolates (27.3% of all fungal isolates). This was followed by *Mucor* (9.8%), *Aspergillus* (5.5%), *Fusarium* (5.1%), *Linnemannia* (4.7%), *Curvularia* (3.5%), *Cunninghamella* (3.3%), *Penicillium* (3.2%), *Neopestalotiopsis* (3%) and *Umbelopsis* (2.8%) (Fig. 3; Suppl. material 1: tables S3–S6).

**Figure 4.**
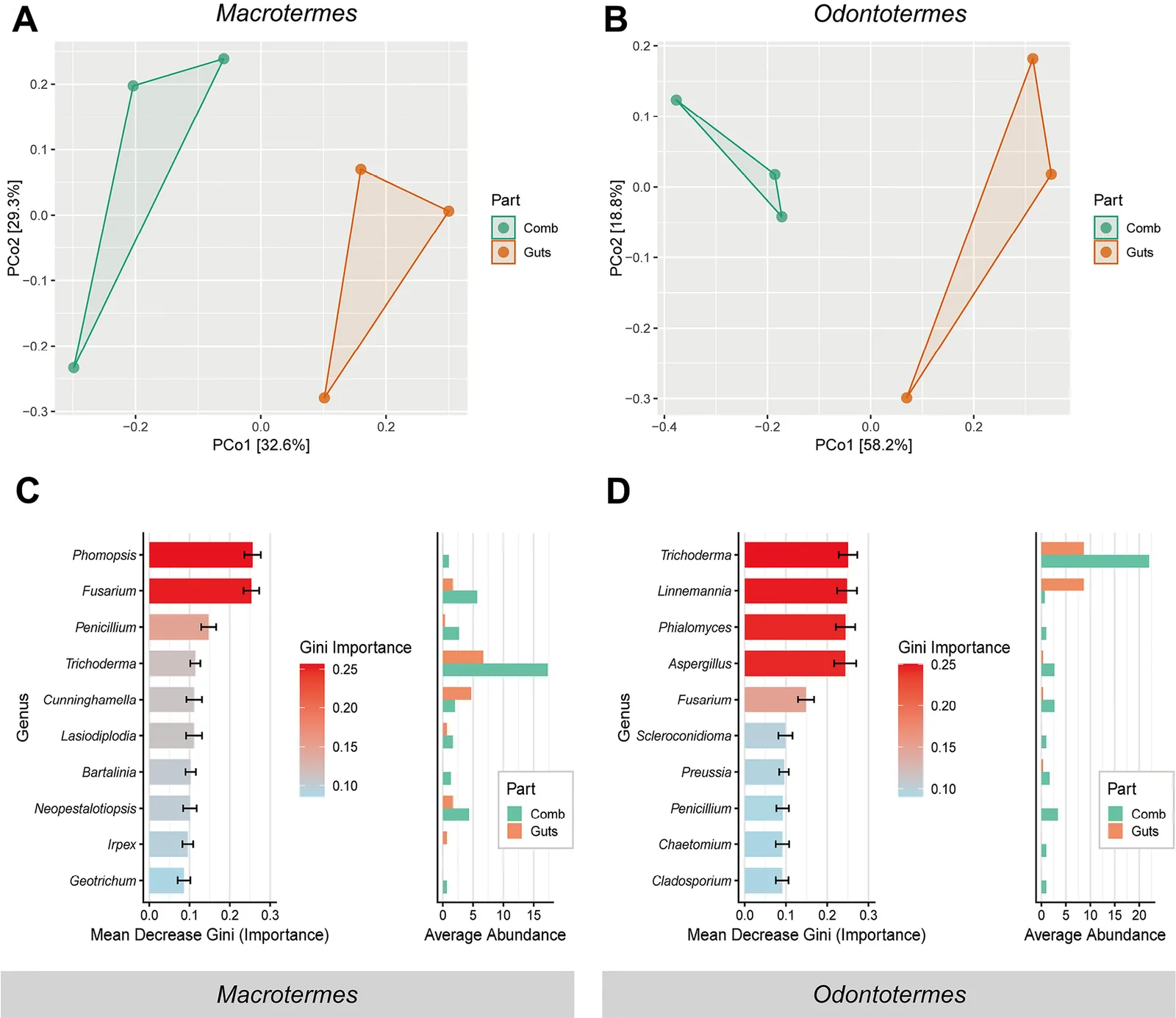
Fungal community structure and discriminatory taxa between combs and guts from *Macrotermes* and *Odontotermes*. **A, B** Principal coordinate analysis (PCoA) score plots; **C, D** Ranking and group abundance of the top ten discriminative genera identified by Random Forest analysis.

The relative abundance (%) of genera and phyla associated with four isolate sources (combs and worker guts of *Macrotermes gilvus*; combs and worker guts of *Odontotermes* sp.) is shown in Suppl. material 2: fig. S6. The top 50 dominant fungal genera are shown in Fig. 3. Alpha diversity indices (Shannon and Chao1) did not differ significantly between nest and gut samples within either termite species (Wilcoxon rank-sum test, *P* > 0.05; Suppl. material 2: fig. S7). Subsequent PERMANOVA analysis showed that the effect of sample part (comb vs. gut) on the fungal community structure showed a strong, but statistically non-significant trend in both termite species. For *Macrotermes gilvus*, it explained 30.2% of the variance (PERMANOVA, *F* = 1.736, P = 0.1, *R*^2^ = 0.302) (Fig. 4A). For *Odontotermes* sp., the explanatory power was even higher, accounting for 50.4% of the variance (*F* = 4.066, P = 0.1, *R*^2^ = 0.504) (Fig. 4B). A random forest (RF) analysis identified the key fungal genera that differed between termite guts and combs within each termite genus. In *Macrotermes* termites, *Bartalinia*, *Geotrichum* and *Phomopsis* were isolated exclusively from the combs, whereas *Irpex* was isolated only from worker guts (Fig. 4C). Amongst the genera shared between combs and guts, most exhibited higher abundance in the combs, with the exception of *Cunninghamella*. In *Odontotermes* termites, *Chaetomium*, *Cladosporium*, *Penicillium*, *Phialomyces* and *Scleroconidioma* were isolated only from the combs and, amongst the shared genera, most were more abundant in combs, except for *Linnemannia* (Fig. 4D). Overall, the cultivable fungal community composition did not differ significantly between gut and comb isolates from the same genus or location (Fig. 4).

Using the dilution plating technique, no *Termitomyces* strains were isolated from either worker guts or comb samples. One *Xylaria* strain (CX-CB35/GMBCC1340) was isolated and showed 100% ITS sequence similarity to the X*ylaria grammica* isolate Otu0315, as determined by BLASTn analysis. However, this isolate did not belong to the termite comb-specialised *Xylaria* (TE) clade. One strain (CX-CU12/ GMBCC1448) isolated from combs of *Odontotermes* sp. (China), showed 100% ITS sequence similarity to *Metarhizium lepidiotae* isolate BUM66.1, based on BLASTn analysis, while another strain Cmu-b60 isolated from combs of *Macrotermes gilvus* (Thailand), showed 99.6% ITS sequence similarity to *M. anisopliae* 090904-2-3, based on BLASTn analysis.

### Diversity of induced comb-specialised *Xylaria* spp.

A total of 112 strains of *Xylaria* were isolated from incubated combs of two fungus-growing termite genera; these were 50 strains from two colonies of *Odontotermes* sp. in China and 62 strains from three colonies of *Macrotermes gilvus* in Thailand (Suppl. material 1: table S7; Suppl. material 2: figs S8, S9). Multi-locus phylogenetic analyses were performed using sequences generated from four loci (ITS, *ACT*, *RPB2* and *TUB2*), together with sequences of 76 comb-specialised *Xylaria* taxa and outgroup species retrieved from GenBank (Li et al. 2025). Thus, a total of 188 termite-associated *Xylaria* strains were included in the phylogenetic reconstruction (Suppl. material 1: table S7). The dataset comprised ITS sequences for all strains, while *ACT*, *TUB2* and *RPB2* sequences were available for 175, 138 and 145 specimens, respectively. The aligned lengths of ITS, *ACT*, *RPB2* and *TUB2* were 670, 297, 1,183 and 1,784 characters, respectively, yielding 2,441 distinct alignment patterns. The proportion of gaps and completely undetermined characters accounted for 33.81% of the total alignment.

**Figure 5.**
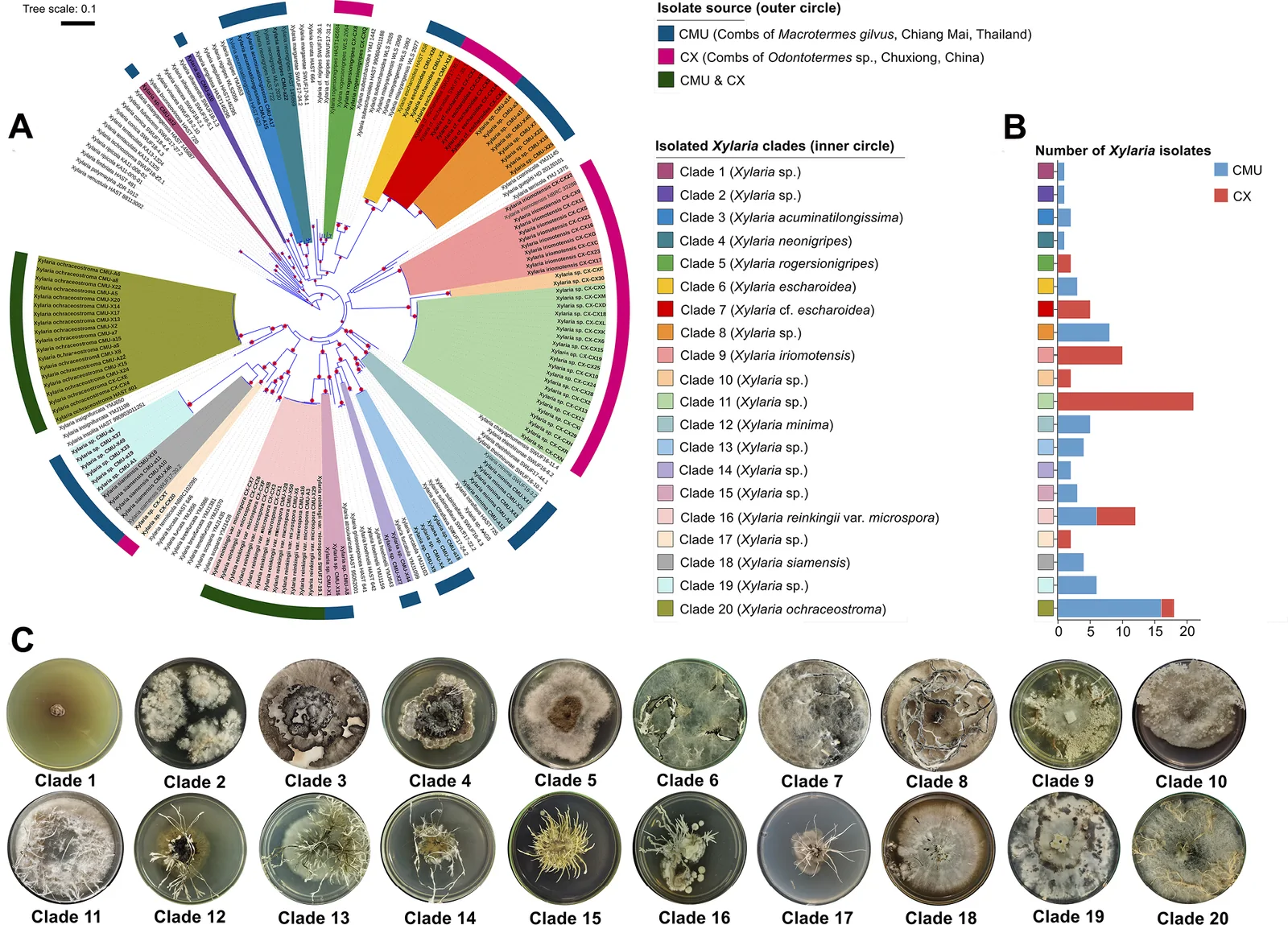
Phylogenetic relationships and cultural characteristics of *Xylaria* strains isolated from termite combs. **A** Circular phylogenetic tree illustrates the relationships amongst *Xylaria* strains induced from box-incubated combs of *Odontotermes* sp. from China (CX-: 50 strains) and *Macrotermes gilvus* from Thailand (CMU-: 62 strains). Nodes with statistical support ≥ 60 are indicated by red dots; **B** Bar chart showing the number of independent and overlapping clades between the two termite genera; **C** Representative colony morphologies of the 20 clades grown on PDA after a month.

Phylogenetic analysis, based on a concatenated four-locus dataset, resolved the 112 strains into 20 distinct operational taxonomic units (OTUs, hereafter referred to as clades). Ten clades corresponded to previously described species — clade 3 (*Xylaria acuminatilongissima*), clade 4 (*X. neonigripes*), clade 5 (*X. rogersionigripes*), clade 6 (*X. escharoidea*), clade 7 (*X. cf. escharoidea*), clade 9 (*X. iriomotensis*), clade 12 (*X. minima*), clade 16 (*X. reinkingii* var. *microspora*), clade 18 (*X. siamensis*) and clade 20 (*X. ochraceostroma*). The remaining ten clades (1, 2, 8, 10, 11, 13, 14, 15, 17 and 19) likely represent novel *Xylaria* taxa. Only two clades — clade 16 (*X. reinkingii* var. *microspora*) and clade 20 (*X. ochraceostroma*) — were isolated from both termite genera, whereas the remaining 18 clades were each restricted to either the Chinese (*Odontotermes* sp.) or Thai (*Macrotermes gilvus*) hosts (Fig. 5). In *Odontotermes* sp. combs, the four most frequently isolated were clade 11 (21 isolates), clade 9 (10 isolates), clade 16 (six isolates) and clade 7 (five isolates). In *Macrotermes gilvus* combs, the four most abundant clades were clade 20 (16 isolates), clade 8 (eight isolates), clades 16 and 19 (six isolates in each) and clade 12 (five isolates) (Fig. 5).

Notably, only clade 12 (*X. minima*) was observed to form miniature sclerotia (3–6 mm diam.; n = 5) on two-week incubated combs of *Macrotermes gilvus* (Suppl. material 2: fig. S10). Such structures were, however, not observed on *Odontotermes* combs. The sclerotia were globose, initially white and gradually darkened to black upon maturation (Suppl. material 2: fig. S10). Vertical sections revealed white internal tissue consisting of densely entangled mycelia, with no spores or conidia observed (Suppl. material 2: fig. S10). The sclerotia were directly and abruptly attached to the fungal comb, with few or no visible hyphal development in the early stage. At later stages, hyphae emerged from the sclerotial crust, growing upwards with minimal branching until the sclerotia became dry and hollow. *X. minima* strains grew readily on PDA and WA plates (Suppl. material 2: fig. S10). As in a recent study, this sclerotium-forming *Xylaria minima* was also observed in *Macrotermes annandalei* and *M. gilvus* (Yang et al. 2026).

Meanwhile, nine paired-alates rearing boxes successfully produced eggs which hatched into the first generation of termites under laboratory conditions. With continuous provision of sterilised food, one colony developed initial comb-like structures (Suppl. material 2: fig. S11). However, after the termites were removed and the comb-like structures were incubated in the laboratory for one week, no *Xylaria* mycelial growth was observed (Suppl. material 2: fig. S11).

### Insecticidal activity of *Metarhizium* sp. Cmu-b60 against fungus-growing termites

**Figure 6.**
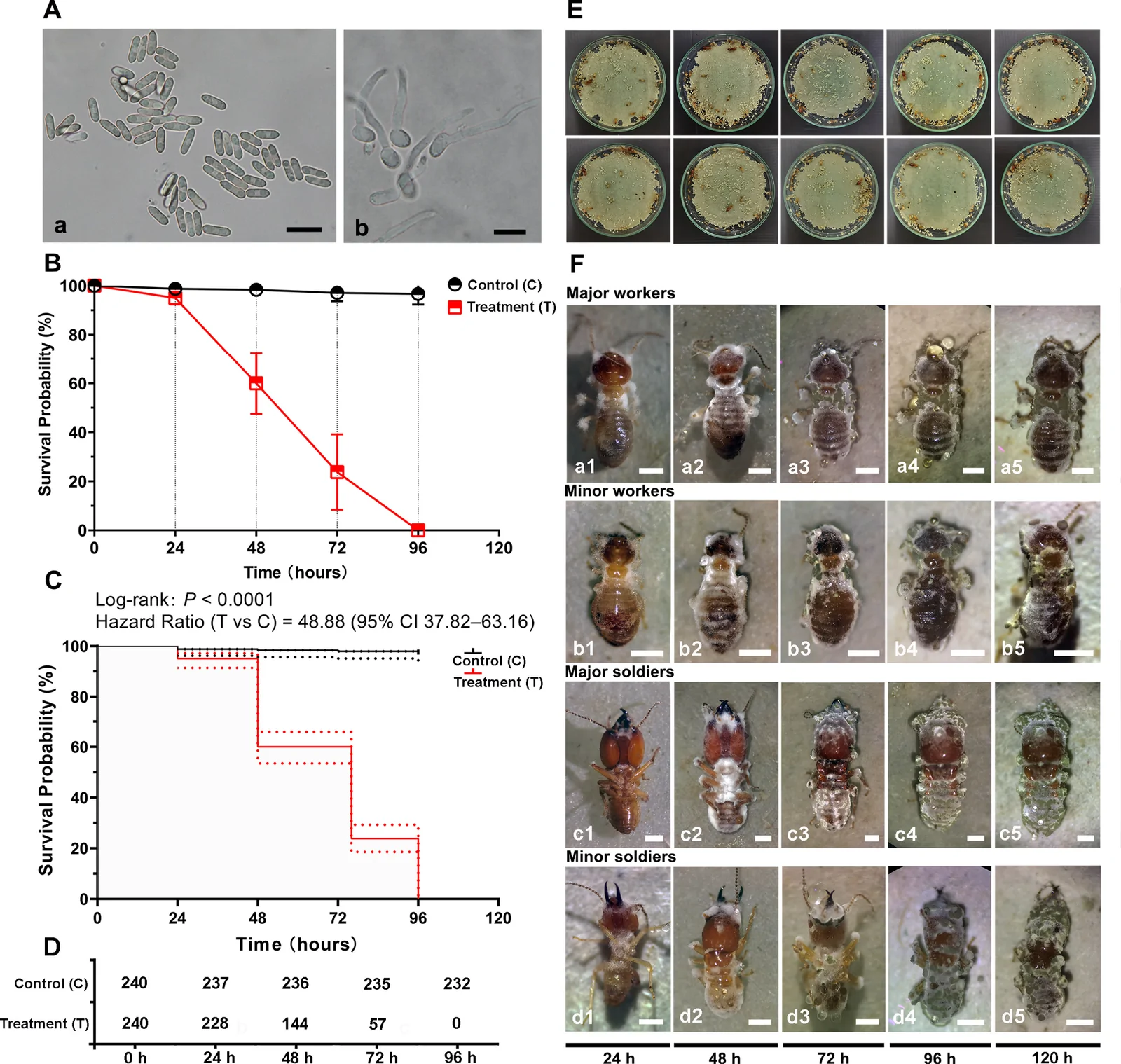
*Metarhizium* infection, termite mortality and morphological symptoms observed during pathogenesis. **A** Conidia (a) and germinated conidia (b) of *Metarhizium* sp. Cmu-b60 after 12 h of growth on PDA, scale bar = 10 μm; **B** Survival rate of termites treated with *Metarhizium* (T) compared with the controls (C) 96 h post-inoculation. Data are shown as mean ± SD (n = 10); **C** Kaplan-Meier survival curves for control (C) and infected (T) groups, including log-rank test results and hazard ratio (HR) with 95% CI; **D** Number of surviving individuals recorded at each time point (0–96 h) for control group (C) and treatment group (T); **E** Negative control plates, in which termites were treated only with distilled water containing 0.02% Tween 80, were opened and examined at 96 hours; **F** Progressive external growth of *Metarhizium* infection in major workers, minor workers, major soldiers and minor soldiers was recorded at 24, 48, 72, 96 and 120-h intervals. Scale bar: 1 mm.

When grown on PDA, the Cmu-b60 isolate formed yellow to dark green conidial bundles upon maturation. The conidia were unicellular and clavate, measuring 6–8 × 2–3 μm (mean = 7 × 2.5 μm, n = 20), with blunt apices (Fig. 6A).

To evaluate the virulence of the Cmu-b60 isolate, a bioassay was conducted against its original host, *Macrotermes gilvus*, using a conidial suspension of 1 × 10^8^ conidia ml^-1^. The survival rate of the infected group (n = 10 replicates) was compared with that of the negative control group (n = 10 replicates) over a 96-hour period. The control group maintained a consistently high survival (> 96.67%) throughout the experiment. In contrast, the infected group exhibited a rapid decline in survival, beginning at 95% survival 24 hours post-inoculation and reaching 100% mortality by 96 hours. Fungal subculture, DNA extraction and ITS sequencing from corpses confirmed that the strain was identical to Cmu-b60. Statistical analysis confirmed the highly significant difference in mortality. Survival analysis using a log-rank test confirmed that the difference between the treatment and control groups was statistically significant (*χ*^2^ = 475.6, *df* = 1, *P* < 0.0001). The Hazard Ratio (HR) test indicated an approximately 49-fold increase in mortality in the infected (T) group compared with the control (C) group (HR = 48.88; 95% CI, 37.82–63.16) (Fig. 6; Suppl. material 1: tables S8, S9).

### Termite behavioural responses towards mycelial plugs of comb weeds (*Xylaria*) and termite entomopathogen (*Metarhizium*)

**Figure 7.**
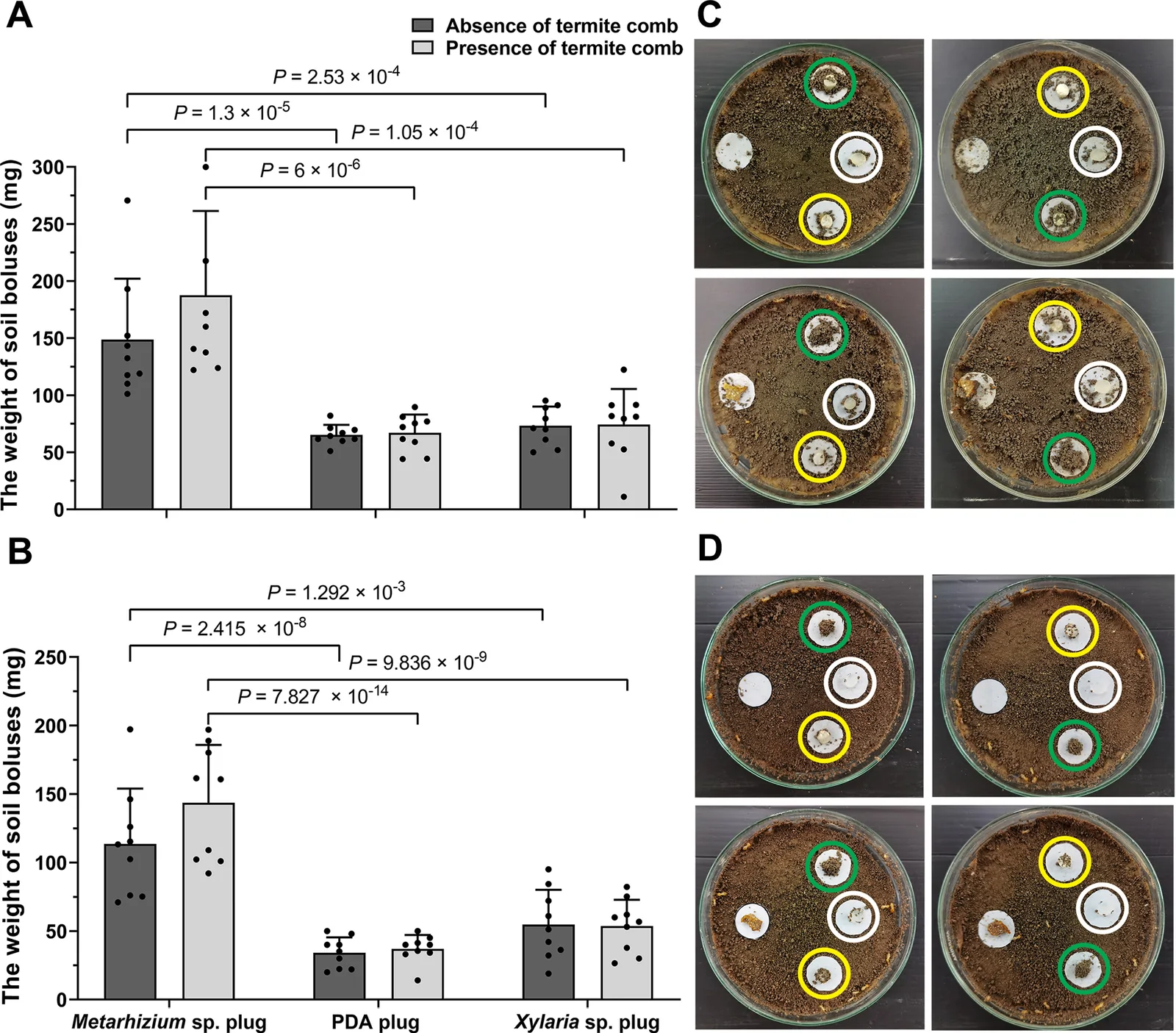
Behavioural responses of fungus-growing termites to the comb weeds *Xylaria* spp. (*Xylaria* clade 20 used in *Macrotermes gilvus*; *Xylaria* clade 11 used in *Odontotermes* sp.), the termite entomopathogen *Metarhizium* sp. Cmu-b60, and PDA (control). Left panels show the mean mass (± SD, n = 9) of soil boluses deposited with each fungus under conditions of presence or absence of termite comb for *Macrotermes gilvus***(A)** and *Odontotermes* sp. **(B)**, with post hoc comparison *p*-values indicated. Representative images illustrating termite behavioural responses to each treatment are presented for *Macrotermes gilvus***(C)** and *Odontotermes* sp. **(D)**. The green circle indicates the *Metarhizium* sp. plug, the yellow circle indicates the *Xylaria* plug and the white circle indicates the PDA plug (Control).

When fungus-growing termites were simultaneously exposed to the two potential threats — the comb weed *Xylaria* clades 11 and 20 and the entomopathogenic fungus *Metarhizium* sp. Cmu-b60 — along with a PDA control plug, their burying responses were markedly different (Fig. 7; Suppl. material 1: tables S10, S11). The presence or absence of the fungal comb had no significant effect on the weight of soil boluses deposited (GZLM: *Macrotermes gilvus*: *Wald χ^2^* = 1.280, df = 1, *P* = 0.258; *Odontotermes* sp.: *Wald χ^2^* = 1.185, df = 1, *P* = 0.276). In contrast, fungus identity exerted a strong effect on burying behaviour (*M. gilvus*: *Wald χ^2^* = 97.989, df = 2, *P* < 0.001; *Odontotermes* sp.: *Wald χ^2^* = 169.779, df = 2, *P* < 0.001), whereas the interaction between comb presence and fungus identity was not significant (*Macrotermes gilvus*: *Wald χ^2^* = 1.292, df = 2, *P* = 0.524; *Odontotermes* sp.: *Wald χ^2^* = 1.291, df = 2, *P* = 0.525). Regardless of the presence or absence of termite comb, exposure to *Metarhizium* elicited significantly more bolus deposition than either PDA or *Xylaria* in both *Macrotermes gilvus* and *Odontotermes* sp. (Bonferroni-corrected GLM, all *P* ≤ 0.001) (Suppl. material 1: table S12). In contrast, no significant difference was detected between *Xylaria* and PDA in either species (all *P* ≥ 0.215) (Suppl. material 1: table S12).

In descriptive comparisons, based on mean ± SD, *Macrotermes gilvus* deposited more soil boluses than *Odontotermes* sp. across all three treatments. The largest difference was observed under *Metarhizium* exposure (168.17 ± 65.57 vs. 130.28 ± 44.81), followed by PDA (66.24 ± 12.47 vs. 35.57 ± 10.51), while the difference was less pronounced under *Xylaria* (73.78 ± 24.29 vs. 54.24 ± 21.81) (Suppl. material 1: tables S10, S11).

The results obtained in this study showed that soil-bolus deposition effectively inhibited the growth of both *Xylaria* and *Metarhizium in vitro*. On WA control plates after one week, *Xylaria* sp. clade 20 produced upright, long, aerial mycelia or stromata, as well as immersed mycelia within the agar, whereas *Xylaria* sp. clade 11 and *Metarhizium* sp. Cmu-b60 produced only immersed mycelia (Suppl. material 2: fig. S12). In contrast, in WA plates treated with soil boluses, fungal growth was strongly suppressed, with only limited mycelial or stromatal growth emerging from the plugs of *Xylaria* sp. clade 11 (GI ± SD (%): 66.21% ± 9.32) and clade 20 (60.7% ± 7.9). However, although some plugs still produced conidia, the immersed mycelia were rarely observed. For *Metarhizium* plugs, growth was strongly inhibited regardless of whether the soil boluses were deposited by *Macrotermes gilvus* or *Odontotermes* sp. (GI ± SD: 68.32% ± 5.68 and 66.75% ± 6.34, respectively) and unidentified bacterial growth was occasionally observed around the plugs (Suppl. material 2: fig. S12). Despite these differences in fungal identity, the degree of radial growth inhibition by deposited soil boluses did not differ significantly between *Xylaria* sp. clade 11 and clade 20 and *Metarhizium* sp. Cmu-b60 (one-way ANOVA: *F*_3_,_12_ = 0.796, *P* = 0.519).

### Repellent effect of *Metarhizium* conidia-contaminated food on termites

The plates were examined after 120 hours (5 days). In the control groups, the termites remained highly active throughout the experimental period, with no mortality or signs of fungal infection and newly-hatched termites were observed. The continuous movement and feeding activity of the termites led to extensive dispersal of the provided food material, creating a visibly disordered plate environment (Suppl. material 2: fig. S13A). In contrast, termites exposed to *Metarhizium*-contaminated food exhibited 100% mortality and dead individuals were covered by dense white mycelial growth indicative of successful fungal infection; subculture, DNA extraction and ITS sequencing confirmed that it was the same strain as Cmu-b60. Termites showed no intensive movement or feeding activity, as evidenced by the undisturbed, neatly accumulated food at the corners of the plates. These results indicate that first-generation termites from the lab innately avoid *Metarhizium* conidia-contaminated food, and that this avoidance is accompanied by a rapid, lethal pathogenic effect of *Metarhizium* under laboratory conditions, occurring even in the absence of direct physical contact between newly-introduced termites and the fungal inoculum (Suppl. material 2: fig. S13B).

### *In vitro* inhibition of comb weeds (*Xylaria*) and termite entomopathogen (*Metarhizium*) by termite-associated bacterial isolates

**Figure 8.**
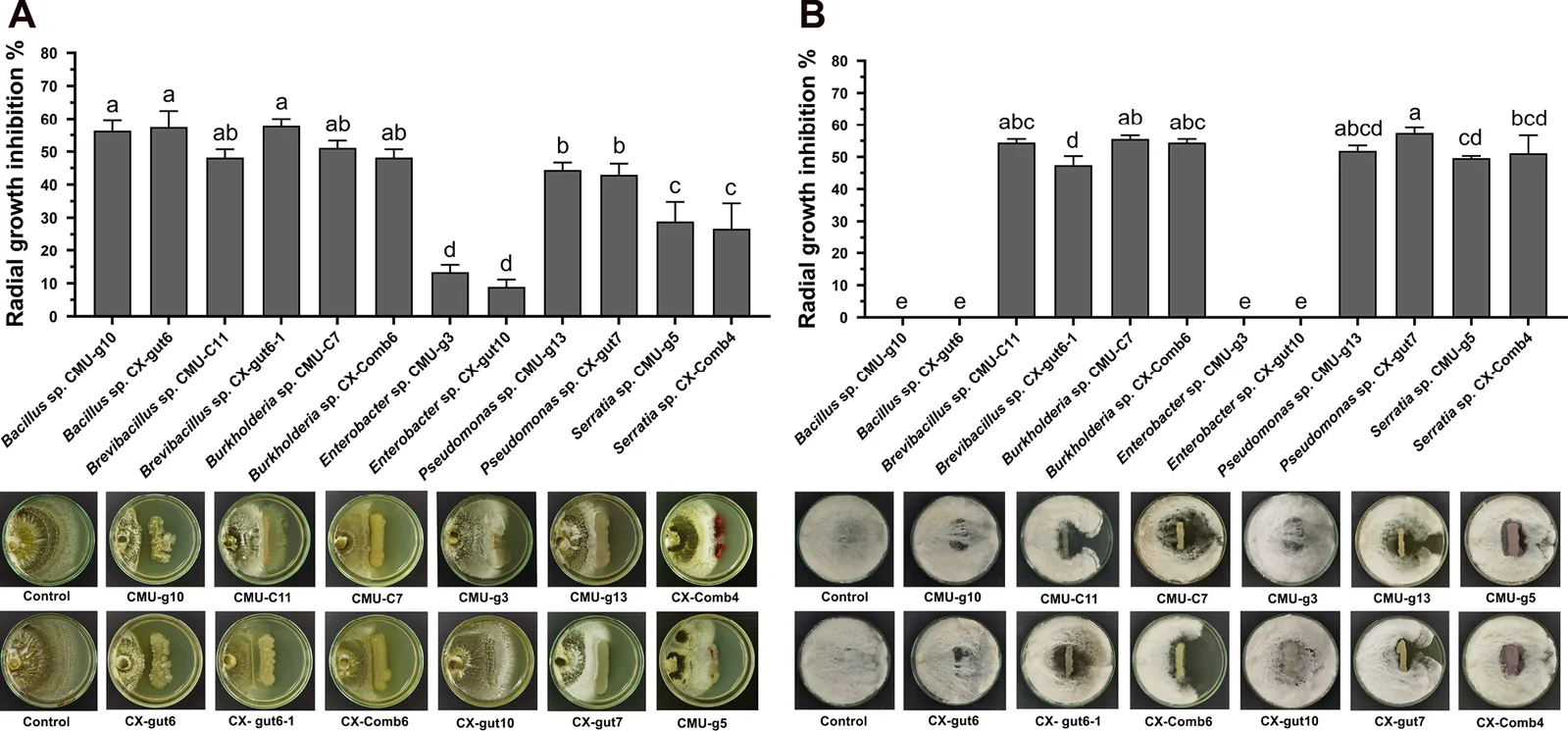
*In vitro* inhibition of *Metarhizium***(A)** and *Xylaria***(B)** by 12 bacterial isolates from termite colonies after 5 d (bacterium-*Xylaria* co-cultures) and for 15 d (bacterium-*Metarhizium* co-cultures). Representative images are shown in the panels below. Bars sharing the same lowercase letter are not significantly different according to Tukey’s HSD post hoc test (*p* > 0.05).

All 12 tested bacterial isolates (Suppl. material 1: tables S1, S2) exhibited varying degrees of growth inhibition against the termite entomopathogen *Metarhizium* sp. Cmu-b60. Four strains of the genera *Bacillus*, *Brevibacillus* and *Burkholderia* suppressed fungal growth by more than 50%. Interactions with *Bacillus* were characterised by pronounced inhibition zones, indicating strong antagonistic activity (Fig. 8; Suppl. material 1: table S13). In contrast, the same bacterial isolates displayed either weak or no inhibitory effects against the comb weed *Xylaria* sp. clade 20. Only six isolates from the genera *Brevibacillus*, *Burkholderia*, *Pseudomonas* and *Serratia* showed growth inhibition exceeding 50%. *Xylaria* rapidly produced abundant aerial mycelia, sometimes growing above colonies of *Bacillus* and *Enterobacter* isolates. In contrast, interactions with *Brevibacillus*, *Burkholderia*, *Pseudomonas* and *Serratia* resulted in a clear concave mycelial retreat, consistent with active suppression of fungal growth (Fig. 8; Suppl. material 1: table S13).

## Discussion

Using a conventional dilution-plating approach, we recovered 600 fungal isolates representing 79 genera from the combs and guts of six colonies across two termite genera. This culture-dependent method inevitably captured only a fraction of the diversity, about 20% of the 418 genera detected by nanopore sequencing (Agarwal et al. 2024). The isolates obtained showed considerable taxonomic overlap with those previously identified via culture-independent and culture-dependent sequencing from fungus combs (fresh and decaying), alates, workers, nymphs and termite-generated defensive soil boluses (Sibanda et al. 2019; Nel et al. 2021; Agarwal et al. 2024; Panchal et al. 2025; Yang et al. 2025). For bacteria, we identified several members of the genera *Bacillus*, *Burkholderia* and *Pseudomonas*, which have been isolated and suggested to function as fungistatic microbes contributing to colony defence (Murphy et al. 2021; Agarwal et al. 2024; Panchal et al. 2025; Yang et al. 2025). Previously using 454-pyrosequencing, Otani et al. (2014) detected 26 bacterial phyla across five genera (nine species) of fungus-growing termites. In contrast, our culture-dependent approach recovered only 59 isolates across three phyla, indicating that a large proportion of comb- and gut-associated bacteria are unculturable using the dilution-plating method. Therefore, more effective methods for isolating fungi and bacteria from termite guts and combs are needed to improve culture recovery (Ahmad et al. 2025). Due to the confounding effect of termite genus and geographic location, we did not perform cross-colony statistical comparisons. Instead, we conducted paired comparisons of the comb and gut fungal communities within each termite genus. In contrast to previous studies showing that termite guts harbour higher bacterial diversity than combs (Long et al. 2010; Makonde et al. 2015; Otani et al. 2016), our findings suggest that, within the same fungus-growing termite species at the same location, the comb- and gut-associated fungal communities are not significantly different (Fig. 4). However, it should be emphasised that these results are culture-dependent only; for bacteria, the diversity of culturable strains is clearly insufficient to capture the full community. Therefore, future studies employing culture-independent approaches are needed to substantiate these results.

Our study demonstrated that highly virulent entomopathogens, such as *Metarhizium*, can be recovered from healthy termite combs using dilution plating. Conversely, the recovery of comb-specialised *Xylaria* still relies primarily on the incubation of comb fragments. Previous studies suggested that, due to the termite hygienic behaviour, *Xylaria* is typically not distributed throughout the comb as a continuous mycelial network, but rather persists either as spores or as small localised mycelial patches (Batra and Batra 1979; Visser et al. 2009). Adaptive evolution of *Xylaria* to fungus comb is probably the reason why no strain belonging to the comb-specialised *Xylaria* was isolated by the PDA dilution-plating method. This spatial distribution may explain why incubating large intact fungus combs increases the probability of eventual dominance by the most competitive *Xylaria* species/strains. Consequently, to enhance the recovery and diversity of *Xylaria*, we recommend subdividing the fungus comb into small (gram-scale) units, which may mitigate competitive exclusion amongst strains and, thereby, facilitate the isolation of less dominant taxa. In addition, most *Xylaria* isolates failed to sporulate on PDA media; thus, long-term cultivation on alternative media, such as Difco oatmeal agar (OMA), may be required to induce sporulation and enable formal species descriptions (Ju et al. 2023). Moreover, some putative new clades of *Xylaria* lacked complete multi-locus data; thus, additional gene sequences will be required to enhance taxonomic resolution (Suppl. material 1: table S7). Our results are consistent with earlier findings that termite-associated *Xylaria* species specifically colonise fungus combs and that individual colonies harbour multiple *Xylaria* species (Li et al. 2025). Although we observed high cryptic diversity and low clade overlap amongst termite genera and across geographic locations, our study was unable to determine whether *Xylaria* clade composition is primarily driven by host genus or geographic origin. Accordingly, future investigations should systematically compare *Xylaria* communities associated with the same termite genus across disparate geographic regions, as well as those associated with different termite genera within the same region, to elucidate the relative contributions of host specificity and geographic isolation to community assembly.

Previous studies have suggested that termites act as dispersal agents for *Xylaria*, probably because species with smaller spores can be easily ingested or otherwise transported by termites (Rogers 2000; Rogers et al. 2005). In the current study, laboratory-established incipient colonies showed no emergence of *Xylaria* mycelia in termite-free controls, suggesting that *Xylaria* is unlikely to be vertically transmitted by alates; alternatively, its emergence may have been suppressed under laboratory conditions (Suppl. material 2: fig. S11). Therefore, it is necessary to explore whether the laboratory’s incipient nests harbour comb-specialised *Xylaria* using culture-independent sequencing. Meanwhile, Visser et al. (2009) also suggested that these propagules or spores of *Xylaria* are likely introduced into the nest through termites’ foraging activities rather than through soil contact. Unlike free-living *Xylaria*, termite-associated *Xylaria* are adapted to comb substrates and are rarely found in non-comb environments, a pattern suggested to result from a significant reduction in genes encoding cellulose-degrading enzymes, which may preclude a free-living lifestyle (Fricke et al. 2023). However, why the termite-associated clade of *Xylaria* is almost exclusively associated with termite fungus combs and how it is introduced into and persists within termite colonies remain unclear.

In examining termite behavioural responses to mycelial plugs of *Xylaria* (a common comb weed) and *Metarhizium* (an entomopathogenic fungus), we introduced methodological refinements to our earlier work, including replacing agar media with autoclaved soil, adding a PDA plug as a negative control and expanding replication across two genera (Yang et al. 2025). As expected, *Metarhizium* plugs elicited significant burial activity from termites, whereas *Xylaria* plugs received significantly lower levels of attention comparable to the PDA control; this response profile remained consistent regardless of the presence or absence of comb fragments bearing fungus nodules (Fig. 7). However, the underlying reasons for this differential behavioural response remain unclear. Descriptive comparisons revealed that *Macrotermes gilvus* deposited more soil boluses than *Odontotermes* sp. across all three treatments (*Metarhizium*, *Xylaria* and PDA). These patterns suggest that *M. gilvus* tends to exhibit a stronger burying response than *Odontotermes* sp., which may be related to its larger body size and may reflect a stronger hygienic capability.

*In vitro* assays further demonstrated that mycelial plugs of *Metarhizium* and *Xylaria* were effectively suppressed when covered with soil boluses. The deposition of soil boluses may create local hypoxia (Katariya et al. 2018), thereby aiding fungal suppression (Panchal et al. 2025). Additional dual-culture assays showed that comb- and gut-associated isolates of *Bacillus*, *Brevibacillus*, *Burkholderia* and *Pseudomonas* also inhibited the growth of *Metarhizium* and *Xylaria*. Based on clear zones and inhibition rates, these bacteria seem to exhibit stronger inhibitory effects against *Metarhizium* than against the comb-associated *Xylaria*. Here, we hypothesise that this lower inhibition may be attributed to two non-mutually exclusive factors: (i) *Xylaria* rapidly produces abundant aerial mycelia that occasionally grow above the bacterial colonies and (ii) *Xylaria* may have evolved tolerance to bacterial antifungal metabolites as a result of its long-term latent persistence within healthy termite fungus combs. However, further research is necessary to confirm this. The specific compounds responsible for the antifungal activities of these bacteria and soil boluses remain unidentified. However, they may originate from termite defensive salivary secretions, which contain antimicrobial peptides, such as spinigerin (Lamberty et al. 2001) and termicin (Da Silva et al. 2003; Bulmer and Crozier 2004), as well as β-1,3-glucanases with known antifungal properties (Hamilton et al. 2011; Martin and Bulmer 2018). In addition, various bacteria associated with fungus-growing termites have been shown to produce fungicidal metabolites, including natalamycin A and the microtermolides A and B, which are produced by *Streptomyces* spp. (Carr et al. 2012; Kim et al. 2014), bacillaene A from *Bacillus* spp. (Um et al. 2013), macrotermycins A and C from *Amycolatopsis* sp. M39 (Beemelmanns et al. 2017) and banegasine from *Actinomadura* sp. (Benndorf et al. 2018). These bacteria and their antibiotics are likely involved in colony defence, potentially through enclosure within soil boluses (Panchal et al. 2025) and incorporation into comb materials (Nakashima and Matsuura 2025), thereby suppressing unwanted fungal invasions.

Our *in vitro* assays, combined with the observed fungal-burying behaviour, indicate that these fungistatic bacteria and termite defensive behaviour serve a dual function: they not only help maintain *Termitomyces* monoculture by suppressing comb weeds such as *Xylaria* spp., but also effectively protect termites against entomopathogenic fungi such as *Metarhizium*.

## Additional information

### Artificial Intelligence (AI) use

The authors accept full responsibility for the content of the manuscript, including the disclosure of any use of AI.

No AI tools were used in the preparation of this manuscript.

### Adherence to national and international regulations

All the fungal strains used in this study have been legally obtained, respecting the Convention on Biological Diversity (Rio Convention).

### Author ORCIDs

E.-F. Yang https://orcid.org/0000-0003-2385-6402

S.C. Karunarathna https://orcid.org/0000-0001-7080-0781

M.C. Samarakoon https://orcid.org/0000-0002-4815-125X

I. Promputtha https://orcid.org/0000-0003-3376-4376

Q.-Q. Tao https://orcid.org/0009-0002-8429-2444

C.-I. Chiu https://orcid.org/0000-0002-1603-0736

Z.-X. Zhao https://orcid.org/0000-0003-4428-8547

X.-J. Luo https://orcid.org/0009-0001-4879-6816

M.M. Gakuubi https://orcid.org/0000-0003-3154-2614

S.L. Stephenson https://orcid.org/0000-0002-9207-8037

A.M. Elgorban https://orcid.org/0000-0003-3664-7853

A.T. Alfagham https://orcid.org/0000-0001-9008-3679

N. Suwannarach https://orcid.org/0000-0002-2653-1913

E. Tarafder https://orcid.org/0000-0002-3680-3433

J.-Y. Yang https://orcid.org/0009-0000-0375-990X

F.-Q. Yu https://orcid.org/0000-0001-7931-080X

D.-Q. Dai https://orcid.org/0000-0001-8935-8807

M.-M. Wang https://orcid.org/0000-0003-3747-5432

S. Tibpromma https://orcid.org/0000-0002-4706-6547

### Data availability

All of the data that support the findings of this study are available in the main text or Supplementary Information.

## Supplementary materials

Supplementary material 1 Additional tables S1–S13 Data type docx https://binary.pensoft.net/file/1795621This dataset is made available under the Open Database License (http://opendatacommons.org/licenses/odbl/1.0/). The Open Database License (ODbL) is a license agreement intended to allow users to freely share, modify, and use this Dataset while maintaining this same freedom for others, provided that the original source and author(s) are credited. Er-Fu Yang, Samantha C. Karunarathna, Milan C. Samarakoon, Itthayakorn Promputtha, Qian-Qian Tao, Chun-I Chiu, Zhen-Xiong Zhao, Xiu-Juan Luo, Martin M. Gakuubi, Steven L. Stephenson, Abdallah M. Elgorban, Alanoud T. Alfagham, Nakarin Suwannarach, Entaj Tarafder, Jing-Ya Yang, Fu-Qiang Yu, Dong-Qin Dai, Mei-Mei Wang, Saowaluck Tibpromma

Supplementary material 2 Additional figures S1–S13 Data type docx https://binary.pensoft.net/file/1795622This dataset is made available under the Open Database License (http://opendatacommons.org/licenses/odbl/1.0/). The Open Database License (ODbL) is a license agreement intended to allow users to freely share, modify, and use this Dataset while maintaining this same freedom for others, provided that the original source and author(s) are credited. Er-Fu Yang, Samantha C. Karunarathna, Milan C. Samarakoon, Itthayakorn Promputtha, Qian-Qian Tao, Chun-I Chiu, Zhen-Xiong Zhao, Xiu-Juan Luo, Martin M. Gakuubi, Steven L. Stephenson, Abdallah M. Elgorban, Alanoud T. Alfagham, Nakarin Suwannarach, Entaj Tarafder, Jing-Ya Yang, Fu-Qiang Yu, Dong-Qin Dai, Mei-Mei Wang, Saowaluck Tibpromma
