# Supplementary material for: Termite sanitary behaviour and their associated bacteria protect against latent Xylaria and entomopathogenic Metarhizium
Source: IMA Fungus. 2026 Oct 1;17:e211888. doi: 10.3897/imafungus.17.211888 (PMC13650603; doi:10.3897/imafungus.17.211888)
Supplement: Supplementary file 1 [file imafungus-17-e211888-s001.docx]

**Supplementary Figures**

**Article Title:**

Termite sanitary behaviour and their associated bacteria protect against latent *Xylaria* and entomopathogenic *Metarhizium*

**Authors:**
Er-Fu Yang, Samantha C. Karunarathna, Milan C. Samarakoon, Itthayakorn Promputtha, Qian-Qian Tao, Chun-I Chiu, Zhen-Xiong Zhao, Xiu-Juan Luo, Martin M. Gakuubi, Steven L. Stephenson, Abdallah M. Elgorban, Alanoud T. Alfagham, Nakarin Suwannarach, Entaj Tarafder, Jing-Ya Yang, Fu-Qiang Yu, Dong-Qin Dai, Mei-Mei Wang, Saowaluck Tibpromma

**Corresponding Authors:**
Mei-Mei Wang — wangmeimei7163@163.com
Saowaluck Tibpromma — saowaluckfai@gmail.com

**Fig. S1** The 59 bacterial strains isolated from fungus-farming termite systems, shown here cultured on NA. (**A**) Bacterial strains isolated from the combs and guts of *Odontotermes* sp. from Chuxiong, Yunnan. (**B**) Bacterial strains isolated from combs and guts of *Macrotermes gilvus* from Chiang Mai, Thailand.

**Fig. S2** Two hundred fungal strains isolated from the inner portions of combs in three *Macrotermes gilvus* colonies (n = 3) in Chiang Mai, Thailand, shown here cultured on PDA.

**Fig. S3** Two hundred fungal strains isolated from the portions of combs of three *Odontotermes* colonies (n = 3) in Chuxiong, China, shown here cultured on PDA.

**Fig. S4** One hundred fungal strains isolated from the guts of *Macrotermes gilvus* workers (n = 3 colonies) from Chiang Mai, Thailand, shown here cultured on PDA.

**Fig. S5** One hundred fungal strains isolated from the guts of *Odontotermes* workers (n = 3 colonies) from Chuxiong, China, shown here cultured on PDA.

**Fig. S6** Relative abundance of fungal isolates at the phylum and genus levels from combs and guts of *Macrotermes gilvus* (Thailand) and *Odontotermes* sp. (China). Each column represents the pooled relative abundance of three biological replicate colonies (n = 3 per termite genus/site).

**Fig. S7** Alpha-diversity indices (Chao1 and Shannon) of fungal communities from combs and guts of *Macrotermes gilvus* (**a**) and *Odontotermes* sp. (**b**).

**Fig. S8** Sixty-two Xylaria isolates obtained from inner comb fragments of three *Macrotermes gilvus* colonies (Thailand) after one month of incubation, cultured on PDA (Colony 1: CMU-A; Colony 2: CMU-a; Colony 3: CMU-X)

**Fig. S9** Fifty *Xylaria* isolates obtained from comb fragments of two *Odontotermes* sp. colonies (China), after 10 days of incubation, cultured on PDA. (Colony 1: CX with numbers; Colony 2: CX with letters)

**Fig. S10** Illustration of a sclerotium-forming, comb-specialised *Xylaria* species. (**a**–**j**) Sclerotia of *X. minima* forming on incubated two-gram fungus combs after one month in darkness at 27°C. (**k**,**l**) Compound- and stereomicroscopic observations of sclerotial tissues in vertical section. Scale bar f–j, l= 5 mm; k = 100 μm.

**Fig. S11 Establishment of new termite colonies in the laboratory and observations of initial comb formation.** (**a**) Paired alates placed in agar-filled boxes. (**b**,**c**) Food collected from natural colonies. (**d**) Boxes containing the king and queen, along with hatched eggs and food materials, kept in dark. (**e**,**f**) After three months, the first-generation termites matured, and initial comb-like structures were occasionally observed. (**g**) Initial comb piled by termite feces. No Xylaria mycelia were observed in the newly established colonies under laboratory conditions, nor in the boxes from which termites were removed after three months.

**Fig. S12** Effect of soil bolus deposition on the radial growth of fungal plugs on water agar. (**A**) Bar plot showing the radial growth of *Metarhizium* sp. and *Xylaria* spp. inhibited by soil bolus deposition. (**B**–**E**) Images of the corresponding water agar plates.

**Fig. S13 (A) Termites remained active, leading to scattered food material and no mortality at 0 and 120 hours post-setup (hps). (B)** Food material contaminated with *Metarhizium conidia* (1ml of 1 × 10⁸ conidia/ml) resulted in 100% mortality by 120 hps, and food piles remained undisturbed, indicating avoidance behaviour.
