# Supplementary material for: Termite sanitary behaviour and their associated bacteria protect against latent Xylaria and entomopathogenic Metarhizium
Source: IMA Fungus. 2026 Oct 1;17:e211888. doi: 10.3897/imafungus.17.211888 (PMC13650603; doi:10.3897/imafungus.17.211888)
Supplement: Supplementary file 2 [file imafungus-17-e211888-s002.docx]

**Supplementary Tables**

**Article Title:**

Termite sanitary behaviour and their associated bacteria protect against latent *Xylaria* and entomopathogenic *Metarhizium*

**Authors:**
Er-Fu Yang, Samantha C. Karunarathna, Milan C. Samarakoon, Itthayakorn Promputtha, Qian-Qian Tao, Chun-I Chiu, Zhen-Xiong Zhao, Xiu-Juan Luo, Martin M. Gakuubi, Steven L. Stephenson, Abdallah M. Elgorban, Alanoud T. Alfagham, Nakarin Suwannarach, Entaj Tarafder, Jing-Ya Yang, Fu-Qiang Yu, Dong-Qin Dai, Mei-Mei Wang, Saowaluck Tibpromma

**Corresponding Authors:**
Mei-Mei Wang — wangmeimei7163@163.com
Saowaluck Tibpromma — saowaluckfai@gmail.com

**Table S1.** BLASTn identification of bacterial strains isolated from the guts and fungus combs of *Odontotermes* sp. in Chuxiong, Yunnan, China (Colonies 1–3).

| **Source** | **Isolate** | **Collection No.** | **Accession No.** | **BLASTn results (16S rRNA)** | **Similarity** | **Family** | **Order** |
| --- | --- | --- | --- | --- | --- | --- | --- |
| **Colony 1**-fungus combs | **CX-Comb1** | GMBCC1537 | PV652648 | *Bacillus subtilis* strain HZD-6 | 100.00% | Bacillaceae | Bacillales |
|  | **CX-Comb2** | GMBCC1538 | PV652649 | *Bacillus cereus* strain V6718 | 100.00% | Bacillaceae | Bacillales |
|  | **CX-Comb3** | GMBCC1539 | PV652650 | *Priestia megaterium* strain CS36 | 99.93% | Bacillaceae | Bacillales |
|  | **CX-Comb4** | GMBCC1540 | PV652651 | *Serratia marcescens* strain ZCL-01 | 99.57% | Yersiniaceae | Enterobacterales |
|  | **CX-Comb5** | GMBCC1541 | PV652652 | *Pseudomonas* sp. App30 | 99.78% | Pseudomonadaceae | Pseudomonadales |
| **Colony 2**-fungus combs | **CX-Comb6** | GMBCC1542 | PV652653 | *Burkholderia* sp. NLJ2 | 100.00% | Burkholderiaceae | Burkholderiales |
|  | **CX-Comb7** | GMBCC1543 | PV652654 | *Pseudomonas* sp. strain JLS70 | 99.93% | Pseudomonadaceae | Pseudomonadales |
|  | **CX-Comb8** | GMBCC1544 | PV652655 | *Pseudomonas koreensis* strain XT630 | 100.00% | Pseudomonadaceae | Pseudomonadales |
|  | **CX-Comb9** | GMBCC1545 | PV652656 | *Priestia megaterium* strain Y74 | 99.89% | Bacillaceae | Bacillales |
| **Colony 3**-fungus combs | **CX-Comb10** | GMBCC1546 | PV652657 | *Paraburkholderia edwinii* strain Pe01 | 100.00% | Burkholderiaceae | Burkholderiales |
|  | **CX-Comb11** | GMBCC1547 | PV652658 | *Rhodococcus* sp. CC11H | 100.00% | Nocardiaceae | Mycobacteriales |
|  | **CX-Comb12** | GMBCC1548 | PV652659 | *Priestia megaterium* strain B-78-3 | 100.00% | Bacillaceae | Bacillales |
| **Colony 1**-termite guts | **CX-Gut1** | GMBCC1549 | PV652660 | *Priestia megaterium* strain B-78-3 | 100.00% | Bacillaceae | Bacillales |
|  | **CX-Gut2** | GMBCC1550 | PV652661 | *Bacillus velezensis* strain ID-A04 | 100.00% | Bacillaceae | Bacillales |
|  | **CX-Gut3** | GMBCC1551 | PV652662 | *Pseudomonas* sp. strain CMB_CA125 | 100.00% | Pseudomonadaceae | Pseudomonadales |
| **Colony 2**-termite guts | **CX-Gut4** | GMBCC1552 | PV652663 | *Pseudomonas* sp. AO-1 | 100.00% | Pseudomonadaceae | Pseudomonadales |
|  | **CX-Gut5** | GMBCC1553 | PV652664 | *Pseudomonas extremaustralis* strain Y39-6 | 100.00% | Pseudomonadaceae | Pseudomonadales |
|  | **CX-Gut6** | GMBCC1554 | PV652665 | *Bacillus subtilis* strain SRCM125727 | 100.00% | Bacillaceae | Bacillales |
|  | **CX-Gut6-1** | GMBCC1555 | PV652666 | *Brevibacillus* sp. strain FJAT-10657 | 100.00% | Paenibacillaceae | Bacillales |
|  | **CX-Gut7** | GMBCC1556 | PV652667 | *Pseudomonas tolaasii* strain SC1V1-1 | 100.00% | Pseudomonadaceae | Pseudomonadales |
|  | **CX-Gut8** | GMBCC1557 | PV652668 | *Pseudomonas koreensis* strain MP131 | 100.00% | Pseudomonadaceae | Pseudomonadales |
|  | **CX-Gut9** | GMBCC1558 | PV652669 | *Paraburkholderia humisilvae* strain MAW7 | 99.26% | Burkholderiaceae | Burkholderiales |
|  | **CX-Gut10** | GMBCC1559 | PV652670 | *Enterobacter* sp. strain SMN3 | 99.93% | Enterobacteriaceae | **Enterobacterales** |
| **Colony 3**-termite guts | **CX-Gut11** | GMBCC1560 | PV652671 | *Bacillus* sp. strain CW15-3 | 100.00% | Bacillaceae | Bacillales |
|  | **CX-Gut12** | GMBCC1561 | PV652672 | *Pandoraea* sp. G3307 | 99.78% | Burkholderiaceae | **Burkholderiales** |
|  | **CX-Gut13** | GMBCC1562 | PV652673 | *Pseudomonas veronii* strain OST1911 | 100.00% | Pseudomonadaceae | Pseudomonadales |
|  | **CX-Gut14** | GMBCC1563 | PV652674 | *Pseudomonas* sp. AO-1 | 100.00% | Pseudomonadaceae | Pseudomonadales |
|  | **CX-Gut15** | GMBCC1564 | PV652675 | *Pseudomonas fluorescens* strain WT326 | 100.00% | Pseudomonadaceae | Pseudomonadales |

**Table S2.** BLASTn identification of bacterial strains isolated from the guts and fungus combs of *Macrotermes gilvus* in Chuxiong, Yunnan, China (Colonies 1–3).

| **Source** | **Isolates** | **Accession No.** | **BLASTn results (16S rRNA)** | **Similarity** | **Family** | **Order** |
| --- | --- | --- | --- | --- | --- | --- |
| **Colony 1**-fungus comb | **CMU-C1** | PX396356 | *Kosakonia* sp. SMBL-WEM22 | 99.72% | Enterobacteriaceae | Enterobacterales |
|  | **CMU-C2** | PX396357 | *Serratia marcescens* strain I2 | 100% | Yersiniaceae | **Enterobacterales** |
|  | **CMU-C3** | PX396358 | *Serratia nematodiphila* strain AAUB146b | 100% | Yersiniaceae | **Enterobacterales** |
|  | **CMU-C4** | PX396359 | *Bacillus velezensis* strain JS25R | 100% | Bacillaceae | Bacillales |
|  | **CMU-C5** | PX396360 | *Bacillus acidiceler* strain JN91 | 99.65% | Bacillaceae | Bacillales |
|  | **CMU-C6** | PX396361 | *Pseudomonas* sp. strain HSTU-Sh9 | 99.93% | Pseudomonadaceae | Pseudomonadales |
|  | **CMU-C7** | PX396362 | *Burkholderia gladioli* strain CACua-73 | 99.93% | Burkholderiaceae | Burkholderiales |
| **Colony 2**-fungus comb | **CMU-C8** | PX396363 | *Bacillus altitudinis* strain kv1 | 99.93% | Bacillaceae | Bacillales |
|  | **CMU-C9** | PX396364 | *Bacillus siamensis* strain PpSM8 | 99.72% | Bacillaceae | Bacillales |
|  | **CMU-C10** | PX396365 | *Pseudomonas* sp. strain M2 | 99.93% | Pseudomonadaceae | Pseudomonadales |
|  | **CMU-C11** | PX396366 | *Brevibacillus brevis* strain Azhar5 | 100% | Paenibacillaceae | Bacillales |
|  | **CMU-C12** | PX396367 | *Enterobacter cloacae* strain 1211LB-2 | 99.86% | Enterobacteriaceae | Enterobacterales |
| **Colony 3**-fungus comb | **CMU-C13** | PX396368 | *Enterobacter lignolyticus* SCF1 | 99.08% | Enterobacteriaceae | Enterobacterales |
|  | **CMU-C14** | PX396369 | *Enterobacter sacchari* strain HX148S | 99.86% | Enterobacteriaceae | Enterobacterales |
|  | **CMU-C15** | PX396370 | *Burkholderia paludis* strain GAR-13 | 100% | Burkholderiaceae | Burkholderiales |
|  | **CMU-C16** | PX396371 | *Bacillus velezensis* strain LZLJ01 | 99.78% | Bacillaceae | Bacillales |
|  | **CMU-C17** | PX396372 | *Bacillus cereus* strain doupp-01 | 100% | Bacillaceae | Bacillales |
| **Colony 1**-termite guts | **CMU-g1** | PX396373 | *Bacillus* sp. LM1(2011) | 99.65% | Bacillaceae | Bacillales |
|  | **CMU-g2** | PX396374 | *Enterobacter ludwigii* strain SWDB21 | 100% | Enterobacteriaceae | Enterobacterales |
|  | **CMU-g3** | PX396375 | *Enterobacter* sp. R4-368 | 99.93% | Enterobacteriaceae | Enterobacterales |
|  | **CMU-g4** | PX396376 | *Serratia marcescens* subsp*. marcescens s*train XS 22-6 | 99.93% | Yersiniaceae | **Enterobacterales** |
| **Colony 2**-termite guts | **CMU-g5** | PX396377 | *Serratia marcescens* strain 4P1 | 99.93% | Yersiniaceae | **Enterobacterales** |
|  | **CMU-g6** | PX396378 | *Citrobacter amalonaticus* strain 2023CK-01304 | 99.79% | Enterobacteriaceae | Enterobacterales |
|  | **CMU-g7** | PX396379 | *Enterobacter* sp. strain MHSB17 | 99.72% | Enterobacteriaceae | Enterobacterales |
|  | **CMU-g8** | PX396380 | *Providencia stuartii* strain prov-sta1 | 99.88% | Morganellaceae | Enterobacterales |
|  | **CMU-g9** | PX396381 | *Kosakonia oryzendophytica* strain XS_137-4 | 100% | Enterobacteriaceae | Enterobacterales |
|  | **CMU-g10** | PX396382 | *Bacillus cereus* strain BC1-1034 | 100% | Bacillaceae | Bacillales |
| **Colony 3-**termite guts | **CMU-g11** | PX396383 | *Bacillus cereus* strain doupp-01 | 99.93% | Bacillaceae | Bacillales |
|  | **CMU-g12** | PX396384 | *Bacillus cereus* strain doupp-01 | 99.93% | Bacillaceae | Bacillales |
|  | **CMU-g13** | PX396385 | *Pseudomonas urethralis* strain Hy6-19 | 99.59% | Pseudomonadaceae | Pseudomonadales |
|  | **CMU-g14** | PX396386 | *Pantoea cypripedii* strain JR3406-1 | 99.71% | Erwiniaceae | Enterobacterales |

**Table S3.** BLASTn results of the 200 fungal strains isolated from the fungus combs of *Odontotermes* colonies (n = 3) in Chuxiong, Yunnan, China. (Colony 1: CB1–69; Colony 2: CU1–66; Colony 3: CY1–65)

| **Isolates** | **Collection No.** | **Accession No.** | **BLASTn results (ITS)** | **Similarity** | **Family** | **Order** |
| --- | --- | --- | --- | --- | --- | --- |
| **CX-CB1** | GMBCC1306 | PV644018 | *Linderina macrospora* strain BCRC31802 | 99.42% | Kickxellaceae | Kickxellales |
| **CX-CB2** | GMBCC1307 | PV644019 | *Trichoderma* sp. OTU035 AN-2016 | 100.00% | Hypocreaceae | Hypocreales |
| **CX-CB3** | GMBCC1308 | PV644020 | *Talaromyces albobiverticillius* strain GXM23016 | 99.82% | Aspergillaceae | Eurotiales |
| **CX-CB4** | GMBCC1309 | PV644021 | *Umbelopsis ramanniana* Um-113 | 99.34% | Umbelopsidaceae | Umbelopsidales |
| **CX-CB5** | GMBCC1310 | PV644022 | *Pestalotiopsis lushanensis* strain HF-080 | 99.65% | Pestalotiopsidaceae | Amphisphaeriales |
| **CX-CB6** | GMBCC1311 | PV644023 | *Pestalotiopsis clavata* FJ1-1 | 99.83% | Pestalotiopsidaceae | Amphisphaeriales |
| **CX-CB7** | GMBCC1312 | PV644024 | *Trichoderma koningiopsis* CTCCSJ-AYC50400 | 99.83% | Hypocreaceae | Hypocreales |
| **CX-CB8** | GMBCC1313 | PV644025 | *Cladosporium cladosporioides* FG29 | 100.00% | Cladosporiaceae | Cladosporiales |
| **CX-CB9** | GMBCC1314 | PV644026 | *Trichoderma harzianum*ZNCF17 | 99.83% | Hypocreaceae | Hypocreales |
| **CX-CB10** | GMBCC1315 | PV644027 | *Trichoderma harzianum* ZWPUEB10 | 100.00% | Hypocreaceae | Hypocreales |
| **CX-CB11** | GMBCC1316 | PV644028 | *Coniella quercicola* GUCC 190207.1 | 99.83% | Schizoparmaceae | Diaporthales |
| **CX-CB12** | GMBCC1317 | PV644029 | *Penicillium* sp. PSF15 | 99.63% | Aspergillaceae | Eurotiales |
| **CX-CB13** | GMBCC1318 | PV644030 | *Penicillium bialowiezense* CIRM-BRFM 3778 | 99.82% | Aspergillaceae | Eurotiales |
| **CX-CB14** | GMBCC1319 | PV644031 | *Preussia* sp. isolate 29 | 99.80% | Sporormiaceae | Pleosporales |
| **CX-CB15** | GMBCC1320 | PV644032 | *Umbelopsis* sp. isolate XXMF000011 | 98.25% | Umbelopsidaceae | Umbelopsidales |
| **CX-CB16** | GMBCC1321 | PV644033 | *Umbelopsis ramanniana* strain Um-113 | 99.64% | Umbelopsidaceae | Umbelopsidales |
| **CX-CB17** | GMBCC1322 | PV644034 | *Trichoderma harzianum* strain ZWPUEB10 | 100.00% | Hypocreaceae | Hypocreales |
| **CX-CB18** | GMBCC1323 | PV644035 | *Penicillium pancosmium* CIRM-BRFM 3759 | 100.00% | Aspergillaceae | Eurotiales |
| **CX-CB19** | GMBCC1324 | PV644036 | *Preussia* sp. isolate 29 | 99.61% | Sporormiaceae | Pleosporales |
| **CX-CB20** | GMBCC1325 | PV644037 | *Trichoderma* sp. Ppa1 | 100.00% | Hypocreaceae | Hypocreales |
| **CX-CB21** | GMBCC1326 | PV644038 | *Trametes versicolor* DCL31 | 99.83% | Polyporaceae | Polyporales |
| **CX-CB22** | GMBCC1327 | PV644039 | *Trichoderma* sp. BLE9 | 100.00% | Hypocreaceae | Hypocreales |
| **CX-CB23** | GMBCC1328 | PV644040 | *Umbelopsis* sp. SM13-27-38-2 | 100.00% | Umbelopsidaceae | Umbelopsidales |
| **CX-CB24** | GMBCC1329 | PV644041 | *Penicillium qii* strain AS3.15340 | 100.00% | Aspergillaceae | Eurotiales |
| **CX-CB25** | GMBCC1330 | PV644042 | *Mucor* sp. strain 2583-3 | 99.83% | Mucoraceae | Mucorales |
| **CX-CB26** | GMBCC1331 | PV644043 | *Scleroconidioma sphagnicola* UAMH 9731 | 100.00% | *Incertae sedis* | *Incertae sedis* |
| **CX-CB27** | GMBCC1332 | PV644044 | *Trichoderma* sp. isolate P0-020 | 99.83% | Hypocreaceae | Hypocreales |
| **CX-CB28** | GMBCC1333 | PV644045 | *Aspergillus* sp. isolate Parr 3-1 | 100.00% | Aspergillaceae | Eurotiales |
| **CX-CB29** | GMBCC1334 | PV644046 | *Trichoderma gamsii* S121RZ | 100.00% | Hypocreaceae | Hypocreales |
| **CX-CB30** | GMBCC1335 | PV644047 | *Mucor* sp. strain 2583-3 | 95.85% | Mucoraceae | Mucorales |
| **CX-CB31** | GMBCC1336 | PV644048 | *Trichoderma* sp. isolate Ppa1 | 100.00% | Hypocreaceae | Hypocreales |
| **CX-CB32** | GMBCC1337 | PV644049 | *Aspergillus* sp. isolate 1Out1 | 100.00% | Aspergillaceae | Eurotiales |
| **CX-CB33** | GMBCC1338 | PV644050 | *Mucor plumbeus* R97306 | 99.83% | Mucoraceae | Mucorales |
| **CX-CB34** | GMBCC1339 | PV644051 | *Trichoderma harzianum* IPBCC07_547 | 100.00% | Hypocreaceae | Hypocreales |
| **CX-CB35** | GMBCC1340 | PV644052 | X*ylaria grammica* isolate Otu0315 | 100.00% | Xylariaceae | Xylariales |
| **CX-CB36** | GMBCC1341 | PV644053 | *Aspergillus* sp. Parr 3-1 | 100.00% | Aspergillaceae | Eurotiales |
| **CX-CB37** | GMBCC1342 | PV644054 | *Mucor plumbeus* R97306 | 100.00% | Mucoraceae | Mucorales |
| **CX-CB38** | GMBCC1343 | PV644055 | *Umbelopsis isabellina* MPG-14a | 100.00% | Umbelopsidaceae | Umbelopsidales |
| **CX-CB39** | GMBCC1344 | PV644056 | *Trichoderma* sp. JSM 06261611 | 100.00% | Hypocreaceae | Hypocreales |
| **CX-CB40** | GMBCC1345 | PV644057 | *Trichoderma harzianum* isolate D15 | 99.83% | Hypocreaceae | Hypocreales |
| **CX-CB41** | GMBCC1346 | PV644058 | *Umbelopsis isabellina* SLF28 | 93.89% | Umbelopsidaceae | Umbelopsidales |
| **CX-CB42** | GMBCC1347 | PV644059 | *Mucor parviseptatus* CBS 417.77 | 100.00% | Mucoraceae | Mucorales |
| **CX-CB43** | GMBCC1348 | PV644060 | *Trichoderma viride* BBA 65450 | 100.00% | Hypocreaceae | Hypocreales |
| **CX-CB44** | GMBCC1349 | PV644061 | *Mucor* sp. strain 2583-3 | 100.00% | Mucoraceae | Mucorales |
| **CX-CB45** | GMBCC1350 | PV644062 | *Mucor plumbeus* isolate R97306 | 99.83% | Mucoraceae | Mucorales |
| **CX-CB46** | GMBCC1351 | PV644063 | *Mucor* sp. strain 2583-3 | 99.83% | Mucoraceae | Mucorales |
| **CX-CB47** | GMBCC1352 | PV644064 | *Mucor* sp. isolate M1-0153 | 99.83% | Mucoraceae | Mucorales |
| **CX-CB48** | GMBCC1353 | PV644065 | *Fusarium* sp. XL-D19 | 100.00% | Nectriaceae | Hypocreales |
| **CX-CB49** | GMBCC1354 | PV644066 | *Penicillium* sp. isolate 29AR21 | 99.64% | Aspergillaceae | Eurotiales |
| **CX-CB50** | GMBCC1355 | PV644067 | *Umbelopsis isabellina* isolate EF43 | 95.02% | Umbelopsidaceae | Umbelopsidales |
| **CX-CB51** | GMBCC1356 | PV644068 | *Trichoderma* sp. isolate P0-058 | 99.83% | Hypocreaceae | Hypocreales |
| **CX-CB52** | GMBCC1357 | PV644069 | *Trichoderma tomentosum* isolate EI 1000 | 99.66% | Hypocreaceae | Hypocreales |
| **CX-CB53** | GMBCC1358 | PV644070 | *Cladosporium cladosporioides* strain FG29 | 100.00% | Cladosporiaceae | Cladosporiales |
| **CX-CB54** | GMBCC1359 | PV644071 | *Trichoderma* sp. T004-R2-1 | 100.00% | Hypocreaceae | Hypocreales |
| **CX-CB55** | GMBCC1360 | PV644072 | *Preussia minima* strain EECC-653 | 99.80% | Sporormiaceae | Pleosporales |
| **CX-CB56** | GMBCC1361 | PV644073 | *Linnemannia* sp. XG12234-1-1 | 100.00% | Mortierellaceae | Mortierellales |
| **CX-CB57** | GMBCC1362 | PV644074 | *Scleroconidioma sphagnicola* JJ-18-24 | 99.82% | *Incertae sedis* | *Incertae sedis* |
| **CX-CB58** | GMBCC1363 | PV644075 | *Mucor variicolumellatus* isolate 14-W-1 | 99.83% | Mucoraceae | Mucorales |
| **CX-CB59** | GMBCC1364 | PV644076 | *Phialomyces arenicola* CBS:129398 | 99.82% | Aspergillaceae | Eurotiales |
| **CX-CB60** | GMBCC1365 | PV644077 | *Mortierella alpina* CBS 920.85 | 98.02% | Mortierellaceae | Mortierellales |
| **CX-CB61** | GMBCC1366 | PV644078 | *Penicillium* sp. GZU-BCECYN66-5 | 99.46% | Aspergillaceae | Eurotiales |
| **CX-CB62** | GMBCC1367 | PV644079 | *Gongronella* sp. strain SAUCC 4059-1 | 99.02% | Cunninghamellaceae | Mucorales |
| **CX-CB63** | GMBCC1368 | PV644080 | *Mucor* sp. isolate GMBCC1074 | 99.67% | Mucoraceae | Mucorales |
| **CX-CB64** | GMBCC1369 | PV644081 | *Trichoderma harzianum* strain ZWPUEB10 | 100.00% | Hypocreaceae | Hypocreales |
| **CX-CB65** | GMBCC1370 | PV644082 | *Trichoderma viridescens* CCTCC:AO400 | 100.00% | Hypocreaceae | Hypocreales |
| **CX-CB66** | GMBCC1371 | PV644083 | *Linnemannia* sp. strain XG12234-1-1 | 100.00% | Mortierellaceae | Mortierellales |
| **CX-CB67** | GMBCC1372 | PV644084 | *Trichoderma viridescens* CCTCC:AO400 | 100.00% | Hypocreaceae | Hypocreales |
| **CX-CB68** | GMBCC1373 | PV644085 | *Trichoderma gamsii* isolate DCR22 | 100.00% | Hypocreaceae | Hypocreales |
| **CX-CB69** | GMBCC1374 | PV644086 | *Absidia* sp. HZ-2021a strain CGMCC 3.16104 | 98.00% | Cunninghamellaceae | Mucorales |
| **CX-CU1** | GMBCC1437 | PV660844 | *Aspergillus flavus* CBS:126853 | 99.82% | Aspergillaceae | Eurotiales |
| **CX-CU2** | GMBCC1438 | PV660845 | *Humicola fuscoatra* strain MN-ZJ:8 | 99.82% | Chaetomiaceae | Sordariales |
| **CX-CU3** | GMBCC1439 | PV660846 | Trichoderma virens isolate TU | 100.00% | Hypocreaceae | Hypocreales |
| **CX-CU4** | GMBCC1440 | PV660847 | *Trichoderma virens* isolate TU | 100.00% | Hypocreaceae | Hypocreales |
| **CX-CU5** | GMBCC1441 | PV660848 | Lasiodiplodia theobromae isolate Lt-A2 | 100.00% | Botryosphaeriaceae | Botryosphaeriales |
| **CX-CU6** | GMBCC1442 | PV660849 | *Scedosporium dehoogii* isolate B11TUSc18 | 99.65% | Microascaceae | Microascales |
| **CX-CU7** | GMBCC1443 | PV660850 | *Aspergillus flavus* CBS:126853 | 99.64% | Aspergillaceae | Eurotiales |
| **CX-CU8** | GMBCC1444 | PV660851 | *Talaromyces dimorphus* strain GXM23012 | 100.00% | Aspergillaceae | Eurotiales |
| **CX-CU9** | GMBCC1445 | PV660852 | *Epicoccum* sp. isolate UgN65 | 99.80% | Didymellaceae | Pleosporales |
| **CX-CU10** | GMBCC1446 | PV660853 | *Scedosporium dehoogii* isolate B11TUSc18 | 99.65% | Microascaceae | Microascales |
| **CX-CU11** | GMBCC1447 | PV660854 | *Trichoderma* sp. isolate 769_E5 | 100.00% | Hypocreaceae | Hypocreales |
| **CX-CU12** | GMBCC1448 | PV660855 | Metarhizium lepidiotae BUM66.1 | 100.00% | Clavicipitaceae | Hypocreales |
| **CX-CU13** | GMBCC1449 | PV660856 | *Scedosporium apiospermum* B11TCHSc1 | 100.00% | Microascaceae | Microascales |
| **CX-CU14** | GMBCC1450 | PV660857 | Flavodon ambrosius isolate P15 | 100.00% | Irpicaceae | Polyporales |
| **CX-CU15** | GMBCC1451 | PV660858 | *Fusarium* sp. strain GFR30 | 100.00% | Nectriaceae | Hypocreales |
| **CX-CU16** | GMBCC1452 | PV660859 | Coprinellus radians IFM 66087 | 99.84% | Psathyrellaceae | Agaricales |
| **CX-CU17** | GMBCC1453 | PV660860 | *Humicola* sp. isolate GLBRC440 | 99.66% | Chaetomiaceae | Sordariales |
| **CX-CU18** | GMBCC1454 | PV660861 | *Lasiodiplodia theobromae* strain Eg36 | 99.82% | Botryosphaeriaceae | Botryosphaeriales |
| **CX-CU19** | GMBCC1455 | PV660862 | Bipolaris panici-miliacei ICMP 10262 | 100.00% | Pleosporaceae | Pleosporales |
| **CX-CU20** | GMBCC1456 | PV660863 | *Talaromyces* sp. strain BRIP 46763 | 100.00% | Aspergillaceae | Eurotiales |
| **CX-CU21** | GMBCC1457 | PV660864 | *Penicillium citrinum* clone SF_699 | 99.79% | Aspergillaceae | Eurotiales |
| **CX-CU22** | GMBCC1458 | PV660865 | Chaetomium sp. clone YB1605 | 99.63% | Chaetomiaceae | Sordariales |
| **CX-CU23** | GMBCC1459 | PV660866 | Scedosporium apiospermum B11CPSc26 | 100.00% | Microascaceae | Microascales |
| **CX-CU24** | GMBCC1460 | PV660867 | *Talaromyces dimorphus* strain GXM23012 | 100.00% | Aspergillaceae | Eurotiales |
| **CX-CU25** | GMBCC1461 | PV660868 | *Microascus gracilis* isolate ARM 1138 | 100.00% | Microascaceae | Microascales |
| **CX-CU26** | GMBCC1462 | PV660869 | *Gongronella* sp. isolate AB3 | 100.00% | Cunninghamellaceae | Mucorales |
| **CX-CU27** | GMBCC1463 | PV660870 | Purpureocillium lilacinum strain M2943 | 100.00% | Ophiocordycipitaceae | Hypocreales |
| **CX-CU28** | GMBCC1464 | PV660871 | Cladosporium cladosporioides MWL32 | 100.00% | Cladosporiaceae | Cladosporiales |
| **CX-CU29** | GMBCC1465 | PV660872 | Sporothrix narcissi TF35 | 100.00% | Ophiostomataceae | Ophiostomatales |
| **CX-CU30** | GMBCC1466 | PV660873 | *Aspergillus flavus* CBS:126853 | 99.00% | Aspergillaceae | Eurotiales |
| **CX-CU31** | GMBCC1467 | PV660874 | Nigrospora oryzae isolate UO-35 | 100.00% | *Incertae sedis* | *Incertae sedis* |
| **CX-CU32** | GMBCC1468 | PV660875 | Epicoccum sp. isolate UgN26 | 100.00% | Didymellaceae | Pleosporales |
| **CX-CU33** | GMBCC1469 | PV660876 | Penicillium sp. PSF39 | 100.00% | Aspergillaceae | Eurotiales |
| **CX-CU34** | GMBCC1470 | PV660877 | Talaromyces kendrickii isolate P1291 | 100.00% | Aspergillaceae | Eurotiales |
| **CX-CU35** | GMBCC1471 | PV660878 | Trichoderma harzianum CTCCSJ-ASD50452 | 100.00% | Hypocreaceae | Hypocreales |
| **CX-CU36** | GMBCC1472 | PV660879 | Trichoderma harzianum CTCCSJ-ASD50452 | 100.00% | Hypocreaceae | Hypocreales |
| **CX-CU37** | GMBCC1473 | PV660880 | Schizothecium glutinans isolate A620 | 100.00% | Lasiosphaeriaceae | Sordariales |
| **CX-CU38** | GMBCC1474 | PV660881 | Gongronella butleri CBS:228.36 | 98.81% | Cunninghamellaceae | Mucorales |
| **CX-CU39** | GMBCC1475 | PV660882 | Trichoderma viridescens CCTCC:AO400 | 100.00% | Hypocreaceae | Hypocreales |
| **CX-CU40** | GMBCC1476 | PV660883 | Trichoderma sp. isolate ST0-001 | 100.00% | Hypocreaceae | Hypocreales |
| **CX-CU41** | GMBCC1477 | PV660884 | Trichoderma koningii isolate S54 | 100.00% | Hypocreaceae | Hypocreales |
| **CX-CU42** | GMBCC1478 | PV660885 | Trichoderma pollinicola isolate QX211008-2 | 100.00% | Hypocreaceae | Hypocreales |
| **CX-CU43** | GMBCC1479 | PV660886 | *Penicillium citrinum* clone SF_699 | 100.00% | Aspergillaceae | Eurotiales |
| **CX-CU44** | GMBCC1480 | PV660887 | *Phialomyces arenicola* CBS:129398 | 100.00% | *Incertae sedis* | *Incertae sedis* |
| **CX-CU45** | GMBCC1481 | PV660888 | Mucor sp. isolate M1-0153 | 99.83% | Mucoraceae | Mucorales |
| **CX-CU46** | GMBCC1482 | PV660889 | Trichoderma sp. isolate 769_E5 | 100.00% | Hypocreaceae | Hypocreales |
| **CX-CU47** | GMBCC1483 | PV660890 | *Rhizopus delemar* UICC 26 | 100.00% | Rhizopodaceae | Mucorales |
| **CX-CU48** | GMBCC1484 | PV660891 | Trichoderma sp. isolate 769_E5 | 100.00% | Hypocreaceae | Hypocreales |
| **CX-CU49** | GMBCC1485 | PV660892 | Trichoderma sp. isolate 769_E5 | 100.00% | Hypocreaceae | Hypocreales |
| **CX-CU50** | GMBCC1486 | PV660893 | *Trichoderma virens* isolate TU | 100.00% | Hypocreaceae | Hypocreales |
| **CX-CU51** | GMBCC1487 | PV660894 | Trichoderma tomentosum isolate EI 1000 ep | 99.66% | Hypocreaceae | Hypocreales |
| **CX-CU52** | GMBCC1488 | PV660895 | Fusarium acaciae-mearnsii isolate NMX8 | 99.81% | Nectriaceae | Hypocreales |
| **CX-CU53** | GMBCC1489 | PV660896 | Trichoderma sp. isolate P0-058 | 100.00% | Hypocreaceae | Hypocreales |
| **CX-CU54** | GMBCC1490 | PV660897 | Trichoderma sp. isolate P0-058 | 100.00% | Hypocreaceae | Hypocreales |
| **CX-CU55** | GMBCC1491 | PV660898 | Fusarium oxysporum clone SF_293 | 100.00% | Nectriaceae | Hypocreales |
| **CX-CU56** | GMBCC1492 | PV660899 | Trichoderma pyramidale strain HF3P20 | 99.83% | Hypocreaceae | Hypocreales |
| **CX-CU57** | GMBCC1493 | PV660900 | Mucor variicolumellatus isolate 14-W-1 | 99.83% | Mucoraceae | Mucorales |
| **CX-CU58** | GMBCC1494 | PV660901 | Penicillium pancosmium CIRM-BRFM 3759 | 99.82% | Aspergillaceae | Eurotiales |
| **CX-CU59** | GMBCC1495 | PV660902 | Trichoderma sp. isolate P0-020 | 100.00% | Hypocreaceae | Hypocreales |
| **CX-CU60** | GMBCC1496 | PV660903 | Talaromyces kendrickii P1291 | 99.82% | Aspergillaceae | Eurotiales |
| **CX-CU61** | GMBCC1497 | PV660904 | Mucor sp. isolate M1-0139 | 100.00% | Mucoraceae | Mucorales |
| **CX-CU62** | GMBCC1498 | PV660905 | Mucor sp. isolate M1-0139 | 100.00% | Mucoraceae | Mucorales |
| **CX-CU63** | GMBCC1499 | PV660906 | *Gliomastix murorum* CBS:154.25 | 99.82% | Bionectriaceae | Hypocreales |
| **CX-CU64** | GMBCC1500 | PV660907 | Trichoderma sp. Ir.330 | 99.83% | Hypocreaceae | Hypocreales |
| **CX-CU65** | GMBCC1501 | PV660908 | Trichoderma sp. isolate P0-058 | 100.00% | Hypocreaceae | Hypocreales |
| **CX-CU66** | GMBCC1502 | PV660909 | Trichoderma sp. isolate P0-026 | 100.00% | Hypocreaceae | Hypocreales |
| **CX-CY1** | GMBCC1503 | PV660910 | Trichoderma sp. isolate P0-026 | 100.00% | Hypocreaceae | Hypocreales |
| **CX-CY2** | GMBCC1504 | PV660911 | Trichoderma sp. isolate P0-026 | 100.00% | Hypocreaceae | Hypocreales |
| **CX-CY3** | GMBCC1505 | PV660912 | *Mucor* sp. isolate CB21-002 | 100.00% | Mucoraceae | Mucorales |
| **CX-CY4** | GMBCC1506 | PV660913 | Chaetomium sp. isolate P4-003 | 100.00% | Chaetomiaceae | Sordariales |
| **CX-CY5** | GMBCC1507 | PV660914 | *Gliomastix murorum* CBS:154.25 | 99.82% | Bionectriaceae | Hypocreales |
| **CX-CY6** | GMBCC1508 | PV660915 | Trichoderma sp. isolate 769_E5 | 100.00% | Hypocreaceae | Hypocreales |
| **CX-CY7** | GMBCC1509 | PV660916 | Mucor sp. strain 2583-3 | 100.00% | Mucoraceae | Mucorales |
| **CX-CY8** | GMBCC1510 | PV660917 | *Aspergillus* sp. strain CMRP4511 | 99.65% | Aspergillaceae | Eurotiales |
| **CX-CY9** | GMBCC1511 | PV660918 | Mucor sp. strain 2583-3 | 100.00% | Mucoraceae | Mucorales |
| **CX-CY10** | GMBCC1512 | PV660919 | Trichoderma sp. strain NLH-7 | 100.00% | Hypocreaceae | Hypocreales |
| **CX-CY11** | GMBCC1513 | PV660920 | Mucor sp. strain 2583-3 | 100.00% | Mucoraceae | Mucorales |
| **CX-CY12** | GMBCC1514 | PV660921 | Trichoderma sp. strain NLH-7 | 100.00% | Hypocreaceae | Hypocreales |
| **CX-CY13** | GMBCC1515 | PX548870 | Mucor sp. strain 2583-3 | 100.00% | Mucoraceae | Mucorales |
| **CX-CY14** | GMBCC1516 | PV660923 | Trichoderma sp. strain NLH-7 | 100.00% | Hypocreaceae | Hypocreales |
| **CX-CY15** | GMBCC1517 | PV660924 | *Trichoderma* sp. isolate 769_E5 | 100.00% | Hypocreaceae | Hypocreales |
| **CX-CY16** | GMBCC1518 | PV660925 | *Trichoderma* sp. isolate 769_E5 | 100.00% | Hypocreaceae | Hypocreales |
| **CX-CY17** | GMBCC1519 | PV660926 | Mucor sp. strain 2583-3 | 100.00% | Mucoraceae | Mucorales |
| **CX-CY18** | GMBCC1520 | PV660927 | Fusarium oxysporum clone SF_293 | 100.00% | Nectriaceae | Hypocreales |
| **CX-CY19** | GMBCC1521 | PV660928 | *Pleurostoma ootheca* isolate NS1a111 | 99.43% | Pleurostomataceae | Calosphaeriales |
| **CX-CY20** | GMBCC1522 | PV660929 | Trichoderma sp. strain SP3 | 100.00% | Hypocreaceae | Hypocreales |
| **CX-CY21** | GMBCC1523 | PV660930 | Fusarium oxysporum clone SF_293 | 100.00% | Nectriaceae | Hypocreales |
| **CX-CY22** | GMBCC1524 | PV660931 | Trichoderma sp. CHTAM10 | 100.00% | Hypocreaceae | Hypocreales |
| **CX-CY23** | GMBCC1525 | PV660932 | Paecilomyces sp. JCM 12545 | 98.60% | Aspergillaceae | Eurotiales |
| **CX-CY24** | GMBCC1526 | PV660933 | Fusarium oxysporum clone SF_293 | 100.00% | Nectriaceae | Hypocreales |
| **CX-CY25** | GMBCC1527 | PV660934 | Scedosporium apiospermum B11CPSc26 | 100.00% | Microascaceae | Microascales |
| **CX-CY26** | GMBCC1528 | PV660935 | Trichoderma aureoviride CCTCC:AV487 | 100.00% | Hypocreaceae | Hypocreales |
| **CX-CY27** | GMBCC1529 | PV660936 | Sporothrix narcissi isolate TF35 | 97.92% | Ophiostomataceae | Ophiostomatales |
| **CX-CY28** | GMBCC1530 | PV660937 | *Mucor septatus*strain URM7364_RV02 | 97.24% | Mucoraceae | Mucorales |
| **CX-CY29** | GMBCC1531 | PV660938 | Preussia sp. isolate Tw3.2 | 100.00% | Sporormiaceae | Pleosporales |
| **CX-CY30** | GMBCC1532 | PV660939 | Umbelopsis isabellina MKJP2012_3 31_2012 | 100.00% | Umbelopsidaceae | Umbelopsidales |
| **CX-CY31** | GMBCC1533 | PV660940 | Umbelopsis sp. isolate U218 | 99.66% | Umbelopsidaceae | Umbelopsidales |
| **CX-CY32** | GMBCC1534 | PV660941 | Dothiorella sp. EB-2020a | 100.00% | Botryosphaeriaceae | Botryosphaeriales |
| **CX-CY33** | GMBCC1535 | PV660942 | Umbelopsis sp. isolate U218 | 99.67% | Umbelopsidaceae | Umbelopsidales |
| **CX-CY34** | GMBCC1536 | PV660943 | *Mucor variicolumellatus* isolate 14-W-1 | 99.83% | Mucoraceae | Mucorales |
| **CX-CY35** | GMBCC1375 | PV644087 | *Martininia panamaensis* CBS 207.47 | 97.17% | Sclerotiniaceae | Helotiales |
| **CX-CY36** | GMBCC1376 | PV644088 | *Aspergillus tubingensis*CNU081066 | 100.00% | Aspergillaceae | Eurotiales |
| **CX-CY37** | GMBCC1377 | PV644089 | *Trichoderma atroviride* strain ZNWPL4 | 100.00% | Hypocreaceae | Hypocreales |
| **CX-CY38** | GMBCC1378 | PV644090 | *Trichoderma viridescens* CCTCC:AO400 | 99.83% | Hypocreaceae | Hypocreales |
| **CX-CY39** | GMBCC1379 | PV644091 | *Trichoderma cf. harzianum* PDA N56-1 | 100% | Hypocreaceae | Hypocreales |
| **CX-CY40** | GMBCC1380 | PV644092 | *Peroneutypa* sp. LL-2024a | 99.83% | Diatrypaceae | Xylariales |
| **CX-CY41** | GMBCC1381 | PV644093 | *Paecilomyces* sp. JCM 12545 | 100.00% | Aspergillaceae | Eurotiales |
| **CX-CY42** | GMBCC1382 | PV644094 | *Trichoderma harzianum* KIMM-1007 | 100.00% | Hypocreaceae | Hypocreales |
| **CX-CY43** | GMBCC1383 | PV644095 | *Fusarium oxysporum* UACH-222 | 100.00% | Nectriaceae | Hypocreales |
| **CX-CY44** | GMBCC1384 | PV644096 | *Trichoderma velutinum* T8KSU | 99.83% | Hypocreaceae | Hypocreales |
| **CX-CY45** | GMBCC1385 | PV644097 | *Trichoderma harzianum* ILSO1_4_25 | 100.00% | Hypocreaceae | Hypocreales |
| **CX-CY46** | GMBCC1386 | PV644098 | *Saksenaea* sp. strain 18AZ-3C | 100.00% | Saksenaeaceae | Mucorales |
| **CX-CY47** | GMBCC1387 | PV644099 | *Trichoderma sulphureum* SF_15 | 100.00% | Hypocreaceae | Hypocreales |
| **CX-CY48** | GMBCC1388 | PV644100 | *Trichoderma v*iride P78 | 100.00% | Hypocreaceae | Hypocreales |
| **CX-CY49** | GMBCC1389 | PV644101 | *Trichoderma* sp. CY-2024c isolate D4-2-1 | 99.66% | Hypocreaceae | Hypocreales |
| **CX-CY50** | GMBCC1390 | PV644102 | *Trichoderma* sp. isolate ST0-001 | 100.00% | Hypocreaceae | Hypocreales |
| **CX-CY51** | GMBCC1391 | PV644103 | *Mortierella* sp. A736 | 100.00% | Mortierellaceae | Mortierellales |
| **CX-CY52** | GMBCC1392 | PV644104 | *Mucor irregularis* isolate 80454 | 100.00% | Mucoraceae | Mucorales |
| **CX-CY53** | GMBCC1393 | PV644105 | *Phaeosphaeria* sp. strain CN136G3 | 99.83% | Phaeosphaeriaceae | Pleosporales |
| **CX-CY54** | GMBCC1394 | PV644106 | *Trichoderma harzianum* strain ZWPUEB10 | 100.00% | Hypocreaceae | Hypocreales |
| **CX-CY55** | GMBCC1395 | PV644107 | *Mucor plumbeus* isolate R97306 | 100.00% | Mucoraceae | Mucorales |
| **CX-CY56** | GMBCC1396 | PV644108 | *Gongronella* sp. strain SAUCC 4059-1 | 100.00% | Cunninghamellaceae | Mucorales |
| **CX-CY57** | GMBCC1397 | PV644109 | *Trichoderma* sp. CY-2024b isolate H5-1 | 100.00% | Hypocreaceae | Hypocreales |
| **CX-CY58** | GMBCC1398 | PV644110 | *Trichoderma* sp. CY-2024c | 100.00% | Hypocreaceae | Hypocreales |
| **CX-CY59** | GMBCC1399 | PV644111 | *Preussia* sp. isolate 29 | 100.00% | Sporormiaceae | Pleosporales |
| **CX-CY60** | GMBCC1400 | PV644112 | *Chaetomium* sp. clone YB1605 | 100.00% | Chaetomiaceae | Sordariales |
| **CX-CY61** | GMBCC1401 | PV644113 | *Phialomyces arenicola* isolate ANR2 | 99.65% | Aspergillaceae | Eurotiales |
| **CX-CY62** | GMBCC1402 | PV644114 | *Gliocladiopsis* sp. strain Bpf-2 | 99.81% | Nectriaceae | Hypocreales |
| **CX-CY63** | GMBCC1403 | PV644115 | *Umbelopsis isabellina* CM14_RG68 | 100% | Umbelopsidaceae | Umbelopsidales |
| **CX-CY64** | GMBCC1404 | PV644116 | *Mucor variicolumellatus* 14-W-1 | 100.00% | Mucoraceae | Mucorales |
| **CX-CY65** | GMBCC1405 | PV644117 | *Scleroconidioma sphagnicola* isolate 397J14 | 99.64% | *Incertae sedis* | *Incertae sedis* |

**Table S4.** BLASTn identification of the 200 fungal strains isolated from the fungus combs of *Macrotermes gilvus* colonies. (Colony 1: Cmu-a1-a57; Colony 2: Cmu-b1-b67; Colony 3: Cmu-c1-c76)

| **Isolates** | **Accession No.** | **BLASTn results (ITS)** | **Similarity** | **Family** | **Order** |
| --- | --- | --- | --- | --- | --- |
| **Cmu-a1** | PX443532 | Phomopsis sp. Y13 | 99.27% | Diaporthaceae | Diaporthales |
| **Cmu-a2** | PX443533 | *Curvularia verruculosa* strain M0239 | 99.82% | Pleosporaceae | Pleosporales |
| **Cmu-a3** | PX443534 | *Geotrichum* sp. 4-3 | 100% | Dipodascaceae | Saccharomycetales |
| **Cmu-a4** | PX443535 | *Cunninghamella* sp. strain XG09702-9-1 | 100% | Cunninghamellaceae | Mucorales |
| **Cmu-a5** | PX443536 | *Cunninghamella blakesleeana* CBS 133.27 | 100% | Cunninghamellaceae | Mucorales |
| **Cmu-a6** | PX443537 | *Bartalinia pondoensis* isolate CCTU 459 | 99.65% | Bartaliniaceae | Amphisphaeriales |
| **Cmu-a7** | PX443538 | *Peroneutypa* sp. isolate YTS23_1 | 99.45% | Diatrypaceae | Xylariales |
| **Cmu-a8** | PX443539 | *Aspergillus* sp. isolate BMF 16 | 99.82% | Aspergillaceae | Eurotiales |
| **Cmu-a9** | PX443540 | *Mucor aseptatophorus* strain FPFCC58ER-3 | 99.83% | Mucoraceae | Mucorales |
| **Cmu-a10** | PX443541 | *Trichosporon asahii* CBS:2479 | 100% | Trichosporonaceae | Trichosporonales |
| **Cmu-a11** | PX443542 | *Fusarium oxysporum* isolate L-4 | 99.06% | Nectriaceae | Hypocreales |
| **Cmu-a12** | PX443543 | *Trichoderma virens* isolate ACP-Tv13 | 100% | Hypocreaceae | Hypocreales |
| **Cmu-a13** | PX443544 | *Neopestalotiopsis mangiferae* isolate J4R1 | 100% | Pestalotiopsidaceae | Amphisphaeriales |
| **Cmu-a14** | PX443545 | *Curvularia lunata* CBS 730.96 | 100% | Pleosporaceae | Pleosporales |
| **Cmu-a15** | PX443546 | *Trichoderma virens* strain RF32 | 100% | Hypocreaceae | Hypocreales |
| **Cmu-a16** | PX443547 | *Trichoderma virens* isolate ACP-Tv13 | 100% | Hypocreaceae | Hypocreales |
| **Cmu-a17** | PX443548 | *Penicillium* sp. strain SW2B | 100% | Aspergillaceae | Eurotiales |
| **Cmu-a18** | PX443549 | *Trichoderma longibrachiatum* isolate 2916 | 100% | Hypocreaceae | Hypocreales |
| **Cmu-a19** | PX443550 | *Trichoderma harzianum* strain SKCGW012 | 100% | Hypocreaceae | Hypocreales |
| **Cmu-a20** | PX443551 | *Diaporthe raonikayaporum* MFLUCC 14-1136 | 100% | Diaporthaceae | Diaporthales |
| **Cmu-a21** | PX443552 | *Fusarium solani* isolate XX-1 | 100% | Nectriaceae | Hypocreales |
| **Cmu-a22** | PX443553 | *Trichoderma virens* clone SF_760 | 100% | Hypocreaceae | Hypocreales |
| **Cmu-a23** | PX443554 | *Cunninghamella blakesleeana* CBS 133.27 | 100% | Cunninghamellaceae | Mucorales |
| **Cmu-a24** | PX443555 | *Trichoderma virens* isolate ACP-Tv13 | 100% | Hypocreaceae | Hypocreales |
| **Cmu-a25** | PX443556 | *Thielavia* sp. strain CBI-S002 | 86% | Ceratocystidaceae | Microascales |
| **Cmu-a26** | PX443557 | *Scolecobasidium cordanae* isolate CF00102 | 99.67% | Sympoventuriaceae | enturiales |
| **Cmu-a27** | PX443558 | *Trichoderma harzianum* strain CFAM-422 | 100% | Hypocreaceae | Hypocreales |
| **Cmu-a28** | PX443559 | *Trichoderma atroviride* TATWM-IQ | 99.67% | Hypocreaceae | Hypocreales |
| **Cmu-a29** | PX443560 | *Trichoderma reesei* strain 10-56 TD1 | 100% | Hypocreaceae | Hypocreales |
| **Cmu-a30** | PX443561 | *Lasiodiplodia pseudotheobromae* isolate HSW-2 | 100% | Botryosphaeriaceae | Botryosphaeriales |
| **Cmu-a31** | PX443562 | *Curvularia sorghina* strain SFC20220920-G189 | 99.81% | Pleosporaceae | Pleosporales |
| **Cmu-a32** | PX443563 | *Curvularia* sp. isolate UgN58 | 100% | Pleosporaceae | Pleosporales |
| **Cmu-a33** | PX443564 | *Aspergillus niger* isolate RDE293 | 100% | Aspergillaceae | Eurotiales |
| **Cmu-a34** | PX443565 | *Bartalinia pondoensis* isolate CCTU 459 | 100% | Bartaliniaceae | Amphisphaeriales |
| **Cmu-a35** | PX443566 | *Trichoderma atroviride* strain ZNWPL10 | 100% | Hypocreaceae | Hypocreales |
| **Cmu-a36** | PX443567 | *Trichoderma longibrachiatum* XZ N205-1 | 99.83% | Hypocreaceae | Hypocreales |
| **Cmu-a37** | PX443568 | *Mucor irregularis* isolate UASWS2622 | 99.83% | Mucoraceae | Mucorales |
| **Cmu-a38** | PX443569 | *Cunninghamella* sp. strain XG09702-9-1 | 100% | Cunninghamellaceae | Mucorales |
| **Cmu-a39** | PX443570 | *Curvularia* sp. strain M1380 | 100% | Pleosporaceae | Pleosporales |
| **Cmu-a40** | PX443571 | *Neopestalotiopsis chiangmaiensis* CGMCC 3.24340 | 100% | Pestalotiopsidaceae | Amphisphaeriales |
| **Cmu-a41** | PX443572 | *Lasiodiplodia* sp. KR1 | 100% | Botryosphaeriaceae | Botryosphaeriales |
| **Cmu-a42** | PX443573 | *Trichoderma harzianum* isolate ARCF99 | 99.83% | Hypocreaceae | Hypocreales |
| **Cmu-a43** | PX443574 | *Trichoderma* sp. strain NTS-11 | 100% | Hypocreaceae | Hypocreales |
| **Cmu-a44** | PX443575 | *Neopestalotiopsis* sp. isolate FX26 | 100% | Pestalotiopsidaceae | Amphisphaeriales |
| **Cmu-a45** | PX443576 | *Curvularia* sp. strain M1380 | 99.82% | Pleosporaceae | Pleosporales |
| **Cmu-a46** | PX443577 | *Trichoderma lixii* culture CCTCC:AO422 | 99.82% | Hypocreaceae | Hypocreales |
| **Cmu-a47** | PX443578 | *Pseudocercospora fuliginosa* SZJB23-46 | 100% | Mycosphaerellaceae | Mycosphaerellales |
| **Cmu-a48** | PX443579 | *Trichoderma aureoviride* strain SKCGW013 | 100% | Hypocreaceae | Hypocreales |
| **Cmu-a49** | PX443580 | *Trichoderma atroviride* TATWM-IQ | 99.82% | Hypocreaceae | Hypocreales |
| **Cmu-a50** | PX443581 | *Trichoderma* sp. strain NSU-23 | 99.83% | Hypocreaceae | Hypocreales |
| **Cmu-a51** | PX443582 | *Curvularia verruculosa* isolate Phaseolus vulgaris | 100% | Pleosporaceae | Pleosporales |
| **Cmu-a52** | PX443583 | *Trichoderma atroviride* TATWM-I | 99% | Hypocreaceae | Hypocreales |
| **Cmu-a53** | PX443584 | *Trichoderma virens* isolate ACP-Tv13 | 100% | Hypocreaceae | Hypocreales |
| **Cmu-a54** | PX443585 | *Cerrena* sp. isolate Lyc23 | 99.84% | Cerrenaceae | Polyporales |
| **Cmu-a55** | PX443586 | *Fusarium oxysporum* strain MIAE01274 | 100% | Nectriaceae | Hypocreales |
| **Cmu-a56** | PX443587 | *Cercospora cf. malloti CPC 20737* | 99.60 | Mycosphaerellaceae | Mycosphaerellales |
| **Cmu-a57** | PX443588 | *Trichoderma* sp. isolate 243_A4 | 99.83% | Hypocreaceae | Hypocreales |
| **Cmu-b1** | PX548871 | *Rhizopus* sp. isolate CU47 | 100% | Rhizopodaceae | Mucorales |
| **Cmu-b2** | PX548872 | *Neopestalotiopsis* sp. XL-2025d | 100% | Pestalotiopsidaceae | Amphisphaeriales |
| **Cmu-b3** | PX548873 | *Trichoderma* sp. isolate K26 | 100% | Hypocreaceae | Hypocreales |
| **Cmu-b4** | PX548874 | *Fusarium oxysporum isolate* DSM 106834 | 100% | Nectriaceae | Hypocreales |
| **Cmu-b5** | PX548875 | *Aspergillus* sp. isolate 1-1 | 100% | Aspergillaceae | Eurotiales |
| **Cmu-b6** | PX548876 | *Neopestalotiopsis* sp. isolate FX27 | 100% | Pestalotiopsidaceae | Amphisphaeriales |
| **Cmu-b7** | PX548877 | *Neopestalotiopsis* sp. isolate MFTU41 | 100% | Pestalotiopsidaceae | Amphisphaeriales |
| **Cmu-b8** | PX548878 | *Aspergillus flavus* isolate AMS_3 | 100% | Aspergillaceae | Eurotiales |
| **Cmu-b9** | PX548879 | *Neopestalotiopsis chiangmaiensis* CGMCC 3.24340 | 100% | Pestalotiopsidaceae | Amphisphaeriales |
| **Cmu-b10** | PX548880 | *Leptoxyphium fumago* strain M50 | 98.8% | Capnodiaceae | Capnodiales |
| **Cmu-b11** | PX548881 | *Neoleptodontidium* sp. isolate OS5PP3N4SBAN | 96.94% | Incertae sedis | Xylariales |
| **Cmu-b12** | PX548882 | *Fusarium* sp. isolate HSB6 | 99.81% | Nectriaceae | Hypocreales |
| **Cmu-b13** | PX548883 | *Chondrostereum* sp. isolate NTOU5303 | 99.47% | Cyphellaceae | Agaricales |
| **Cmu-b14** | PX548884 | *Trichoderma lixii* culture CCTCC:AO422 | 99.82% | Hypocreaceae | Hypocreales |
| **Cmu-b15** | PX548885 | *Fusarium falciforme* isolate TJS-23-2 | 100% | Nectriaceae | Hypocreales |
| **Cmu-b16** | PX548886 | *Coprinopsis rhombisporoides BR DDCOFRI* | 97.92% | Psathyrellaceae | Agaricales |
| **Cmu-b17** | PX548887 | *Fusarium solani* isolate Shuhb-5 | 100% | Nectriaceae | Hypocreales |
| **Cmu-b18** | PX548888 | *Fusarium solani* isolate Shuhb-5 | 100% | Nectriaceae | Hypocreales |
| **Cmu-b19** | PX548889 | *Fusarium solani* isolate Shuhb-5 | 100% | Nectriaceae | Hypocreales |
| **Cmu-b20** | PX548890 | *Trichoderma atroviride* strain CL | 99.93% | Hypocreaceae | Hypocreales |
| **Cmu-b21** | PX548891 | *Curvularia* sp. isolate UgN58 | 100% | Pleosporaceae | Pleosporales |
| **Cmu-b22** | PX548892 | *Nigrohirschioporus durus v*oucher SL1997 | 100% | Hirschioporaceae | Hymenochaetales |
| **Cmu-b23** | PX548893 | *Trichoderma aureoviride* strain SKCGW013 | 100% | Hypocreaceae | Hypocreales |
| **Cmu-b24** | PX548894 | *Penicillium citrinum* isolate SM081 | 100% | Aspergillaceae | Eurotiales |
| **Cmu-b25** | PX548895 | *Aspergillus nomiae* isolate USPMTOX123 | 100% | Aspergillaceae | Eurotiales |
| **Cmu-b26** | PX548896 | *Fusarium solani* isolate Shuhb-5 | 99.62% | Nectriaceae | Hypocreales |
| **Cmu-b27** | PX548897 | *Phanerochaete* sp. T172A | 99.83% | Phanerochaetaceae | Polyporales |
| **Cmu-b28** | PX548898 | *Fusarium* solani isolate Shuhb-5 | 100% | Nectriaceae | Hypocreales |
| **Cmu-b29** | PX548899 | *Aspergillus nomiae* isolate USPMTOX119 | 99.82% | Aspergillaceae | Eurotiales |
| **Cmu-b30** | PX548900 | *Fusarium solani* isolate DCR09 | 100% | **Nectriaceae** | Hypocreales |
| **Cmu-b31** | PX548901 | *Penicillium* sp. isolate Cmu-c59 | 100% | Aspergillaceae | Eurotiales |
| **Cmu-b32** | PX548902 | *Aspergillus* sp. isolate B261 | 99.82% | Aspergillaceae | Eurotiales |
| **Cmu-b33** | PX548903 | *Gongronella* sp. strain CBI-S023 | 100% | Cunninghamellaceae | Mucorales |
| **Cmu-b34** | PX548904 | *Saksenaea* sp. isolate AOc4 | 99.86% | Saksenaeaceae | Mucorales |
| **Cmu-b35** | PX548905 | *Lasiodiplodia theobromae* isolate B1f | 100% | Botryosphaeriaceae | Botryosphaeriales |
| **Cmu-b36** | PX548906 | *Acremonium polychromum* strain SZWE1 | 100% | Bionectriaceae | Hypocreales |
| **Cmu-b37** | PX548907 | *Phomopsis* sp. PSU-ES62 | 99.45% | Diaporthaceae | Diaporthales |
| **Cmu-b38** | PX548908 | *Neopestalotiopsis chrysea* voucher JUF0010 | 100% | Pestalotiopsidaceae | Amphisphaeriales |
| **Cmu-b39** | PX548909 | *Setophaeosphaeria microspora* isolate A2-17 | 99.32% | Phaeosphaeriaceae | Pleosporales |
| **Cmu-b40** | PX548910 | *Cytospora* sp. isolate F11 | 99.63% | Cytosporaceae | Diaporthales |
| **Cmu-b41** | PX548911 | *Acremonium polychromum* isolate J30 | 100% | Bionectriaceae | Hypocreales |
| **Cmu-b42** | PX548912 | *Humicola phialophoroides* strain 24MA3F-3 | 99.63% | Chaetomiaceae | Sordariales |
| **Cmu-b43** | PX548913 | *Scedosporium dehoogii isolate* CMRP2880 | 99.31% | Microascaceae | Microascales |
| **Cmu-b44** | PX548914 | *Aspergillus nomiae* isolate USPMTOX132 | 100% | Aspergillaceae | Eurotiales |
| **Cmu-b45** | PX548915 | *Phaeosphaeriopsis musae* strain SZWE5 | 100% | Phaeosphaeriaceae | Pleosporales |
| **Cmu-b46** | PX548916 | *Curvularia lunata* strain QLCL2.5 | 100% | Pleosporaceae | Pleosporales |
| **Cmu-b47** | PX548917 | *Fusarium solani* strain T-ICA06NP | 100% | **Nectriaceae** | Hypocreales |
| **Cmu-b48** | PX548918 | *Cunninghamella* sp. strain XG09702-9-1 | 100% | Cunninghamellaceae | Mucorales |
| **Cmu-b49** | PX548919 | *Geotrichum* sp. 4-3 | 100% | Dipodascaceae | Saccharomycetales |
| **Cmu-b50** | PX548920 | *Cunninghamella blakesleeana* CBS 133.27 | 100.00% | Cunninghamellaceae | Mucorales |
| **Cmu-b51** | PX548921 | *Scedosporium dehoogii* strain DMic 01867 | 99.65% | Microascaceae | Microascales |
| **Cmu-b52** | PX548922 | *Aspergillus niger* strain APBSWTPF64 | 99.82% | Aspergillaceae | Eurotiales |
| **Cmu-b53** | PX548923 | *Scedosporium dehoogii* strain DMic 01867 | 99.65% | Microascaceae | Microascales |
| **Cmu-b54** | PX548924 | *Trichoderma erinaceum* strain CIB T72 | 99.82% | Hypocreaceae | Hypocreales |
| **Cmu-b55** | PX548925 | *Talaromyces pinophilus* isolate R14 | 100% | Aspergillaceae | Eurotiales |
| **Cmu-b56** | PX548926 | *Aspergillus nomius* strain S1 | 99.82% | Aspergillaceae | Eurotiales |
| **Cmu-b57** | PX548927 | *Lasiodiplodia theobromae isolate YLH2-2* | 100% | Botryosphaeriaceae | Botryosphaeriales |
| **Cmu-b58** | PX548928 | *Aspergillus costaricensis* isolate PM7 | 100% | Aspergillaceae | Eurotiales |
| **Cmu-b59** | PX548929 | *Penicillium* sp. 28 BRO-2013 | 100% | Aspergillaceae | Eurotiales |
| **Cmu-b60** | PX548930 | *Metarhizium anisopliae* 090904-2-3 | 99.60% | Clavicipitaceae | Hypocreales |
| **Cmu-b61** | PX548931 | *Trichoderma* sp. voucher 47S | 100% | Hypocreaceae | Hypocreales |
| **Cmu-b62** | PX548932 | *Trichoderma* harzianum isolate WDPB2 | 100% | Hypocreaceae | Hypocreales |
| **Cmu-b63** | PX548933 | *Pleurostoma ootheca* CBS:126089 | 100% | Pleurostomataceae | Calosphaeriales |
| **Cmu-b64** | PX548934 | *Scedosporium dehoogii* strain GZUIFR21.837 | 98.99% | Microascaceae | Microascales |
| **Cmu-b65** | PX548935 | *Aspergillus quadrilineatus* strain KU20018.62 | 100% | Aspergillaceae | Eurotiales |
| **Cmu-b66** | PX548936 | *Humicola* sp. strain BRPET34 | 99.85% | Chaetomiaceae | Sordariales |
| **Cmu-b67** | PX548937 | *Coprinellus radians* 66087 | 99.53% | Psathyrellaceae | Agaricales |
| **Cmu-c1** | PX207373 | *Curvularia sorghina* SFC20220920-G189 | 100% | Pleosporaceae | Pleosporales |
| **Cmu-c2** | PX207374 | *Epicoccum* sp. 2 NV-2016 | 100% | Didymellaceae | Pleosporales |
| **Cmu-c3** | PX207375 | *Neopestalotiopsis* sp. GUCC 21003 | 99.81% | Pestalotiopsidaceae | Amphisphaeriales |
| **Cmu-c4** | PX207376 | *Hyphomucor assamensis* CBS: 415.77 | 95.75% | Mucoraceae | Mucorales |
| **Cmu-c5** | PX207377 | *Lasiodiplodia pseudotheobromae* HSW-2 | 100% | Botryosphaeriaceae | Botryosphaeriales |
| **Cmu-c6** | PX207378 | *Trichoderma breve* TWS48Abf(b) | 100% | Hypocreaceae | Hypocreales |
| **Cmu-c7** | PX207379 | *Thielaviopsis ethacetica* VPCI 1503/15 | 100% | Ceratocystidaceae | Microascales |
| **Cmu-c8** | PX207380 | *Trichoderma aureoviride* SKCGW013 | 100% | Hypocreaceae | Hypocreales |
| **Cmu-c9** | PX207381 | *Fusarium equiseti* ICMP 10350 | 100% | Nectriaceae | Hypocreales |
| **Cmu-c10** | PX207382 | *Macrophomina phaseolina* KWF09 | 100% | Botryosphaeriaceae | Botryosphaeriales |
| **Cmu-c11** | PX207383 | *Nigrospora sphaerica* AME-105 | 100% | Apiosporaceae | Amphisphaeriales |
| **Cmu-c12** | PX207384 | *Curvularia verruculosa* PSU-ES201 | 99.82% | Pleosporaceae | Pleosporales |
| **Cmu-c13** | PX207385 | *Aspergillus oryzae* strain PX-2 | 100% | Aspergillaceae | Eurotiales |
| **Cmu-c14** | PX207386 | *Phomopsis* sp. PSU-ES62 | 99.62% | Diaporthaceae | Diaporthales |
| **Cmu-c15** | PX207387 | *Pestalotiopsis mangiferae* isolate J4R1 | 100% | Pestalotiopsidaceae | Amphisphaeriales |
| **Cmu-c16** | PX207388 | *Curvularia verruculosa* PSU-ES201 | 100% | Pleosporaceae | Pleosporales |
| **Cmu-c17** | PX207389 | *Fusarium solani* strain B25 | 99.81% | Nectriaceae | Hypocreales |
| **Cmu-c18** | PX207390 | *Fusarium solani* isolate DCR09 | 99.63% | Nectriaceae | Hypocreales |
| **Cmu-c19** | PX207391 | *Trichoderma harzianum* strain ZNAF10 | 99.83% | Hypocreaceae | Hypocreales |
| **Cmu-c20** | PX207392 | *Trichoderma harzianum* ZNCF17 | 100% | Hypocreaceae | Hypocreales |
| **Cmu-c21** | PX207393 | *Neopestalotiopsis* sp. GUCC 21003 | 100% | Pestalotiopsidaceae | Amphisphaeriales |
| **Cmu-c22** | PX207394 | *Trichoderma virens* HGUP194023 | 100% | Hypocreaceae | Hypocreales |
| **Cmu-c23** | PX207395 | *Lichtheimia* sp. isolate 1TW9 | 100% | Lichtheimiaceae | Mucorales |
| **Cmu-c24** | PX207396 | *Neopestalotiopsis protearum* AME-127 | 100% | Pestalotiopsidaceae | Amphisphaeriales |
| **Cmu-c25** | PX207397 | *Gilbertella persicaria* culture CBS:325.71B | 99.67% | Choanephoraceae | Mucorales |
| **Cmu-c26** | PX207398 | *Aspergillus* sp. isolate HBJSFH-SD14 | 99.65% | Aspergillaceae | Eurotiales |
| **Cmu-c27** | PX207399 | *Fusarium solani* isolate G2 | 100% | Nectriaceae | Hypocreales |
| **Cmu-c28** | PX207400 | *Trichoderma longibrachiatum* isolate PM | 100% | Hypocreaceae | Hypocreales |
| **Cmu-c29** | PX207401 | *Trichoderma* sp. strain NTS-18 | 99.83% | Hypocreaceae | Hypocreales |
| **Cmu-c30** | PX207402 | *Trichoderma asperellum* strain STWT6 | 100% | Hypocreaceae | Hypocreales |
| **Cmu-c31** | PX207403 | *Trichoderma* sp. 1 MSP-2018 | 100% | Hypocreaceae | Hypocreales |
| **Cmu-c32** | PX207404 | *Trichoderma longibrachiatum* isolate 2925 | 100% | Hypocreaceae | Hypocreales |
| **Cmu-c33** | PX207405 | *Aspergillus niger* strain CPO MARH7 | 100% | Aspergillaceae | Eurotiales |
| **Cmu-c34** | PX207406 | *Trichoderma* sp. strain NTS-18 | 100% | Hypocreaceae | Hypocreales |
| **Cmu-c35** | PX207407 | *Penicillium sclerotiorum* isolate YY25 | 100% | Aspergillaceae | Eurotiales |
| **Cmu-c36** | PX207408 | *Trichoderma longibrachiatum* UASWS2629 | 100% | Hypocreaceae | Hypocreales |
| **Cmu-c37** | PX207409 | *Trichoderma virens* clone SF_29 | 99.83% | Hypocreaceae | Hypocreales |
| **Cmu-c38** | PX207410 | *Trichoderma* sp. BLT1C | 100% | Hypocreaceae | Hypocreales |
| **Cmu-c39** | PX207411 | *Trichoderma harzianum* ZNCF4 | 100% | Hypocreaceae | Hypocreales |
| **Cmu-c40** | PX207412 | *Epicoccum sorghinum* SFC102353 | 100% | Didymellaceae | Pleosporales |
| **Cmu-c41** | PX548938 | *Rhizopus arrhizus* Sun ITS01 Kz | 100% | Rhizopodaceae | Mucorales |
| **Cmu-c42** | PX207413 | *Trichoderma harzianum* strain Tr4 | 100% | Hypocreaceae | Hypocreales |
| **Cmu-c43** | PX207414 | *Trichoderma harzianum* strain Tr4 | 99.82% | Hypocreaceae | Hypocreales |
| **Cmu-c44** | PX207415 | *Lichtheimia brasiliensis* strain FB1 | 100% | Lichtheimiaceae | Mucorales |
| **Cmu-c45** | PX207416 | *Hyphomucor assamensis* CBS:415.77 | 95.68% | Mucoraceae | Mucorales |
| **Cmu-c46** | PX207417 | *Bartalinia pondoensis* isolate CCTU 459 | 99.82% | Bartaliniaceae | Amphisphaeriales |
| **Cmu-c47** | PX207418 | *Neopestalotiopsis mangiferae* isolate J4R1 | 100% | Pestalotiopsidaceae | Amphisphaeriales |
| **Cmu-c48** | PX207419 | *Lichtheimia* sp. isolate 1TW9 | 100% | Lichtheimiaceae | Mucorales |
| **Cmu-c49** | PX207420 | *Trichoderma virens* clone SF_29 | 99.83% | Hypocreaceae | Hypocreales |
| **Cmu-c50** | PX207421 | *Trichoderma* sp. isolate T2024 | 100% | Hypocreaceae | Hypocreales |
| **Cmu-c51** | PX207422 | *Penicillium citrinum* strain 217N | 100% | Aspergillaceae | Eurotiales |
| **Cmu-c52** | PX207423 | *Bjerkandera adusta* isolate NTOU5268 | 100% | Phanerochaetaceae | Polyporales |
| **Cmu-c53** | PX207424 | *Scedosporium apiospermum* B11CSHSc17 | 99.21% | Microascaceae | Microascales |
| **Cmu-c54** | PX207425 | *Penicillium sclerotiorum* isolate YY25 | 100% | Aspergillaceae | Eurotiales |
| **Cmu-c55** | PX207426 | *Talaromyces pinophilus* isolate P3 | 99.66% | Aspergillaceae | Eurotiales |
| **Cmu-c56** | PX207427 | *Pestalotiopsis* sp. YRX-2022b | 99.65% | Pestalotiopsidaceae | Amphisphaeriales |
| **Cmu-c57** | PX207428 | *Ectophoma multirostrata* strain M1417 | 100% | Didymellaceae | Pleosporales |
| **Cmu-c58** | PX207429 | *Ectophoma pomi* isolate A690 | 100% | Didymellaceae | Pleosporales |
| **Cmu-c59** | PX207430 | *Penicillium sclerotiorum* isolate CN13 | 97.78% | Aspergillaceae | Eurotiales |
| **Cmu-c60** | PX207431 | *Bartalinia pondoensis* isolate CCTU 459 | 99.82% | Bartaliniaceae | Amphisphaeriales |
| **Cmu-c61** | PX548939 | *Rhizopus arrhizus* Sun ITS01 Kz | 100% | Rhizopodaceae | Mucorales |
| **Cmu-c62** | PX207432 | *Chaetomium globosum* S1TH2 | 100% | Chaetomiaceae | Sordariales |
| **Cmu-c63** | PX207433 | *Trichoderma* sp. isolate T2024 | 100% | Hypocreaceae | Hypocreales |
| **Cmu-c64** | PX207434 | *Trichoderma* sp. strain NTS-18 | 99.82% | Hypocreaceae | Hypocreales |
| **Cmu-c65** | PX207435 | *Aspergillus* sp. isolate HBJSFH-SD14 | 100% | Aspergillaceae | Eurotiales |
| **Cmu-c66** | PX207436 | *Trichoderma asperellum* strain SHSJ2005 | 99.47% | Hypocreaceae | Hypocreales |
| **Cmu-c67** | PX207437 | *Trichoderma harzianum* ACCC33200 | 100% | Hypocreaceae | Hypocreales |
| **Cmu-c68** | PX548940 | *Rhizopus arrhizus* Sun ITS01 Kz | 100% | Rhizopodaceae | Mucorales |
| **Cmu-c69** | PX207438 | *Chaetomium globosum* HCPF 9215 | 100% | Chaetomiaceae | Sordariales |
| **Cmu-c70** | PX207439 | *Albifimbria verrucaria* strain M.v.1 | 99.82% | Stachybotryaceae | Hypocreales |
| **Cmu-c71** | PX207440 | *Absidia* sp. XJ-2024b | 99.17% | Cunninghamellaceae | Mucorales |
| **Cmu-c72** | PX207441 | *Purpureocillium lilacinum* HGUP190162 | 100% | Ophiocordycipitaceae | Hypocreales |
| **Cmu-c73** | PX207442 | *Lichtheimia brasiliensis* isolate Vimi-18.0001 | 100.00% | Lichtheimiaceae | Mucorales |
| **Cmu-c74** | PX207443 | *Trichoderma erinaceum* strain CEN1558 | 99.52% | Hypocreaceae | Hypocreales |
| **Cmu-c75** | PX207444 | *Talaromyces pinophilus* isolate P3 | 99.66% | Aspergillaceae | Eurotiales |
| **Cmu-c76** | PX207445 | *Leptoxyphium fumago* strain M50 | 99.80% | Capnodiaceae | Capnodiales |

**Table S5.** BLASTn identification of the 100 fungal strains isolated from worker guts of *Odontotermes* colonies, in Chuxiong, Yunnan, China. (Colony 1: QJ1-33, Colony 2: JJ1-33, Colony 3: QQ1-34)

| **Isolates** | **Collection No.** | **Accession No.** | **BLASTn results (ITS)** | **Similarity** | **Family** | **Order** |
| --- | --- | --- | --- | --- | --- | --- |
| **CX-QJ1** | GMBCC1206 | PV660733 | *Mucor* sp. isolate GMBCC1074 | 99.50% | Mucoraceae | Mucorales |
| **CX-QJ2** | GMBCC1207 | PV660734 | *Umbelopsis* sp. isolate U218 | 99.51% | Umbelopsidaceae | Umbelopsidales |
| **CX-QJ3** | GMBCC1208 | PV660735 | *Pestalotiopsis lushanensis* strain HF-080 | 99.83% | Pestalotiopsidaceae | Amphisphaeriales |
| **CX-QJ4** | GMBCC1209 | PV660736 | *Umbelopsis isabellina* CM14_RG68 | 100.00% | Umbelopsidaceae | Umbelopsidales |
| **CX-QJ5** | GMBCC1210 | PV660737 | *Pestalotiopsis lushanensis* strain HF-080 | 99.65% | Pestalotiopsidaceae | Amphisphaeriales |
| **CX-QJ6** | GMBCC1211 | PV660738 | *Mucor* sp. strain GMBCC1074 | 99.82% | Mucoraceae | Mucorales |
| **CX-QJ7** | GMBCC1212 | PV660739 | *Trichoderma tomentosum* isolate EI 1000 ep | 99.82% | Hypocreaceae | Hypocreales |
| **CX-QJ8** | GMBCC1213 | PV660740 | *Phaeosphaeria verruculosa* CBS 263.85 | 100.00% | Didymosphaeriaceae | Pleosporales |
| **CX-QJ9** | GMBCC1214 | PV660741 | *Trichoderma gamsii* isolate 40790-7S-1G-Fdi | 100.00% | Hypocreaceae | Hypocreales |
| **CX-QJ10** | GMBCC1215 | PV660742 | *Pestalotiopsis lushanensis* strain HF-080 | 100.00% | Pestalotiopsidaceae | Amphisphaeriales |
| **CX-QJ11** | GMBCC1216 | PV660743 | *Fusarium* sp. strain S331 | 99.81% | Nectriaceae | Hypocreales |
| **CX-QJ12** | GMBCC1217 | PV660744 | *Trichoderma* sp. strain P0-020 | 100.00% | Hypocreaceae | Hypocreales |
| **CX-QJ13** | GMBCC1218 | PV660745 | *Umbelopsis isabellina* isolate MKJP2012_3 31 | 99.04% | Umbelopsidaceae | Umbelopsidales |
| **CX-QJ14** | GMBCC1219 | PV660746 | *Preussia* sp. isolate Tw3.2 | 100.00% | Sporormiaceae | Pleosporales |
| **CX-QJ15** | GMBCC1220 | PV660747 | *Trichoderma gamsii* strain TW20050 | 100.00% | Hypocreaceae | Hypocreales |
| **CX-QJ16** | GMBCC1221 | PV660748 | *Linnemannia* sp. strain XG12234-1-1 | 100.00% | Mortierellaceae | Mortierellales |
| **CX-QJ17** | GMBCC1222 | PV660749 | *Mucor variicolumellatus* isolate 14-W-1 | 99.32% | Mucoraceae | Mucorales |
| **CX-QJ18** | GMBCC1223 | PV660750 | *Umbelopsis* sp. isolate XXMF000011 | 100.00% | Umbelopsidaceae | Umbelopsidales |
| **CX-QJ19** | GMBCC1224 | PV660751 | *Mucor* sp. strain GMBCC1074 | 100.00% | Mucoraceae | Mucorales |
| **CX-QJ20** | GMBCC1225 | PV660752 | *Linnemannia* sp. strain XG12234-1-1 | 100.00% | Mortierellaceae | Mortierellales |
| **CX-QJ21** | GMBCC1226 | PV660753 | *Linnemannia* sp. strain XG12234-1-1 | 100.00% | Mortierellaceae | Mortierellales |
| **CX-QJ22** | GMBCC1227 | PV660754 | *Trichoderma crassum* isolate JUFD106 | 99.83% | Hypocreaceae | Hypocreales |
| **CX-QJ23** | GMBCC1228 | PV660755 | *Trichoderma* sp. isolate 282_E4 | 99.66% | Hypocreaceae | Hypocreales |
| **CX-QJ24** | GMBCC1229 | PV660756 | *Trichoderma* sp. strain F0210-12L2 | 100.00% | Hypocreaceae | Hypocreales |
| **CX-QJ25** | GMBCC1230 | PV660757 | *Trichoderma* sp. isolate 282 | 99.66% | Hypocreaceae | Hypocreales |
| **CX-QJ26** | GMBCC1231 | PV660758 | *Trichoderma atroviride* isolate HZA13 | 100.00% | Hypocreaceae | Hypocreales |
| **CX-QJ27** | GMBCC1232 | PV660759 | *Trichoderma* sp. strain NFD10 | 100.00% | Hypocreaceae | Hypocreales |
| **CX-QJ28** | GMBCC1233 | PV660760 | *Trichoderma atroviride* isolate PAN12-12 | 100.00% | Hypocreaceae | Hypocreales |
| **CX-QJ29** | GMBCC1234 | PV660761 | *Trichoderma viridescens* CCTCC:AO400 | 99.82% | Hypocreaceae | Hypocreales |
| **CX-QJ30** | GMBCC1235 | PV660762 | *Mucor* sp. strain 2583-3 | 99.83% | Mucoraceae | Mucorales |
| **CX-QJ31** | GMBCC1236 | PV660763 | *Linnemannia* sp. strain XG12234-1-1 | 100.00% | Mortierellaceae | Mortierellales |
| **CX-QJ32** | GMBCC1237 | PV660764 | *Trichoderma koningiopsis* isolate A729 | 100.00% | Hypocreaceae | Hypocreales |
| **CX-QJ33** | GMBCC1238 | PV660765 | *Saksenaea* sp. strain 18AZ-3C | 100.00% | Saksenaeaceae | Mucorales |
| **CX-JJ1** | GMBCC1239 | PV660766 | *Linnemannia* sp. strain XG12234-1-1 | 100.00% | Mortierellaceae | Mortierellales |
| **CX-JJ2** | GMBCC1240 | PV660767 | *Trichoderma koningiopsis* CTCCSJ-AMH50461 | 100.00% | Hypocreaceae | Hypocreales |
| **CX-JJ3** | GMBCC1241 | PV660768 | *Linnemannia* sp. strain XG12234-1-1 | 100.00% | Mortierellaceae | Mortierellales |
| **CX-JJ4** | GMBCC1242 | PV660769 | *Trichoderma pubescens* clone SF_38 | 100.00% | Hypocreaceae | Hypocreales |
| **CX-JJ5** | GMBCC1243 | PV660770 | *Trichoderma harzianum* isolate KIMM-1007 | 100.00% | Hypocreaceae | Hypocreales |
| **CX-JJ6** | GMBCC1244 | PV660771 | *Mortierella* sp. strain YTM27 | 99.68% | Mortierellaceae | Mortierellales |
| **CX-JJ7** | GMBCC1245 | PV660772 | *Trichoderma koningiopsis* isolate A729 | 100.00% | Hypocreaceae | Hypocreales |
| **CX-JJ8** | GMBCC1246 | PV660773 | *Mucor chiangraiensis* MFLUCC 21-0042 | 99.83% | Mucoraceae | Mucorales |
| **CX-JJ9** | GMBCC1247 | PV660774 | *Linnemannia* sp. strain XG12234-1-1 | 100.00% | Mortierellaceae | Mortierellales |
| **CX-JJ10** | GMBCC1248 | PV660775 | *Mucor* sp. strain 2583-3 | 100.00% | Mucoraceae | Mucorales |
| **CX-JJ11** | GMBCC1249 | PV660776 | *Trichoderma* sp. strain CSC21B0576 | 100.00% | Hypocreaceae | Hypocreales |
| **CX-JJ12** | GMBCC1250 | PV660777 | *Trichoderma tomentosum* EI 1000 ep | 99.65% | Hypocreaceae | Hypocreales |
| **CX-JJ13** | GMBCC1251 | PV660778 | *Umbelopsis* sp. TKPB-2017 | 100.00% | Umbelopsidaceae | Umbelopsidales |
| **CX-JJ14** | GMBCC1252 | PV660779 | *Linnemannia* sp. strain XG12234-1-1 | 100.00% | Mortierellaceae | Mortierellales |
| **CX-JJ15** | GMBCC1253 | PV660780 | *Mucor* sp. strain 2583-3 | 100.00% | Mucoraceae | Mucorales |
| **CX-JJ16** | GMBCC1254 | PV660781 | *Mucor* sp. isolate GMBCC1074 | 99.66% | Mucoraceae | Mucorales |
| **CX-JJ17** | GMBCC1255 | PV660782 | *Gongronella* sp. strain SAUCC 4059-1 | 99.35% | Cunninghamellaceae | Mucorales |
| **CX-JJ18** | GMBCC1256 | PV660783 | *Mucor* sp. strain 2583-3 | 100.00% | Mucoraceae | Mucorales |
| **CX-JJ19** | GMBCC1257 | PV660784 | *Linnemannia* sp. strain XY05127 | 100.00% | Mortierellaceae | Mortierellales |
| **CX-JJ20** | GMBCC1258 | PV660785 | *Umbelopsis* sp. isolate XXMF000011 | 99.13% | Umbelopsidaceae | Umbelopsidales |
| **CX-JJ21** | GMBCC1259 | PV660786 | *Linnemannia* sp. strain XG12234-1-1 | 100.00% | Mortierellaceae | Mortierellales |
| **CX-JJ22** | GMBCC1260 | PV660787 | *Linnemannia* sp. strain XG12234-1-1 | 99.83% | Mortierellaceae | Mortierellales |
| **CX-JJ23** | GMBCC1261 | PV660788 | *Linnemannia* sp. strain XG12234-1-1 | 99.66% | Mortierellaceae | Mortierellales |
| **CX-JJ24** | GMBCC1262 | PV660789 | *Linnemannia* sp. strain XG12234-1-1 | 100.00% | Mortierellaceae | Mortierellales |
| **CX-JJ25** | GMBCC1263 | PV660790 | *Mucor* sp. isolate GMBCC1074 | 100.00% | Mucoraceae | Mucorales |
| **CX-JJ26** | GMBCC1264 | PV660791 | *Trichoderma* sp. isolate P0-058 | 99.83% | Hypocreaceae | Hypocreales |
| **CX-JJ27** | GMBCC1265 | PV660792 | *Mucor* sp. isolate GMBCC1074 | 99.78% | Mucoraceae | Mucorales |
| **CX-JJ28** | GMBCC1266 | PV660793 | *Linnemannia* sp. strain XG09531-3 | 100.00% | Mortierellaceae | Mortierellales |
| **CX-JJ29** | GMBCC1267 | PV660794 | *Linnemannia* sp. strain XG12234-1-1 | 99.83% | Mortierellaceae | Mortierellales |
| **CX-JJ30** | GMBCC1268 | PV660795 | *Linnemannia* sp. strain XG12234-1-1 | 99.82% | Mortierellaceae | Mortierellales |
| **CX-JJ31** | GMBCC1269 | PV660796 | *Linnemannia* sp. strain XG12234-1-1 | 100.00% | Mortierellaceae | Mortierellales |
| **CX-JJ32** | GMBCC1270 | PV660797 | *Linnemannia* sp. strain XG12234-1-1 | 100.00% | Mortierellaceae | Mortierellales |
| **CX-JJ33** | GMBCC1271 | PV660798 | *Mucor* sp. strain 2583-3 | 95.83% | Mucoraceae | Mucorales |
| **CX-QQ1** | GMBCC1272 | PV660799 | *Mortierella* sp. strain XY05130 | 99.83% | Mortierellaceae | Mortierellales |
| **CX-QQ2** | GMBCC1273 | PV660800 | *Mucor chiangraiensis* MFLUCC 21-0042 | 99.79% | Mucoraceae | Mucorales |
| **CX-QQ3** | GMBCC1274 | PV660801 | *Mucor* sp. strain 2583-3 | 99.83% | Mucoraceae | Mucorales |
| **CX-QQ4** | GMBCC1275 | PV660802 | *Linnemannia* sp. strain XG12234-1-1 | 100.00% | Mortierellaceae | Mortierellales |
| **CX-QQ5** | GMBCC1276 | PV660803 | *Linnemannia* sp. strain XG12234-1-1 | 100.00% | Mortierellaceae | Mortierellales |
| **CX-QQ6** | GMBCC1277 | PV660804 | *Trichoderma* sp. isolate P0-020 | 99.83% | Hypocreaceae | Hypocreales |
| **CX-QQ7** | GMBCC1278 | PV660805 | *Trichoderma koningiopsis* CCTCC:AV403 | 100.00% | Hypocreaceae | Hypocreales |
| **CX-QQ8** | GMBCC1279 | PV660806 | *Sporothrix narcissi* TF35 | 97.93% | Ophiostomataceae | Ophiostomatales |
| **CX-QQ9** | GMBCC1280 | PV660807 | *Mucor variicolumellatus* 14-W-1 | 99.83% | Mucoraceae | Mucorales |
| **CX-QQ10** | GMBCC1281 | PV660808 | *Linnemannia* sp. strain XG12234-1-1 | 100.00% | Mortierellaceae | Mortierellales |
| **CX-QQ11** | GMBCC1282 | PV660809 | *Mucor* sp. strain 2583-3 | 100.00% | Mucoraceae | Mucorales |
| **CX-QQ12** | GMBCC1283 | PV660810 | *Mucor* sp. strain 2583-3 | 100.00% | Mucoraceae | Mucorales |
| **CX-QQ13** | GMBCC1284 | PV660811 | *Linnemannia* sp. strain XG12234-1-1 | 100.00% | Mortierellaceae | Mortierellales |
| **CX-QQ14** | GMBCC1285 | PV660812 | *Trichoderma* sp. strain NSU-23 | 100.00% | Hypocreaceae | Hypocreales |
| **CX-QQ15** | GMBCC1286 | PV660813 | *Linnemannia* sp. strain XG12234-1-1 | 100.00% | Mortierellaceae | Mortierellales |
| **CX-QQ16** | GMBCC1287 | PV660814 | *Mucor* sp. strain 2583-3 | 99.83% | Mucoraceae | Mucorales |
| **CX-QQ17** | GMBCC1288 | PV660815 | *Gongronella* sp. strain SAUCC 4059-1 | 99.00% | Cunninghamellaceae | Mucorales |
| **CX-QQ18** | GMBCC1289 | PV660816 | *Mucor* sp. strain 2583-3 | 100.00% | Mucoraceae | Mucorales |
| **CX-QQ19** | GMBCC1290 | PV660817 | *Mucor* sp. strain 2583-3 | 99.83% | Mucoraceae | Mucorales |
| **CX-QQ20** | GMBCC1291 | PV660818 | *Mucor* sp. strain 2583-3 | 99.83% | Mucoraceae | Mucorales |
| **CX-QQ21** | GMBCC1292 | PV660819 | *Mucor* sp. strain 2583-3 | 95.73% | Mucoraceae | Mucorales |
| **CX-QQ22** | GMBCC1293 | PV660820 | *Trichoderma tomentosum* isolate 21 | 99.67% | Hypocreaceae | Hypocreales |
| **CX-QQ23** | GMBCC1294 | PV660821 | *Trichoderma guizhouense* BPI:GJS 08135 | 100.00% | Hypocreaceae | Hypocreales |
| **CX-QQ24** | GMBCC1295 | PV660822 | *Mucor* sp. isolate GMBCC1074 | 99.50% | Mucoraceae | Mucorales |
| **CX-QQ25** | GMBCC1296 | PV660823 | *Linnemannia* sp. strain XG09531-3 | 100.00% | Mortierellaceae | Mortierellales |
| **CX-QQ26** | GMBCC1297 | PV660824 | *Trichoderma* sp. Ir.330 | 99.66% | Hypocreaceae | Hypocreales |
| **CX-QQ27** | GMBCC1298 | PV660825 | *Mucor* sp. isolate E20376 | 99.83% | Mucoraceae | Mucorales |
| **CX-QQ28** | GMBCC1299 | PV660826 | *Linnemannia nantahalensis* CBS 610.70 | 100.00% | Mortierellaceae | Mortierellales |
| **CX-QQ29** | GMBCC1300 | PV660827 | *Mucor* sp. strain 2583-3 | 100.00% | Mucoraceae | Mucorales |
| **CX-QQ30** | GMBCC1301 | PV660828 | *Linnemannia* sp. strain XG12234-1-1 | 99.83% | Mortierellaceae | Mortierellales |
| **CX-QQ31** | GMBCC1302 | PV660829 | *Aspergillus niger* CBS:124.38 | 100.00% | Aspergillaceae | Eurotiales |
| **CX-QQ32** | GMBCC1303 | PV660830 | *Mucor* sp. isolate GMBCC1074 | 99.50% | Mucoraceae | Mucorales |
| **CX-QQ33** | GMBCC1304 | PV660831 | *Mucor* sp. isolate E20376 | 99.83% | Mucoraceae | Mucorales |
| **CX-QQ34** | GMBCC1305 | PV660832 | *Mucor* sp. isolate GMBCC1074 | 99.67% | Mucoraceae | Mucorales |

**Table S6.** BLASTn identification of the 100 fungal strains isolated from worker guts of *Macrotermes gilvus* colonies in Chiang Mai, Thailand. (Colony 1: ag1-32, Colony 1: bg1-30, Colony 1: cg1-38)

| **Isolates** | **Accession No.** | **BLASTn results (ITS)** | **Similarity** | **Family** | **Order** |
| --- | --- | --- | --- | --- | --- |
| **Cmu-ag1** | PX548941 | *Lasiodiplodia theobromae* isolate 29B | 100% | Botryosphaeriaceae | Botryosphaeriales |
| **Cmu-ag2** | PX548942 | *Talaromyces pinophilus* strain FS96 | 100% | Aspergillaceae | Eurotiales |
| **Cmu-ag3** | PX548943 | *Aspergillus caelatus* CBS:763.97 | 99.47% | Aspergillaceae | Eurotiales |
| **Cmu-ag4** | PX548944 | *Irpex latemarginatus* voucher HGUP192131 | 100% | Irpicaceae | Polyporales |
| **Cmu-ag5** | PX548945 | *Neopestalotiopsis* sp. strain ZHKUCC 23-0440 | 100% | Pestalotiopsidaceae | Amphisphaeriales |
| **Cmu-ag6** | PX548946 | *Rhizopus oryzae* isolate N-30-2 | 100% | Rhizopodaceae | Mucorales |
| **Cmu-ag7** | PX548947 | *Neopestalotiopsis* sp. isolate FX26 | 100% | Pestalotiopsidaceae | Amphisphaeriales |
| **Cmu-ag8** | PX548948 | *Rhizopus delemar* isolate RS4-S2-05 | 99.83% | Rhizopodaceae | Mucorales |
| **Cmu-ag9** | PX548949 | *Neopestalotiopsis* sp. isolate FX27 | 100% | Pestalotiopsidaceae | Amphisphaeriales |
| **Cmu-ag10** | PX548950 | *Fusarium equiseti* isolate PAK-4 | 100% | Nectriaceae | Hypocreales |
| **Cmu-ag11** | PX548951 | *Curvularia sorghina* strain SFC20220920-G189 | 100% | Pleosporaceae | Pleosporales |
| **Cmu-ag12** | PX548952 | *Neopestalotiopsis* sp. isolate MFTU41 | 100% | Pestalotiopsidaceae | Amphisphaeriales |
| **Cmu-ag13** | PX548953 | *Curvularia suttoniae* strain M1349 | 100% | Pleosporaceae | Pleosporales |
| **Cmu-ag14** | PX548954 | *Neopestalotiopsis eucalypticola* CBS 264.37 | 100% | Pestalotiopsidaceae | Amphisphaeriales |
| **Cmu-ag15** | PX548955 | *Trichoderma virens* isolate SN4 | 100% | Hypocreaceae | Hypocreales |
| **Cmu-ag16** | PX548956 | *Gongronella* sp. strain CBI-S023 | 99.69% | Cunninghamellaceae | Mucorales |
| **Cmu-ag17** | PX548957 | *Trichosporon asahii* isolate KU60020.197 | 100% | Trichosporonaceae | Trichosporonales |
| **Cmu-ag18** | PX548958 | *Aspergillus japonicus* isolate PNNW_047 | 99.81% | Aspergillaceae | Eurotiales |
| **Cmu-ag19** | PX548959 | *Fusarium oxysporum* isolate B-3 | 99.43% | Nectriaceae | Hypocreales |
| **Cmu-ag20** | PX548960 | *Aspergillus tamarii* isolate Fungi_29 | 100% | Aspergillaceae | Eurotiales |
| **Cmu-ag21** | PX548961 | *Scedosporium boydii* CBS:127828 | 100% | Microascaceae | Microascales |
| **Cmu-ag22** | PX548962 | *Cunninghamella blakesleeana* CBS 133.27 | 100% | Cunninghamellaceae | Mucorales |
| **Cmu-ag23** | PX548963 | *Trichoderma* sp. voucher 47S | 100% | Hypocreaceae | Hypocreales |
| **Cmu-ag24** | PX548964 | *Cunninghamella* sp. strain XG09702-9-1 | 99% | Cunninghamellaceae | Mucorales |
| **Cmu-ag25** | PX548965 | *Cunninghamella* sp. strain XG09702-9-1 | 99% | Cunninghamellaceae | Mucorales |
| **Cmu-ag26** | PX548966 | *Cunninghamella* sp. strain XG09702-9-1 | 99% | Cunninghamellaceae | Mucorales |
| **Cmu-ag27** | PX548967 | *Lichtheimia* sp. isolate 1Oc3 | 100% | Lichtheimiaceae | Mucorales |
| **Cmu-ag28** | PX548968 | *Cunninghamella* sp. strain XG09702-9-1 | 100% | Cunninghamellaceae | Mucorales |
| **Cmu-ag29** | PX548969 | *Cunninghamella* sp. strain XG09702-9-1 | 99.25% | Cunninghamellaceae | Mucorales |
| **Cmu-ag30** | PX548970 | *Cunninghamella* sp. strain XG09702-9-1 | 100% | Cunninghamellaceae | Mucorales |
| **Cmu-ag31** | PX548971 | *Mucor irregularis* isolate BMU02257 | 99.81% | Mucoraceae | Mucorales |
| **Cmu-ag32** | PX548972 | *Cunninghamella* sp. strain XG09702-9-1 | 100% | Cunninghamellaceae | Mucorales |
| **Cmu-**bg1 | PX548973 | *Curvularia verruculosa* strain ALE-173 | 100% | Pleosporaceae | Pleosporales |
| **Cmu-**bg2 | PX548974 | *Trichoderma erinaceum* strain CIB T72 | 99.82% | Hypocreaceae | Hypocreales |
| **Cmu-**bg3 | PX548975 | *Trichoderma* sp. isolate yi1009_1 | 100% | Hypocreaceae | Hypocreales |
| **Cmu-**bg4 | PX548976 | *Cunninghamella blakesleeana* CBS 433.84 | 100% | Cunninghamellaceae | Mucorales |
| **Cmu-**bg5 | PX548977 | *Fusarium solani* isolate Shuhb-5 | 100% | Nectriaceae | Hypocreales |
| **Cmu-**bg6 | PX548978 | *Coprinellus* sp. isolate 1In3 | 100% | Psathyrellaceae | Agaricales |
| **Cmu-**bg7 | PX548979 | *Cunninghamella* sp. strain XG09702-9-1 | 100% | Cunninghamellaceae | Mucorales |
| **Cmu-**bg8 | PX548980 | *Fusarium solani* isolate XX-1 | 100% | Nectriaceae | Hypocreales |
| **Cmu-**bg9 | PX548981 | *Trichoderma tomentosum* strain DAOM 195050 | 100% | Hypocreaceae | Hypocreales |
| **Cmu-**bg10 | PX548982 | *Meyerozyma guilliermondii* isolate EGZ-34 | 100% | Debaryomycetaceae | Saccharomycetales |
| **Cmu-**bg11 | PX548983 | *Phanerochaete* sp. T172A | 99.83% | Phanerochaetaceae | Polyporales |
| **Cmu-**bg12 | PX548984 | *Aspergillus nomiae* isolate USPMTOX123 | 100% | Aspergillaceae | Eurotiales |
| **Cmu-**bg13 | PX548985 | *Trichoderma* lixii culture CCTCC:AW506 | 100% | Hypocreaceae | Hypocreales |
| **Cmu-**bg14 | PX548986 | *Trichoderma* sp. isolate CU51 | 100% | Hypocreaceae | Hypocreales |
| **Cmu-**bg15 | PX548987 | *Aspergillus* sp. strain SH1 | 99.82% | Aspergillaceae | Eurotiales |
| **Cmu-**bg16 | PX548988 | *Cunninghamella* sp. strain XG09702-9-1 | 100% | Cunninghamellaceae | Mucorales |
| **Cmu-**bg17 | PX548989 | *Trichoderma gamsii* strain DAOM 234229 | 100% | Hypocreaceae | Hypocreales |
| **Cmu-**bg18 | PX548990 | *Trichoderma* sp. isolate P0-058 | 100% | Hypocreaceae | Hypocreales |
| **Cmu-**bg19 | PX548991 | *Lasiodiplodia theobromae* isolate MBAi184AG | 100% | Botryosphaeriaceae | Botryosphaeriales |
| **Cmu-**bg20 | PX548992 | *Chaetomium globosum* BCC 31359 | 100% | Chaetomiaceae | Sordariales |
| **Cmu-**bg21 | PX548993 | *Scedosporium dehoogii* isolate SUN587 | 100% | Microascaceae | Microascales |
| **Cmu-**bg22 | PX548994 | *Cladosporium halotolerans* strain KMM 4398 | 100% | Cladosporiaceae | Cladosporiales |
| **Cmu-**bg23 | PX548995 | *Epicoccum* sp. strain JME-7 | 100% | Didymellaceae | Pleosporales |
| **Cmu-**bg24 | PX548996 | *Gilbertella persicaria* CBS:325.71A | 99.67% | Choanephoraceae | Mucorales |
| **Cmu-**bg25 | PX548997 | *Curvularia alcornii* strain M1438 | 100% | Pleosporaceae | Pleosporales |
| **Cmu-**bg26 | PX548998 | *Penicillium soli* KUMCC 18-0202 | 100% | Aspergillaceae | Eurotiales |
| **Cmu-**bg27 | PX548999 | *Trichoderma* sp. F0210-12L2 | 100% | Hypocreaceae | Hypocreales |
| **Cmu-**bg28 | PX549000 | *Trichoderma tomentosum* isolate PAN12-53 | 99% | Hypocreaceae | Hypocreales |
| **Cmu-**bg29 | PX549001 | *Curvularia verruculosa* strain M0239 | 100% | Pleosporaceae | Pleosporales |
| **Cmu-**bg30 | PX549002 | *Trichoderma atroviride* strain Fe57K | 100% | Hypocreaceae | Hypocreales |
| **Cmu-cg1** | PX549003 | *Curvularia lunata* isolate BOT17 | 100% | Pleosporaceae | Pleosporales |
| **Cmu-cg2** | PX549004 | *Rhizopus stolonifer* isolate KG | 100% | Rhizopodaceae | Mucorales |
| **Cmu-cg3** | PX549005 | *Hypocrea lixii* strain SHMH1012 | 100% | Hypocreaceae | Hypocreales |
| **Cmu-cg4** | PX549006 | *Cunninghamella* sp. strain XG09702-9-1 | 99.21% | Cunninghamellaceae | Mucorales |
| **Cmu-cg5** | PX549007 | *Cunninghamella* sp. strain XG09702-9-1 | 99.22% | Cunninghamellaceae | Mucorales |
| **Cmu-cg6** | PX549008 | *Rhizopus stolonifer* isolate KN | 100% | Rhizopodaceae | Mucorales |
| **Cmu-cg7** | PX549009 | *Cunninghamella* sp. strain XG09702-9-1 | 99.22% | Cunninghamellaceae | Mucorales |
| **Cmu-cg8** | PX549010 | *Mucor* sp. isolate YY47 | 99.81% | Mucoraceae | Mucorales |
| **Cmu-cg9** | PX549011 | *Aspergillus niger* isolate RDE293 | 100% | Aspergillaceae | Eurotiales |
| **Cmu-cg10** | PX549012 | *Aspergillus niger* isolate 93_T7 | 100% | Aspergillaceae | Eurotiales |
| **Cmu-cg11** | PX549013 | *Lichtheimia* sp. isolate 1Oc3 | 99.61% | Lichtheimiaceae | Mucorales |
| **Cmu-cg12** | PX549014 | *Scedosporium apiospermum* CBS:127619 | 99.83% | Microascaceae | Microascales |
| **Cmu-cg13** | PX549015 | *Chaetomium globosum* strain UOA/HCPF 9215 | 100% | Chaetomiaceae | Sordariales |
| **Cmu-cg14** | PX549016 | *Trichoderma lixii* culture CCTCC:AO422 | 100% | Hypocreaceae | Hypocreales |
| **Cmu-cg15** | PX549017 | *Lichtheimia brasiliensis* strain URM6911 | 100% | Lichtheimiaceae | Mucorales |
| **Cmu-cg16** | PX549018 | *Trichoderma* sp. isolate SDAS203873 | 100% | Hypocreaceae | Hypocreales |
| **Cmu-cg17** | PX549019 | *Fusarium proliferatum* isolate 14F | 100% | Nectriaceae | Hypocreales |
| **Cmu-cg18** | PX549020 | *Curvularia lunata* isolate JUF0079 | 100% | Pleosporaceae | Pleosporales |
| **Cmu-cg19** | PX549021 | *Mortierella wolfii* strain DTO 403-F4 | 99.70% | Mortierellaceae | Mortierellales |
| **Cmu-cg20** | PX549022 | *Trichoderma virens* isolate 5L | 100% | Hypocreaceae | Hypocreales |
| **Cmu-cg21** | PX549023 | *Lichtheimia* sp. strain XG16000-9-1 | 100% | Lichtheimiaceae | Mucorales |
| **Cmu-cg22** | PX549024 | *Trichoderma lixii* culture CCTCC:AO422 | 100% | Hypocreaceae | Hypocreales |
| **Cmu-cg23** | PX549025 | *Gilbertella persicaria* isolate ARM 1171 | 99.83% | Choanephoraceae | Mucorales |
| **Cmu-cg24** | PX549026 | *Gilbertella persicaria* isolate ARM 1171 | 99.83% | Choanephoraceae | Mucorales |
| **Cmu-cg25** | PX549027 | *Gilbertella persicaria* CBS:325.71A | 99.67% | Choanephoraceae | Mucorales |
| **Cmu-cg26** | PX549028 | *Gilbertella persicaria* CBS:325.71A | 99.67% | Choanephoraceae | Mucorales |
| **Cmu-cg27** | PX549029 | *Westerdykella globosa* isolate N1 s | 99.59% | Sporormiaceae | Pleosporales |
| **Cmu-cg28** | PX549030 | *Curvularia* sp. isolate UgN58 | 99.51% | Pleosporaceae | Pleosporales |
| **Cmu-cg29** | PX549031 | *Trichoderma erinaceum* strain CIB T72 | 100% | Hypocreaceae | Hypocreales |
| **Cmu-cg30** | PX549032 | *Lichtheimia* sp. isolate 1Oc3 | 99.61% | Lichtheimiaceae | Mucorales |
| **Cmu-cg31** | PX549033 | *Irpex laceratus* isolate EL/TP/UK/01/2020 | 100% | Irpicaceae | Polyporales |
| **Cmu-cg32** | PX549034 | *Trichoderma virens* isolate ACP-Tv13 | 100% | Hypocreaceae | Hypocreales |
| **Cmu-cg33** | PX549035 | *Gilbertella persicaria* isolate ARM 1171 | 100% | Choanephoraceae | Mucorales |
| **Cmu-cg34** | PX549036 | *Cercospora* cf. malloti CPC 20737 | 99% | Mycosphaerellaceae | Mycosphaerellales |
| **Cmu-cg35** | PX549037 | *Curvularia* sp. strain M1380 | 100% | Pleosporaceae | Pleosporales |
| **Cmu-cg36** | PX549038 | *Trichoderma virens* isolate ACP-Tv13 | 100% | Hypocreaceae | Hypocreales |
| **Cmu-cg37** | PX549039 | *Lichtheimia* sp. isolate 1TW9 | 100% | Lichtheimiaceae | Mucorales |
| **Cmu-cg38** | PX549040 | *Talaromyces pinophilus* isolate Glinf047 | 100% | Aspergillaceae | Eurotiales |

**Table S7.** Species names, strain numbers, and corresponding GenBank accession numbers of the *Xylaria* taxa (TE) were used in the phylogenetic analyses.The newly generated sequences were labeled as CX for *Odontotermes* sp. (China) and CMU for *Macrotermes gilvus* (Thailand), type strains are shown in bold, and “/” represents the unavailable sequences.

| **Taxon** | **Origin** | **Specimens** | **Habitat** | **ITS** | **ACT** | **RPB2** | **TUB2** |
| --- | --- | --- | --- | --- | --- | --- | --- |
| *Xylaria fimbriata* | France | HAST 491 | Termite nests | GU324753 | GQ853040 | GQ853022 | GQ502705 |
| ***Xylaria ripicola*** | **South Korea** | **KA11-0060-1** | **/** | **NR153251** | **/** | **KU554696** | **/** |
| *Xylaria ripicola* | South Korea | KA11-0060-2 | / | KM817200 | / | KU554697 | / |
| *Xylaria brevifurcata* | Taiwan | YMJ986 | on the ground of a mango orchard | OQ845481 | OQ818371 | OQ851525 | OQ818431 |
| ***Xylaria brevifurcata*** | **Taiwan** | **YMJ1381** | **on the ground of a mango orchard** | **OQ845482** | **OQ818372** | **OQ851526** | **OQ818432** |
| ***Xylaria tenellifurcata*** | **Taiwan** | **YMJ1070** | **ground of a bamboo plantation** | **OQ845498** | **OQ818388** | **OQ851542** | **OQ818448** |
| ***Xylaria scoparia*** | **Taiwan** | **YMJ1435** | **ground of a bamboo plantation** | **OQ843014** | **OQ818429** | **OQ851583** | **OQ818489** |
| *Xylaria scoparia* | Taiwan | YMJ1428 | on ground | OQ843013 | OQ818428 | OQ851582 | OQ818488 |
| ***Xylaria insignifurcata*** | **Taiwan** | **YMJ650** | **ground of a bamboo plantation** | **GU324758** | **GQ853051** | **GQ853033** | **GQ502716** |
| *Xylaria insignifurcata* | Taiwan | YMJ1198 | ground of a bamboo plantation | OQ845497 | OQ818387 | OQ851541 | OQ818447 |
| ***Xylaria hoehnelii*** | **Taiwan** | **YMJ643** | **ground of a mango orchard** | **OQ845489** | **OQ818379** | **OQ851533** | **OQ818439** |
| *Xylaria hoehnelii* | Taiwan | YMJ1159 | ground of a mango orchard | OQ845493 | OQ818383 | OQ851537 | OQ818443 |
| ***Xylaria termiticola*** | **Japan** | **NBRC102095** | **Termite nests** | **AB274813** | **PQ605196** | **PQ605191** | **PQ605186** |
| ***Xylaria ischnostroma*** | **Thailand** | **SWUF18-22.1** | **Termite nests** | **MT622788** | **MW459225** | **/** | **MW459244** |
| ***Xylaria furcatula*** | **Taiwan** | **YMJ1103** | **ground of a bamboo plantation** | **OQ845484** | **OQ818374** | **OQ851528** | **OQ818434** |
| *Xylaria furcatula* | Taiwan | YMJ1099 | ground of a bamboo plantation | OQ845483 | OQ818373 | OQ851527 | OQ818433 |
| *Xylaria conica* | Thailand | SWUF18-4.3 | Termite nests | MT622786 | MW459223 | / | / |
| ***Xylaria conica*** | **Thailand** | **SWUF18-4.4** | **Termite nests** | **MT622787** | **MW459224** | **/** | **MW459243** |
| *Xylaria tentaculata* | Korea | KA13-1324 | Leaf litter or wood | KM077163 | / | / | / |
| *Xylaria tentaculata* | Korea | KA13-1325 | Leaf litter or wood | KM077164 | / | / | / |
| ***Xylaria coprinicola*** | **China** | **YMJ 1145** | **Termite nests** | **HM585020** | **HM585017** | **HM585019** | **HM585018** |
| *Xylaria vinacea* | Thailand | SWUF 182.1 | Termite nests | MT622781 | MW459219 | / | MW459239 |
| ***Xylaria vinacea*** | **Thailand** | **SWUF18-2.10** | **Termite nests** | **MT622783** | **MW459220** | **/** | **MW459240** |
| ***Xylaria brunneovinosa*** | **Taiwan** | **HAST 720** | **Termite nests** | **EU179862** | **GQ853041** | **GQ853023** | **GQ502706** |
| ***Xylaria minima*** | **Thailand** | **SWUF18-3.2** | **Termite nests** | **MT622789** | **MW459226** | **/** | **MW459245** |
| ***Xylaria terricola*** | **Taiwan** | **YMJ 1375** | **Termite nests** | **MF577042** | **MF577045** | **MF577043** | **MF577044** |
| ***Xylaria theinhirunae*** | **Thailand** | **SWUF17-44.1** | Termite nests | **MT622771** | **OQ845432** | **OQ851592** | **OQ845440** |
| *Xylaria theinhirunae* | Thailand | SWUF16-10.1 | Termite nests | MT622773 | OQ845431 | OQ851591 | OQ845439 |
| *Xylaria guepini* | France | HD 20120101 | on humiferous sandy soil | OP863307 | OP856890 | OP856891 | OP856892 |
| ***Xylaria fulvescens*** | **Thailand** | **SWUF17-27.2** | **Termite nests** | **MT622780** | **MW459218** | **/** | **MW459238** |
| *Xylaria thienhirunae* | Thailand | SWUF16-6.2 | Termite nests | MT622770 | MW459210 | / | MW459235 |
| *Xylaria chaiyaphumensis* | Thailand | SWUF 16-11.4 | Soil near a termite nest | MT622776 | MW459213 | / | MW459236 |
| *Xylaria hoehnelii* | Taiwan | HAST 642 | ground of a mango orchard | GU324759 | GQ853054 | GQ853036 | GQ502719 |
| ***Xylaria intraflava*** | **Taiwan** | **HAST 725** | **Termite nests** | **EU179866** | **GQ853053** | **GQ853035** | **GQ502718** |
| *Xylaria* sp. | Togo | AAG5 | *Ancistrotermes cavithorax* | EU164400 | / | / | / |
| *Xylaria subintraflava* | Thailand | SWUF 1722.2 | Termite nests | MT622764 | MW459206 | / | MW459231 |
| *Xylaria subintraflava* | Thailand | SWUF 1724.2 | Termite nests | MT622757 | MW459207 | / | MW459232 |
| ***Xylaria reinkingii var. microspora*** | **Thailand** | **SWUF17-19.1** | **Termite nests** | **MT622769** | **MW459209** | **/** | **MW459234** |
| ***Xylaria griseosepiacea*** | **Taiwan** | **HAST 641** | **Termite nests** | **EU179865** | **GQ853049** | **GQ853031** | **GQ502714** |
| ***Xylaria atrodivaricata*** | **Taiwan** | **HAST 95052001** | **Termite nests** | **EU178739** | **GQ853048** | **GQ853030** | **GQ502713** |
| ***Xylaria insolita*** | **Taiwan** | **HAST 990903011251** | **Termite nests** | **MN655979** | **MN656985** | **MN656981** | **MN656983** |
| ***Xylaria ochraceostroma*** | **Taiwan** | **HAST 401** | **ground of a mango orchard** | **EU179869** | **GQ853052** | **GQ853034** | **GQ502717** |
| *Xylaria furcata* | Taiwan | HAST 646 | Termite nests | GU324757 | GQ853050 | GQ853032 | GQ502715 |
| *Xylaria furcata* | Taiwan | YMJ956 | Termite nests | OQ842973 | OQ818389 | OQ851543 | OQ818449 |
| ***Xylaria siamensis*** | **Thailand** | **SWUF17-20.2** | **Termite nests** | **MT622765** | **MW459208** | **/** | **MW459233** |
| ***Xylaria acuminatilongissima*** | **Taiwan** | **HAST 623** | **Termite nests** | **EU178738** | **GQ853046** | **GQ853028** | **GQ502711** |
| ***Xylaria neonigripes*** | **Taiwan** | **HAST145669** | **Termite nests/on the ground of a bamboo plantation** | **MZ888988** | **MZ901349** | **MZ901335** | **MZ901321** |
| *Xylaria neonigripes* | China | WLS 2030 | Termite nests/on the ground of a bamboo plantation | MZ888987 | MZ901348 | MZ901334 | MZ901320 |
| ***Xylaria margaretae*** | **Thailand** | **SWUF17-34.1** | **Termite nests** | **MT622778** | **MW459216** | **/** | **/** |
| *Xylaria margaretae* | Thailand | SWUF 1734.2 | Termite nests | MT622779 | MW459217 | / | / |
| ***Xylaria cirrata*** | **Taiwan** | **HAST 664** | **Termite nests** | **EU179863** | **GQ853042** | **GQ853024** | **GQ502707** |
| ***Xylaria rogersionigripes*** | **Taiwan** | **HAST145684** | **Ground of a bamboo plantation** | **MZ888991** | **MZ901352** | **MZ901338** | **MZ901324** |
| *Xylaria rogersionigripes* | China | WLS 2064 | Termite nests | MZ888990 | MZ901351 | MZ901337 | MZ901323 |
| *Xylaria cf. nigripes* | Thailand | SWUF 1731.2 | Termite nests | MT622790 | MW459202 | / | MW459229 |
| *Xylaria cf. nigripes* | Thailand | SWUF 1736.1 | Termite nests | MT622791 | MW459203 | / | / |
| *Xylaria nigripes* | China | WLS2056 | Termite nests | MZ888989 | MZ901350 | MZ901336 | MZ901322 |
| *Xylaria nigripes* | Taiwan, China | YMJ653 | ground of a mango orchard | EU179868 | GQ853045 | GQ853027 | GQ502710 |
| ***Xylaria mianyangensis*** | **China** | **HAST 145687** | **On ground** | **MZ888986** | **MZ901347** | **MZ901333** | **MZ901319** |
| *Xylaria mianyangensis* | China | WLS 2062 | On ground | MZ888993 | MZ901354 | MZ901340 | MZ901326 |
| *Xylaria mianyangensis* | China | WLS 2069 | On ground | MZ888994 | MZ901355 | MZ901341 | MZ901327 |
| *Xylaria mianyangensis* | China | WLS2077 | On ground | MZ888995 | MZ901356 | MZ901342 | MZ901328 |
| ***Xylaria subescharoidea*** | **Taiwan** | **HAST 990604011188** | **Termite nests** | **MN655980** | **MN656986** | **MN656982** | **MN656984** |
| *Xylaria subescharoidea* | China | WLS 2026 | Termite nests | MZ888999 | MZ901360 | MZ901346 | MZ901332 |
| *Xylaria subescharoidea* | China | YMJ 1442 | Termite nests | MZ888997 | MZ901358 | MZ901344 | MZ901330 |
| ***Xylaria escharoidea*** | **Taiwan** | **HAST 658** | **Termite nests** | **EU179864** | **GQ853044** | **GQ853026** | **GQ502709** |
| *Xylaria cf. escharoidea* | Thailand | SWUF1735.1 | Termite nests | MT622792 | MW459200 | / | MW459227 |
| *Xylaria cf. escharoidea* | Thailand | SWUF 1739.1 | Termite nests | MT622794 | MW459201 | / | MW459228 |
| *Xylaria neonigripes* | Taiwan | HAST 722 | Termite nests | GU324756 | GQ853047 | GQ853029 | GQ502712 |
| ***Xylaria subintraflava*** | **Thailand** | **SWUF16-4.3** | **Termite nests** | **MT622762** | **MW459204** | **/** | **MW459230** |
| ***Xylaria sihanonthii*** | **Thailand** | **SWUF18-1.3** | **Termite nests** | **MT622785** | **MW459222** | **/** | **MW459242** |
| *Xylaria sihanonthii* | Thailand | SWUF18-5.1 | Termite nests | MT622784 | MW459221 | / | MW459241 |
| ***Xylaria iriomotensis*** | **Japan** | **NBRC 33288** | **Incubated termite nests** | **AB274815** | **PQ605195** | **PQ605190** | **PQ605185** |
| *Xylaria polymorpha* | USA | JDR 1012 | Wood/stump | GU322460 | GQ452364 | GQ848343 | GQ495954 |
| *Xylaria venustula* | Taiwan | HAST 88113002 | On bark | GU300091 | GQ421287 | GQ844807 | GQ487699 |
| *Xylaria angulosa* | Taiwan | HAST146296 | On ground | PQ582212 | PQ605193 | PQ605188 | PQ605183 |
| *Xylaria angulosa* | Taiwan | HAST146295 | On ground | PQ582213 | PQ605194 | PQ605189 | PQ605184 |
| ***Xylaria* cf*. escharoidea*** | **China** | **CX0/GMBCC1406** | ***Odontotermes* termite nests** | **PV679728** | **PV683221** | **PV683167** | **PV683140** |
| ***Xylaria* var. *microspora*** | **China** | **CX1/GMBCC1407** | ***Odontotermes* termite nests** | **PV679729** | **PV683080** | **PV683172** | **PV683145** |
| ***Xylaria* sp.** | **China** | **CX2/GMBCC1408** | ***Odontotermes* termite nests** | **PV679730** | **PV683086** | **PV683178** | **PV683119** |
| ***Xylaria* var. *microspora*** | **China** | **CX3/GMBCC1409** | ***Odontotermes* termite nests** | **PV679731** | **PV683081** | **PV683173** | **PV683146** |
| ***Xylaria ochraceostroma*** | **China** | **CX4/GMBCC1410** | ***Odontotermes* termite nests** | **PV679732** | **PV683107** | **PV683209** | **PV683161** |
| ***Xylaria* cf*. escharoidea*** | **China** | **CX5/GMBCC1411** | ***Odontotermes* termite nests** | **PV679733** | **PV683222** | **PV683168** | **PV683141** |
| ***Xylaria* sp.** | **China** | **CX6/GMBCC1412** | ***Odontotermes* termite nests** | **PV679734** | **PV683087** | **PV683179** | **PV683120** |
| ***Xylaria* var. *microspora*** | **China** | **CX7/GMBCC1413** | ***Odontotermes* termite nests** | **PV679735** | **PV683082** | **PV683174** | **PV683147** |
| ***Xylaria rogersionigripes*** | **China** | **CX8/GMBCC1414** | ***Odontotermes* termite nests** | **PV679736** | **PV683078** | **PV683211** | **/** |
| ***Xylaria iriomotensis*** | **China** | **CX9/GMBCC1415** | ***Odontotermes* termite nests** | **PV679737** | **PV683109** | **PV683199** | **PV683151** |
| ***Xylaria* sp.** | **China** | **CX10/GMBCC1416** | ***Odontotermes* termite nests** | **PV679738** | **PV683088** | **PV683180** | **PV683121** |
| ***Xylaria iriomotensis*** | **China** | **CX11/GMBCC1417** | ***Odontotermes* termite nests** | **PV679739** | **PV683110** | **PV683200** | **PV683152** |
| ***Xylaria* sp.** | **China** | **CX12/GMBCC1418** | ***Odontotermes* termite nests** | **PV679740** | **PV683089** | **PV683181** | **PV683122** |
| ***Xylaria* sp.** | **China** | **CX13/GMBCC1419** | ***Odontotermes* termite nests** | **PV679741** | **PV683090** | **PV683182** | **PV683123** |
| ***Xylaria* cf*. escharoidea*** | **China** | **CX14/GMBCC1420** | ***Odontotermes* termite nests** | **PV679742** | **PV683223** | **PV683169** | **PV683142** |
| ***Xylaria* sp.** | **China** | **CX15/GMBCC1421** | ***Odontotermes* termite nests** | **PV679743** | **PV683091** | **PV683183** | **PV683124** |
| ***Xylaria iriomotensis*** | **China** | **CX16/GMBCC1422** | ***Odontotermes* termite nests** | **PV679744** | **PV683111** | **PV683201** | **PV683153** |
| ***Xylaria iriomotensis*** | **China** | **CX17/GMBCC1423** | ***Odontotermes* termite nests** | **PV679745** | **PV683112** | **PV683202** | **PV683154** |
| ***Xylaria* sp.** | **China** | **CX18/GMBCC1424** | ***Odontotermes* termite nests** | **PV679746** | **PV683092** | **PV683184** | **PV683125** |
| ***Xylaria* sp.** | **China** | **CX19/GMBCC1425** | ***Odontotermes* termite nests** | **PV679747** | **PV683093** | **PV683185** | **PV683126** |
| ***Xylaria* sp.** | **China** | **CX20/GMBCC1426** | ***Odontotermes* termite nests** | **PV679748** | **PV683215** | **PV683213** | **PV683163** |
| ***Xylaria iriomotensis*** | **China** | **CX21/GMBCC1427** | ***Odontotermes* termite nests** | **PV679749** | **PV683113** | **PV683203** | **PV683155** |
| ***Xylaria iriomotensis*** | **China** | **CX22/GMBCC1428** | ***Odontotermes* termite nests** | **PV679750** | **PV683114** | **PV683204** | **PV683156** |
| ***Xylaria iriomotensis*** | **China** | **CX23/GMBCC1429** | ***Odontotermes* termite nests** | **PV679751** | **PV683115** | **PV683205** | **PV683157** |
| ***Xylaria* sp.** | **China** | **CX24/GMBCC1430** | ***Odontotermes* termite nests** | **PV679752** | **PV683094** | **PV683186** | **PV683127** |
| ***Xylaria* sp.** | **China** | **CX25/GMBCC1431** | ***Odontotermes* termite nests** | **PV679753** | **PV683095** | **PV683187** | **PV683128** |
| ***Xylaria* var. *microspora*** | **China** | **CX26/GMBCC1432** | ***Odontotermes* termite nests** | **PV679754** | **PV683083** | **PV683175** | **PV683148** |
| ***Xylaria* sp.** | **China** | **CX28/GMBCC1434** | ***Odontotermes* termite nests** | **PV679755** | **PV683096** | **PV683188** | **PV683129** |
| ***Xylaria* sp.** | **China** | **CX29/GMBCC1435** | ***Odontotermes* termite nests** | **PV679756** | **PV683217** | **/** | **PV683165** |
| ***Xylaria* sp.** | **China** | **CX30/GMBCC1436** | ***Odontotermes* termite nests** | **PV679757** | **PV683218** | **/** | **PV683166** |
| ***Xylaria* cf*. escharoidea*** | **China** | **CXA/GMBCC1637** | ***Odontotermes* termite nests** | **PV679758** | **PV683219** | **PV683170** | **PV683143** |
| ***Xylaria* var. *microspora*** | **China** | **CXB/GMBCC1638** | ***Odontotermes* termite nests** | **PV679759** | **PV683084** | **PV683176** | **PV683149** |
| ***Xylaria iriomotensis*** | **China** | **CXC/GMBCC1639** | ***Odontotermes* termite nests** | **PV679760** | **PV683116** | **PV683206** | **PV683158** |
| ***Xylaria* sp.** | **China** | **CXD/GMBCC1640** | ***Odontotermes* termite nests** | **PV679761** | **PV683097** | **PV683189** | **PV683130** |
| ***Xylaria ochraceostroma*** | **China** | **CXE/GMBCC1641** | ***Odontotermes* termite nests** | **PV679762** | **PV683108** | **PV683210** | **PV683162** |
| ***Xylaria* sp.** | **China** | **CXF/GMBCC1642** | ***Odontotermes* termite nests** | **PV679763** | **PV683098** | **PV683190** | **PV683131** |
| ***Xylaria iriomotensis*** | **China** | **CXG/GMBCC1643** | ***Odontotermes* termite nests** | **PV679764** | **PV683117** | **PV683207** | **PV683159** |
| ***Xylaria* sp.** | **China** | **CXH/GMBCC1644** | ***Odontotermes* termite nests** | **PV679765** | **PV683099** | **PV683191** | **PV683132** |
| ***Xylaria* sp.** | **China** | **CXI/GMBCC1653** | ***Odontotermes* termite nests** | **PV679766** | **PV683100** | **PV683192** | **PV683133** |
| ***Xylaria* cf*. escharoidea*** | **China** | **CXJ/GMBCC1654** | ***Odontotermes* termite nests** | **PV679767** | **PV683220** | **PV683171** | **PV683144** |
| ***Xylaria* sp.** | **China** | **CXK/GMBCC1645** | ***Odontotermes* termite nests** | **PV679768** | **PV683101** | **PV683193** | **PV683134** |
| ***Xylaria* sp.** | **China** | **CXL/GMBCC1433** | ***Odontotermes* termite nests** | **PV679769** | **PV683102** | **PV683194** | **PV683135** |
| ***Xylaria* sp.** | **China** | **CXM/GMBCC1646** | ***Odontotermes* termite nests** | **PV679770** | **PV683103** | **PV683195** | **PV683136** |
| ***Xylaria* sp.** | **China** | **CXN/GMBCC1647** | ***Odontotermes* termite nests** | **PV679771** | **PV683104** | **PV683196** | **PV683137** |
| ***Xylaria* sp.** | **China** | **CXO/GMBCC1655** | ***Odontotermes* termite nests** | **PV679772** | **PV683105** | **PV683197** | **PV683138** |
| ***Xylaria* var. *microspora*** | **China** | **CXP/GMBCC1648** | ***Odontotermes* termite nests** | **PV679773** | **PV683085** | **PV683177** | **PV683150** |
| ***Xylaria rogersionigripes*** | **China** | **CXQ/GMBCC1649** | ***Odontotermes* termite nests** | **PV679774** | **PV683079** | **PV683212** | **/** |
| **Xylaria sp.** | **China** | **CXR/GMBCC1650** | ***Odontotermes* termite nests** | **PV679775** | **PV683106** | **PV683198** | **PV683139** |
| ***Xylaria iriomotensis*** | **China** | **CXS/GMBCC1651** | ***Odontotermes* termite nests** | **PV679776** | **PV683118** | **PV683208** | **PV683160** |
| ***Xylaria* sp.** | **China** | **CXT/GMBCC1652** | ***Odontotermes* termite nests** | **PV679777** | **PV683216** | **PV683214** | **PV683164** |
| ***Xylaria ochraceostroma*** | **Thailand** | **CMU-X2** | *Macrotermes gilvus* | **PX517510** | **PX548729** | **PX607997** | **PX608043** |
| ***Xylaria ochraceostroma*** | **Thailand** | **CMU-X8** | ***Macrotermes gilvus*** | **PX517511** | **PX548730** | **PX607998** | **PX608044** |
| ***Xylaria ochraceostroma*** | **Thailand** | **CMU-X13** | ***Macrotermes gilvus*** | **PX517512** | **PX548731** | **PX607999** | **PX608045** |
| ***Xylaria ochraceostroma*** | **Thailand** | **CMU-X14** | ***Macrotermes gilvus*** | **PX517513** | **PX548732** | **PX608000** | **PX608046** |
| ***Xylaria ochraceostroma*** | **Thailand** | **CMU-X20** | ***Macrotermes gilvus*** | **PX517514** | **PX548733** | **PX608001** | **PX608047** |
| ***Xylaria ochraceostroma*** | **Thailand** | **CMU-X17** | ***Macrotermes gilvus*** | **PX517515** | **PX548734** | **PX608002** | **PX608048** |
| ***Xylaria ochraceostroma*** | **Thailand** | **CMU-X15** | ***Macrotermes gilvus*** | **PX517516** | **PX548735** | **PX608003** | **PX608049** |
| ***Xylaria ochraceostroma*** | **Thailand** | **CMU-X22** | ***Macrotermes gilvus*** | **PX517517** | **PX548736** | **PX608004** | **PX608050** |
| ***Xylaria ochraceostroma*** | **Thailand** | **CMU-X24** | ***Macrotermes gilvus*** | **PX517518** | **PX548737** | **PX608005** | **PX608051** |
| ***Xylaria ochraceostroma*** | **Thailand** | **CMU-A5** | ***Macrotermes gilvus*** | **PX517519** | **PX548738** | **PX608006** | **PX608052** |
| ***Xylaria ochraceostroma*** | **Thailand** | **CMU-A6** | ***Macrotermes gilvus*** | **PX517520** | **PX548739** | **PX608007** | **PX608053** |
| ***Xylaria ochraceostroma*** | **Thailand** | **CMU-a5** | ***Macrotermes gilvus*** | **PX517521** | **PX548740** | **PX608008** | **PX608054** |
| ***Xylaria ochraceostroma*** | **Thailand** | **CMU-a8** | ***Macrotermes gilvus*** | **PX517522** | **PX548741** | **PX608009** | **PX608055** |
| ***Xylaria ochraceostroma*** | **Thailand** | **CMU-a7** | ***Macrotermes gilvus*** | **PX517523** | **PX548742** | **PX608010** | **PX608056** |
| ***Xylaria ochraceostroma*** | **Thailand** | **CMU-a15** | ***Macrotermes gilvus*** | **PX517524** | **PX548743** | **PX608011** | **PX608057** |
| ***Xylaria ochraceostroma*** | **Thailand** | **CMU-A22** | ***Macrotermes gilvus*** | **PX517525** | **PX548744** | **PX608012** | **PX608058** |
| ***Xylaria minima*** | **Thailand** | **CMU-X31** | ***Macrotermes gilvus*** | **PX517526** | **PX548745** | **PX608013** | **PX608059** |
| ***Xylaria minima*** | **Thailand** | **CMU-X43** | ***Macrotermes gilvus*** | **PX517527** | **PX548746** | **PX608014** | **PX608060** |
| ***Xylaria minima*** | **Thailand** | **CMU-X47** | ***Macrotermes gilvus*** | **PX517528** | **PX548747** | **PX608015** | **PX608061** |
| ***Xylaria minima*** | **Thailand** | **CMU-A8** | ***Macrotermes gilvus*** | **PX517529** | **PX548748** | **PX608016** | **PX608062** |
| ***Xylaria minima*** | **Thailand** | **CMU-A12** | ***Macrotermes gilvus*** | **PX517530** | **PX548749** | **PX608017** | **PX608063** |
| ***Xylaria* sp.** | **Thailand** | **CMU-X7** | ***Macrotermes gilvus*** | **PX517531** | **PX548750** | **PX608018** | **PX608064** |
| ***Xylaria* sp.** | **Thailand** | **CMU-X19** | ***Macrotermes gilvus*** | **PX517532** | **PX548751** | **PX608019** | **PX608065** |
| ***Xylaria* sp.** | **Thailand** | **CMU-X23** | ***Macrotermes gilvus*** | **PX517533** | **PX548752** | **PX608020** | **PX608066** |
| ***Xylaria* sp.** | **Thailand** | **CMU-X25** | ***Macrotermes gilvus*** | **PX517534** | **PX548753** | **PX608021** | **PX608067** |
| ***Xylaria* sp.** | **Thailand** | **CMU-a3** | ***Macrotermes gilvus*** | **PX517535** | **PX548754** | **PX608022** | **PX608068** |
| ***Xylaria* sp.** | **Thailand** | **CMU-a14** | ***Macrotermes gilvus*** | **PX517536** | **PX548755** | **PX608023** | **PX608069** |
| ***Xylaria* sp.** | **Thailand** | **CMU-a17** | ***Macrotermes gilvus*** | **PX517537** | **PX548756** | **PX608024** | **PX608070** |
| ***Xylaria* sp.** | **Thailand** | **CMU-X40** | ***Macrotermes gilvus*** | **PX517538** | **PX548757** | **PX608025** | **PX608071** |
| ***Xylaria escharoidea*** | **Thailand** | **CMU-X3** | ***Macrotermes gilvus*** | **PX517539** | **PX548758** | **PX608026** | **PX608072** |
| ***Xylaria escharoidea*** | **Thailand** | **CMU-X26** | ***Macrotermes gilvus*** | **PX517540** | **PX548759** | **PX608027** | **PX608073** |
| ***Xylaria escharoidea*** | **Thailand** | **CMU-X18** | ***Macrotermes gilvus*** | **PX517541** | **PX548760** | **PX608028** | **PX608074** |
| ***Xylaria reinkingii var. microspora*** | **Thailand** | **CMU-X6** | ***Macrotermes gilvus*** | **PX517542** | **PX548761** | **PX608029** | **PX608075** |
| ***Xylaria reinkingii var. microspora*** | **Thailand** | **CMU-X29** | ***Macrotermes gilvus*** | **PX517543** | **PX548762** | **PX608030** | **PX608076** |
| ***Xylaria reinkingii var. microspora*** | **Thailand** | **CMU-X50** | ***Macrotermes gilvus*** | **PX517544** | **PX548763** | **PX608031** | **PX608077** |
| ***Xylaria reinkingii var. microspora*** | **Thailand** | **CMU-X28** | ***Macrotermes gilvus*** | **PX517545** | **PX548764** | **PX608032** | **PX608078** |
| ***Xylaria reinkingii var. microspora*** | **Thailand** | **CMU-A3** | ***Macrotermes gilvus*** | **PX517546** | **PX548765** | **PX608033** | **PX608079** |
| ***Xylaria reinkingii var. microspora*** | **Thailand** | **CMU-a16** | ***Macrotermes gilvus*** | **PX517547** | **PX548766** | **PX608034** | **PX608080** |
| ***Xylaria* sp.** | **Thailand** | **CMU-X4** | ***Macrotermes gilvus*** | **PX517548** | **PX548767** | **/** | **PX608081** |
| ***Xylaria* sp.** | **Thailand** | **CMU-X9** | ***Macrotermes gilvus*** | **PX517549** | **PX548768** | **/** | **PX608082** |
| ***Xylaria* sp.** | **Thailand** | **CMU-a18** | ***Macrotermes gilvus*** | **PX517550** | **PX548769** | **/** | **PX608083** |
| ***Xylaria* sp.** | **Thailand** | **CMU-A7** | ***Macrotermes gilvus*** | **PX517551** | **PX548770** | **/** | **PX608084** |
| ***Xylaria acuminatilongissima*** | **Thailand** | **CMU-A15** | ***Macrotermes gilvus*** | **PX517552** | **PX548771** | **PX608035** | **PX608085** |
| ***Xylaria acuminatilongissima*** | **Thailand** | **CMU-A17** | ***Macrotermes gilvus*** | **PX517553** | **PX548772** | **PX608036** | **PX608086** |
| ***Xylaria* sp.** | **Thailand** | **CMU-A8** | ***Macrotermes gilvus*** | **PX517554** | **PX548773** | **/** | **/** |
| ***Xylaria* sp.** | **Thailand** | **CMU-X16** | ***Macrotermes gilvus*** | **PX517555** | **PX548774** | **/** | **/** |
| ***Xylaria* sp.** | **Thailand** | **CMU-X1** | ***Macrotermes gilvus*** | **PX517556** | **PX548775** | **/** | **/** |
| ***Xylaria* sp.** | **Thailand** | **CMU-a10** | ***Macrotermes gilvus*** | **PX517557** | **PX548776** | **/** | **PX608087** |
| ***Xylaria* sp.** | **Thailand** | **CMU-a12** | ***Macrotermes gilvus*** | **PX517558** | **PX548777** | **PX608037** | **PX608088** |
| ***Xylaria neonigripes*** | **Thailand** | **CMU-a22** | ***Macrotermes gilvus*** | **PX517559** | **PX548778** | **PX608038** | **PX608089** |
| ***Xylaria siamensis*** | **Thailand** | **CMU-X10** | ***Macrotermes gilvus*** | **PX517560** | **PX548779** | **PX608039** | **PX608090** |
| ***Xylaria siamensis*** | **Thailand** | **CMU-A10** | ***Macrotermes gilvus*** | **PX517561** | **PX548780** | **PX608040** | **PX608091** |
| ***Xylaria siamensis*** | **Thailand** | **CMU-X46** | ***Macrotermes gilvus*** | **PX517562** | **PX548781** | **PX608041** | **PX608092** |
| ***Xylaria siamensis*** | **Thailand** | **CMU-a11** | ***Macrotermes gilvus*** | **PX517563** | **PX548782** | **PX608042** | **PX608093** |
| ***Xylaria* sp.** | **Thailand** | **CMU-X37** | ***Macrotermes gilvus*** | **PX517564** | **/** | **/** | **/** |
| ***Xylaria* sp.** | **Thailand** | **CMU-X33** | ***Macrotermes gilvus*** | **PX517565** | **/** | **/** | **/** |
| ***Xylaria* sp.** | **Thailand** | **CMU-A1** | ***Macrotermes gilvus*** | **PX517566** | **/** | **/** | **/** |
| ***Xylaria* sp.** | **Thailand** | **CMU-a19** | ***Macrotermes gilvus*** | **PX517567** | **/** | **/** | **/** |
| ***Xylaria* sp.** | **Thailand** | **CMU-a1** | ***Macrotermes gilvus*** | **PX517568** | **/** | **/** | **/** |
| ***Xylaria* sp.** | **Thailand** | **CMU-X49** | ***Macrotermes gilvus*** | **PX517569** | **/** | **/** | **/** |
| ***Xylaria* sp.** | **Thailand** | **CMU-X44** | ***Macrotermes gilvus*** | **PX517570** | **/** | **/** | **/** |
| ***Xylaria* sp.** | **Thailand** | **CMU-X27** | ***Macrotermes gilvus*** | **PX517571** | **/** | **/** | **/** |

**Table S8. Survival of** *Macrotermes* **workers and soldiers exposed to control treatments.** Values represent the survival percentages of termites (major workers+minor workers+major soldiers+minor soldiers)

|  | **0 h** | **24 h** | **48h** | **72 h** | **96 h** |
| --- | --- | --- | --- | --- | --- |
|  | **Survival** | **Survival** | **Survival** | **Survival** | **Survival** |
| **Treat1** | 10+10+2+2 (100%) | 10+10+2+2 (100%) | 10+10+2+2 (100%) | 10+10+2+2 (100%) | 10+10+2+2 (100%) |
| **Treat2** | 10+10+2+2 (100%) | 10+10+2+2 (100%) | 10+10+2+2 (100%) | 10+10+2+2 (100%) | 10+10+2+2 (100%) |
| **Treat3** | 10+10+2+2 (100%) | 10+10+1+2 (95.83%) | 10+10+1+2 (95.83%) | 10+10+1+2 (95.83%) | 10+10+1+2 (95.83%) |
| **Treat4** | 10+10+2+2 (100%) | 10+10+2+2 (100%) | 10+10+2+2 (100%) | 10+10+2+2 (100%) | 10+10+2+2 (100%) |
| **Treat5** | 10+10+2+2 (100%) | 10+10+2+2 (100%) | 10+10+2+2 (100%) | 10+10+2+2 (100%) | 10+10+2+2 (100%) |
| **Treat6** | 10+10+2+2 (100%) | 10+10+2+2 (100%) | 10+10+2+2 (100%) | 10+10+2+2 (100%) | 10+10+2+2 (100%) |
| **Treat7** | 10+10+2+2 (100%) | 10+9+2+2 (95.83%) | 10+9+2+2 (95.83%) | 10+9+2+2 (95.83%) | 10+9+2+2 (95.83%) |
| **Treat8** | 10+10+2+2 (100%) | 10+10+2+1 (95.83%) | 10+10+2+1 (95.83%) | 10+10+1+1 (91.67%) | 10+10+1+1 (91.67%) |
| **Treat9** | 10+10+2+2 (100%) | 10+10+2+2 (100%) | 10+10+2+2 (100%) | 10+10+1+2 (95.83%) | 10+10+1+2 (95.83%) |
| **Treat10** | 10+10+2+2 (100%) | 10+10+2+2 (100%) | 9+10+2+2 (95.83%) | 8+10+2+2 (91.67%) | 8+10+2+1 (87.5%) |

**Table S9. Survival of** *Macrotermes* **workers and soldiers exposed to** *Metarhizium* **treatments.** Values represent the survival percentages of termites (major workers+minor workers+major soldiers+minor soldiers)

|  | **0 h** | **24 h** | **48h** | **72 h** | **96 h** |
| --- | --- | --- | --- | --- | --- |
|  | **Survival** | **Survival** | **Survival** | **Survival** | **Survival** |
| **Treat1** | 10+10+2+2 (100%) | 10+9+2+2 (95.83%) | 5+4+1+1 (45.83%) | 0 (0%) | 0 (0%) |
| **Treat2** | 10+10+2+2 (100%) | 10+10+2+1 (95.83%) | 9+6+2+0 (70.83%) | 3+4+0+0 (29.17%) | 0 (0%) |
| **Treat3** | 10+10+2+2 (100%) | 10+10+2+1 (95.83%) | 7+8+2+0 (70.83%) | 3+5+1+0 (37.5%) | 0 (0%) |
| **Treat4** | 10+10+2+2 (100%) | 10+9+2+2 (95.83%) | 9+6+2+1 (75%) | 6+5+0+0 (45.83%) | 0 (0%) |
| **Treat5** | 10+10+2+2 (100%) | 10+9+2+2 (95.83%) | 8+6+2+0 (66.67%) | 6+1+1+0 (33.33%) | 0 (0%) |
| **Treat6** | 10+10+2+2 (100%) | 10+9+2+2 (95.83%) | 5+3+2+1 (45.83%) | 0 (0%) | 0 (0%) |
| **Treat7** | 10+10+2+2 (100%) | 10+8+2+2 (91.67%) | 9+5+0+0 (58.33%) | 6+1+0+0 (29.17%) | 0 (0%) |
| **Treat8** | 10+10+2+2 (100%) | 10+8+2+2 (91.67%) | 5+2+2+1 (41.67%) | 2+0+1+0 (12.5%) | 0 (0%) |
| **Treat9** | 10+10+2+2 (100%) | 10+9+2+1 (91.67%) | 9+3+1+0 (54.16%) | 5+0+0+0 (20.83%) | 0 (0%) |
| **Treat10** | 10+10+2+2 (100%) | 10+10+2+2 (100%) | 7+6+2+2 (70.83%) | 3+3+1+0 (29.17%) | 0 (0%) |

**Table S10. Weights of soil boluses deposited around each fungal plug (1.5-diameter paper circle) by *Macrotermes gilvus* termites under conditions with or without a fungus comb (mg).**

| **Plates** | **Absence of termite comb** | | | **Presence of termite comb** | | |
| --- | --- | --- | --- | --- | --- | --- |
|  | ***Xylaria* sp. plug** | **PDA plug** | ***Metarhizium* sp. plug** | ***Xylaria* sp. plug** | **PDA plug** | ***Metarhizium* sp. plug** |
| **1** | 71.1 | 69.6 | 193 | 91.6 | 89.6 | 172 |
| **2** | 70 | 60 | 270.6 | 81.8 | 75.3 | 140.6 |
| **3** | 50 | 66.9 | 143.2 | 57.7 | 44.4 | 137.5 |
| **4** | 91.3 | 61.6 | 117.6 | 91.3 | 61.6 | 217.6 |
| **5** | 89 | 51 | 101.2 | 79.4 | 59 | 123.8 |
| **6** | 52 | 61.6 | 110 | 11 | 72.1 | 314.2 |
| **7** | 95.2 | 82.1 | 119 | 122.3 | 81 | 299.9 |
| **8** | 79.5 | 71.28 | 132.5 | 81.2 | 77 | 160 |
| **9** | 61.2 | 64.2 | 152.2 | 52.5 | 44.1 | 122.1 |

**Table S11. Weights of soil boluses deposited around each fungal plug (1.5-diameter paper circle) by** Odontotermes sp. **termites under conditions with or without a fungus comb (mg).**

| **Plates** | **Absence of termite comb** | | | **Presence of termite comb** | | |
| --- | --- | --- | --- | --- | --- | --- |
|  | ***Xylaria* sp. plug** | **PDA plug** | ***Metarhizium* sp. plug** | ***Xylaria* sp. plug** | **PDA plug** | ***Metarhizium* sp. plug** |
| **1** | 51.2 | 22.3 | 76 | 75.1 | 35.5 | 109 |
| **2** | 61 | 35.1 | 146.2 | 30 | 14 | 197 |
| **3** | 42.1 | 20 | 113.2 | 26.5 | 33 | 191.5 |
| **4** | 32.2 | 48 | 115.3 | 60 | 41.3 | 188.8 |
| **5** | 72.1 | 39.9 | 102.4 | 37.7 | 50 | 92.1 |
| **6** | 36.3 | 40 | 75.2 | 61.9 | 45 | 180.2 |
| **7** | 84.3 | 50 | 126 | 56.8 | 34.2 | 102.2 |
| **8** | 19 | 22 | 71 | 82.2 | 39.9 | 160.8 |
| **9** | 95 | 30 | 197.2 | 53 | 40 | 101 |

**Table S12.** Results of pairwise comparisons using estimated marginal means (EM means) with Bonferroni correction in the GLM framework, in the presence or absence of termite comb (simultaneous weed exposure experiment).

| **Termite** | **Comb presence** | **Comparison** | **Mean difference** | **SE** | **Bonferroni-corrected P** | **95% CI** |
| --- | --- | --- | --- | --- | --- | --- |
| *M. gilvus* | Present | *Metarhizium* vs. *Xylaria* | 113.211 | 25.191 | 1.05 × 10^⁻4^ | [39.269, 187.153] |
|  |  | *Metarhizium* vs. PDA | 120.400 | 23.741 | 6 × 10^⁻6^ | [50.716, 190.084] |
|  |  | *Xylaria* vs. PDA | 7.189 | 11.005 | 1 | [-25.113, 39.490] |
|  | Absent | *Metarhizium* vs. *Xylaria* | 75.556 | 17.560 | 2.53 × 10^⁻4^ | [24.013, 127.099] |
|  |  | *Metarhizium* vs. PDA | 83.447 | 16.971 | 1.3 × 10^⁻5^ | [33.632, 133.261] |
|  |  | *Xylaria* vs. PDA | 7.891 | 5.929 | 1 | [-9.512, 25.294] |
| *Odontotermes* sp. | Present | *Metarhizium* vs. *Xylaria* | 89.933 | 14.561 | 9.836 × 10^⁻9^ | [49.195, 132.672] |
|  |  | *Metarhizium* vs. PDA | 106.633 | 13.632 | 7.827 × 10^⁻14^ | [66.621, 146.646] |
|  |  | *Xylaria* vs. PDA | 16.700 | 6.821 | 0.215 | [-3.321, 36.721] |
|  | Absent | *Metarhizium* vs. *Xylaria* | 58.811 | 14.977 | 1.292 × 10^⁻3^ | [14.850, 102.773] |
|  |  | *Metarhizium* vs. PDA | 79.467 | 13.172 | 2.415 × 10^⁻8^ | [40.804, 118.129] |
|  |  | *Xylaria* vs. PDA | 20.656 | 8.713 | 0.266 | [-4.920, 46.231] |

**Table S13.** Inhibition percentages (IP) of Metarhizium sp. Cmu-b60 and Xylaria sp. clade 20 isolates by 12 bacterial strains at day 15 and day 5. IP values ≥50% are indicated in bold.

| **Fungal isolate** | **Mean growth inhibition (GI) after 5/20 days ± SD (%)** | |
| --- | --- | --- |
|  | ***Metarhizium* sp. Cmu-b60** | ***Xylaria* sp. clade 20** |
| *Bacillus* CMU-g10 | **56.3%±3.4** | 0% |
| *Bacillus* CX-gut6 | **57.41%±5.01** | 0% |
| *Brevibacillus* CMU-C11 | 48.15%±2.56 | **54.44%±1.12** |
| *Brevibacillus* CX-gut6-1 | **57.78%±2.22** | 47.41%±2.8 |
| *Burkholderia* CMU-C7 | **51.11%±2.34** | **55.56%±1.1** |
| *Burkholderia* CX-Comb6 | 48.23%±2.24 | **54.44%±1.12** |
| *Enterobacter* CMU-g3 | 13.33%±2.23 | 0% |
| *Enterobacter* CX-gut10 | 8.89%±2.22 | 0% |
| *Pseudomonas* CMU-g13 | 44.44%±2.26 | **51.85%±1.62** |
| *Pseudomonas* CX-gut7 | 42.96%±3.4 | **57.41%±1.7** |
| *Serratia* CMU-g5 | 28.89%±5.88 | 49.63%±0.64 |
| *Serratia* CX-Comb4 | 26.67%±7.7 | **51.11%±5.56** |
